# Identification of Aerobic Salivary Microorganisms in Patients With Oral Squamous Cell Carcinoma Using MALDI‐TOF MS: Preliminary Findings From a Pilot Study

**DOI:** 10.1002/rcm.10063

**Published:** 2025-05-05

**Authors:** Milena da Silva Moreira, Giovanna Schmitz, Mariana de Sá Alves, Maria Anita Mendes, Mônica Ghislaine Oliveira Alves, Levy Anderson César Alves, Meriellen Dias, Celso Muller Bandeira, Gabrielle Luana Jimenez Teodoro Nepomuceno, Herculano da Silva Martinho, Janete Dias Almeida

**Affiliations:** ^1^ Universidade Estadual Paulista (Unesp), Instituto de Ciência e Tecnologia, Campus São José dos Campos, São José dos Campos São Paulo Brazil; ^2^ Dempster MS Lab Universidade de São Paulo São Paulo Brazil; ^3^ School of Dentistry Universidade Paulista São Paulo Brazil; ^4^ School of Dentistry Universidade Municipal de São Caetano do Sul São Caetano do Sul Brazil; ^5^ Centro de Ciências Naturais e Humanas Universidade Federal do ABC (UFABC) Santo André SP Brazil

**Keywords:** MALDI‐TOF MS, oral cancer, salivary microorganisms

## Abstract

**Rationale:**

Oral squamous cell carcinoma (OSCC) is a multifactorial disease, and emerging evidence links the oral microbiome to its development. Rapid, noninvasive identification of salivary microorganisms may offer novel diagnostic and prognostic insights into oral cancer.

**Methods:**

Salivary microbiota from OSCC patients and healthy individuals were analyzed using MALDI Biotyper. Saliva samples were cultured, and microbial identification was performed based on protein spectral profiles using an UltrafleXtreme MALDI‐TOF mass spectrometer.

**Results:**

Thirteen OSCC patients (mean age 55 ± 11 years; 69% male) and nineteen healthy controls (mean age 55 ± 10 years; 79% male) were analyzed. Distinct microbial profiles were observed in OSCC patients, including pathogenic species previously associated with carcinogenesis, suggesting potential biomarkers for oral cancer.

**Conclusions:**

The MALDI Biotyper is an effective, noninvasive tool for identifying salivary microbiota. Its application may support early diagnosis and prognosis of OSCC, reinforcing the significance of the oral microbiome in cancer etiology.

Oral squamous cell carcinoma (OSCC) directly impacts the lining of the oral mucosa. The National Cancer Institute (INCA) [1] estimated about 15 190 new cases of OSCC in Brazil in 2023, with 11 180 cases in men and 4010 cases in women.

The etiology of OSCC is multifactorial and risk factors include tobacco, excessive alcohol consumption, and exposure to ultraviolet solar radiation [2]. During the last decade, the etiology of oral cancer has been revised. Within this context, some investigations began associating certain pathogens present in the oral cavity with malignant neoplasms [3].

Research in microbiology has demonstrated an association between the occurrence of cancer and a significant increase in certain pathogens. Farrell et al. [4] observed variations in the oral microbiota, including an increase in the growth of the bacterium *Granulicatella adiacens* and a marked reduction in *Neisseria elongata* and *Streptococcus mitis* , in patients with pancreatic cancer. Chattopadhyay et al. [5] suggested specific oral pathogens to be potential risk factors for cancer development. These findings highlight the need for identifying diagnostic biomarkers, particularly oral microbial biomarkers, to enable early diagnosis and enhance treatment options.

Literature reports indicate that about 15% of malignant neoplasms are linked to microorganisms [6]. This is an innovative research field where, to the best of our knowledge, investigations have focused on the identification of microorganisms using molecular techniques based on 16S rRNA sequences. However, these methods are time‐consuming and expensive compared to high‐performance technologies like the MALDI Biotyper, a microbial identification system using matrix‐assisted laser desorption ionization/time‐of‐flight mass spectrometry (MALDI‐TOF MS) [7].

MALDI‐TOF MS is a widely used high‐throughput method [8]. Using this method, each bacterial species will provide the same mass spectrum. Identification is then performed by comparing the result of a bacterium with all other mass spectra available in the databases [8]. Its simplicity and speed thus render the method effective in detecting and characterizing several bacterial agents. Furthermore, MALDI‐TOF MS offers higher precision limits than techniques previously used to identify biomarkers present in bacteria, fungi, and viruses [9]. In addition, accurate results are obtained for more than 95% of isolated organisms, reducing the time for identifying these microorganisms to minutes compared to classical biochemistry [10]. Considering the speed of identification of the bacterial spectrum, the diagnostic process tends to be faster, enabling a better prognosis and therapeutic approaches for patients.

Research into the relationship between microorganisms and the development of malignant neoplasms is a promising field in science since it may lead to developing antineoplastic therapies for the population. Therefore, the present study aimed to identify aerobic salivary microorganisms in patients with OSCC using the MALDI Biotyper. The study was conducted by the Declaration of Helsinki and was approved by the Research Ethics Committee of the São José dos Campos Institute of Science and Technology (ICT‐UNESP) following the protocol described by Alves et al. [11]. MALDI‐TOF MS analysis was performed at the Dempster Mass Spectrometry Laboratory of the Polytechnic School of the University of São Paulo (Poli‐USP).

Patients older than 18 years with a diagnosis of OSCC were included. Patients who had already undergone some type of cancer treatment, that is, surgery, radiotherapy, and/or chemotherapy of any body part, were excluded.

Salivary samples were collected from 13 patients diagnosed with OSCC at the Municipal Mario Gatti Hospital and Celso Pierro Hospital. In addition, salivary samples were collected from a group of 19 healthy controls at ICT‐UNESP. The collection and storage of salivary samples followed the procedures specified in previous studies [11, 12].

## 1 Specimen Collection

Saliva was collected by sputum into a disposable plastic tube (Falcon, Rio de Janeiro) according to the modified method from Navazesh [13]. The participants were instructed to expectorate 3 mL of saliva into tubes that were kept on ice. The tubes were then closed and transported to the laboratory within a maximum of 1 h and stored in a freezer at −80°C.

## 2 Bacteria Isolation

For microbial identification using the MALDI Biotyper, a bacterial colony growth step is required to acquire spectra [11]. All samples were thawed at room temperature for subsequent bacterial isolation on brain–heart infusion agar (BHI; HiMedia Laboratories, Mumbai, India) incubated at 37°C for approximately 4 days [13], which allows morphological separation of the different bacteria. To ensure the purity of bacterial isolates, this process was repeated as needed until single colonies were obtained. On average, four different bacterial species were isolated from each primary agar plate.

## 3 MALDI‐TOF MS Analysis

The protocol was performed as follows: Bacterial cells from the BHI agar plate were transferred to a tube (Eppendorf, Germany) containing approximately 200 μL of Milli‐Q water. Next, 900 μL of ethanol was added, and the sample was centrifuged (13 000 rpm, 5 min). The supernatant was discarded after centrifugation, and ethanol was removed by repeated centrifugations. The pellet was resuspended in 50 μL of 70% formic acid. After this procedure, 50 μL of acetonitrile was added, and the sample was homogenized in a vortex and centrifuged again.

The α‐cyano‐4‐hydroxycinnamic acid matrix was prepared as a saturated solution of 50% acetonitrile and 2.5% trifluoroacetic acid. Next, 1 μL of the sample extract was dispensed onto a steel target plate and dried at room temperature. Finally, 1 μL of the matrix solution was added, and the plate was left to dry at room temperature. Mass spectrometry analyses were performed with the UltrafleXtreme MALDI‐TOF Mass Spectrometer (Bruker Daltonics, Germany) operating in the linear positive ion mode. Mass spectra were acquired in a mass range of 2 to 20 kDa, with ions formed by smart beam irradiation at a frequency of 2000 Hz, PIE of 100 ns, and a lens voltage of 7 kV. The acceleration voltages for the first and second ion sources were 25 and 23 kV, respectively. Bacteria were then identified using the Biotyper 3.1 Database. Cutoff values of 2 and 1.7 were used for species and genus identification, respectively.

Data were analyzed statistically using the Prism 5.03 program (GraphPad Inc., 2010). For descriptive statistics, the mean and standard deviation (SD) were calculated. The χ^2^‐test was used to check for significant differences in the bacteria identified between the groups studied. A level of significance of 5% was considered in all analyses.

## 4 Results

Thirteen salivary samples from OSCC patients were selected for the study, including nine men (69%) and four women (31%), as summarized in Table 1. The ages of these patients ranged from 35 to 71 years (mean ± SD: 55 ± 11 years). The control group consisted of 19 healthy individuals, including 15 men (79%) and four women (21%) (Table 1), whose age ranged from 32 to 71 years (55 ± 10 years). All patients underwent extraoral and intraoral examinations and completed a questionnaire regarding their smoking habits. Additionally, patients with OSCC were categorized based on the stage of the disease, as shown in Table 1.

**TABLE 1:** Distribution of demographic data.

	OSCC group (*n* = 13) *n* (%)	Control group (*n* = 19) *n* (%)
**Sex**		
Male	9 (69%)	15 (79%)
Female	4 (31%)	4 (21%)
**Smoking habit**		
Smoker	10 (77%)	2 (10%)
Nonsmoker	3 (23%)	10 (53%)
Former smoker		7 (37%)
**OSCC stage**		
I	3 (23%)	
II	2 (15%)	
III	1 (8%)	
IV	7 (54%)	

Abbreviation: OSCC, oral squamous cell carcinoma.

The data presented in Table 1 aim to characterize the study population. It is important to note that the population with OSCC largely overlaps with those described in previous studies, which predominantly consisted of male smokers [14].

Table 2 shows the results of salivary microbial identification obtained for the OSCC and control groups using the MALDI Biotyper. We followed Bruker's guidelines for scoring. According to Bruker, scores below 1.7 do not allow the identification of microorganisms at the genus or species levels. Therefore, all data with scores below 1.7 were not included in our analysis. Scores between 2.0 and 2.29 allow the probable identification of microbiological species in the salivary sample, while scores between 1.7 and 1.99 enable the identification of the probable microbiological genus but not the species. Thus, in Table 2, under the “Bruker criteria” column, we indicate which microorganisms were identified at the probable species level and which were identified only at the probable genus level.

**TABLE 2:** Microbial species identified in the saliva of OSCC patients and healthy controls.

Microorganisms identified	OSCC group *n* (%)	Control group *n* (%)	*p* *	Bruker criteria
*Arthrobacter gandavensis*	1 (8%)	—	0.2193	Specie
*Candida albicans*	1 (8%)	—	0.2193	Genus
*Enterococcus faecalis*	1 (8%)	—	0.2193	Specie
*Filifactor villosus*	1 (8%)	—	0.2193	Genus
*Rothia aeria*	—	5 (26%)	**0.0441** *	Genus
*Rothia dentocariosa*	3 (23%)	11 (58%)	0.0512	Specie
*Rothia mucilaginosa*	4 (31%)	4 (21%)	0.5333	Specie
*Staphylococcus aureus*	3 (23%)	1 (5%)	0.1345	Genus
*Streptococcus salivarius*	—	7 (37%)	**0.0133** *	Genus
*Streptococcus vestibularis*	1 (8%)	—	0.2193	Genus

Abbreviation: OSCC, oral squamous cell carcinoma.

* Chi‐square (χ^2^) test considering *p*≤0.05.

In the OSCC group, the most prevalent microorganism was *Rothia mucilaginosa* (31%), followed by genus *Staphylococcus* (23%) and the species *Rothia dentocariosa* (23%). Additionally, five other microorganisms were identified: the genera *Candida* (8%), *Filifactor* (8%), and *Streptococcus* (8%), as well as the *species Enterococcus faecalis* (8%) and *Arthrobacter gandavensis* (8%) e *genus*, totaling eight microbial taxa identified in the OSCC group.

In the control group, the most prevalent microorganisms in saliva were *R. dentocariosa* (58%), the genus *Streptococcus* (37%), the genus *Rothia* (26%), and the species *Rothia mucilaginosa* (21%). Notably, the prevalence of the genus *Staphylococcus* was only 5%. Five microorganisms were not detected in the saliva of these individuals considered healthy: the species *A. gandavensis, E. faecalis, as well as the genera Streptococcus, Candida, and Filifactor*. Notably, two genera identified in the control group, *Rothia* and *Streptococcus*, were absent in the saliva of OSCC patients. Conversely, two species, *R. mucilaginosa* and *R. dentocariosa* , along with the genus *Staphylococcus*, were detected in both groups.

A statistically significant difference was observed between the OSCC and control groups for the genera *Rothia* (*p* = 0.441) and *Streptococcus* (*p* = 0.0133), highlighting the relevance of these genera in the control group. However, no statistically significant differences were found for the other microorganisms identified using the MALDI Biotyper.

It is important to note that the ***n*** shown in Table 2 below represents the number of patients with the microorganism present in their saliva. For example, if the **n** for *R. mucilaginosa* in the OSCC group is four, this indicates that four patients with the diagnosis of OSCC also had this microorganism in their saliva.

## 5 Discussion

The primary objective of this study was to identify aerobic salivary microorganisms in patients diagnosed with OSCC using the MALDI‐TOF MS technique. The MALDI Biotyper can identify the protein composition of microbial cells, making it a widely adopted technique in routine laboratories for practical, fast, reliable, and cost‐effective identification of microbial species [15, 16, 17, 18, 19]. Some studies investigating the salivary microbiota of patients with OSCC [20, 21, 22, 23] have used other identification techniques such as PCR. The present study is therefore the first to employ the MALDI‐TOF MS technique.

The main risk factors for the development of OSCC are alcohol and tobacco [24]. Tobacco smoke exerts deleterious effects on the mouth caused by carcinogenic substances such as nitrosamines, heavy metal ions, phenols, and aromatic hydrocarbons. The substances promote changes in oral tissues that can lead to the development of oral cancer [25]. The habit of smoking has been associated with OSCC [24], corroborating the results of the present study in which the majority of OSCC patients were smokers. Cigarette smoking can affect health by creating a different environment in the oral cavity that interferes with the complex relationships between bacteria and by altering certain metabolic pathways [26]. The importance of the microbiome for the development of different oral pathologies, including OSCC, is notorious since immunocompromised patients have disorganized microbiomes when compared to healthy patients [26].

In 2005, a North American study suggested that three bacterial species, *Capnocytophaga gingivalis* , *Prevotella melaninogenica* , and *S. mitis* , could serve as potential noninvasive diagnostic indicators of oral cancer because of their high counts in the saliva of these patients [27]. However, our study did not identify these three species.

In our study, *Filifactor* was found to be prevalent in patients with oral cancer. These genera bacteria have been identified in other studies and have been reported to induce the secretion of proinflammatory cytokines, activating specific oncogenes and maintaining the environment in an inflammatory state. Additionally, this species increases tumor progression by promoting tumor colonization by other pathogens [5, 28]. Furthermore, *Filifactor* has been identified as a potential microbial biomarker in patients with primary bronchogenic carcinoma [29].

*E. faecalis* is a symbiotic lactic acid bacterium involved in biofilm formation and present in the gastrointestinal tract [30]. This species has been frequently associated with the development of colorectal cancer. Studies suggest that *E. faecalis* can damage the deoxyribonucleic acid (DNA) of colon epithelial cells, leading to the development of malignant neoplasms [31]. Few studies have evaluated this species in the mouth and its potential relationship with the development of OSCC.

*Rothia aeria* , *R. dentocariosa* , and *R. mucilaginosa* are species commonly reported as part of the normal oral cavity flora, generally not associated with harmful effects [32]. This finding supports our results, as a higher number of these microorganisms were observed in the control group. Furthermore, in immunocompromised patients, it has been observed that these microorganisms of the *Rothia* genus, which are natural inhabitants of the oral flora, can become pathogenic and infect the oral microenvironment due to oral dysbiosis [32].

The genus *Rothia* is associated with the production of carcinogenic acetaldehyde, and an increase in these bacteria in OSCC has been previously reported [5]. *R. dentocariosa, R. mucilaginosa*, and the genus *Staphylococcus* were detected in both the OSCC and control groups. *R. dentocariosa* is a gram‐positive bacterium that inhabits the oral cavity and has been frequently identified in studies on squamous cell carcinoma, where its prevalence was found to be higher in control groups compared to those with malignant neoplasms [33]. Additionally, studies investigating the presence of *R. mucilaginosa* in oral leukoplakia lesions have shown an increase in this species in potentially malignant lesions [34].

*Staphylococcus aureus* is recognized as a bacterium with a significant potential to cause chronic infections and bacteremia, especially in immunocompromised individuals [35, 36]. The prevalence of the genus *Staphylococcus* was notably higher in the OSCC group compared to the control group. *Streptococcus salivarius*, on the other hand, is regarded as a probiotic agent of the oral cavity [37]. Many oral bacteria within the *Streptococcus* genus produce lactic acid, which acts as an immunosuppressive mediator, making it a valuable target for cancer therapies [38, 39]. It is known that a low pH and hypoxic environment favor tumor metastases [40].

Species within the genus *Candida* are considered opportunistic pathogens, meaning they can shift from being harmless commensals to harmful pathogens based on the host's immune defense status. These microorganisms have been reported to be involved in oral carcinogenesis [41]. *Candida albicans* is capable of inducing carcinogenesis through the release of proinflammatory cytokines that trigger a Th17 response and through direct carcinogens [42], in addition to TNF‐α and IL‐18, which are associated with tumor invasiveness, migration, and metastasis [43]. Furthermore, *C. albicans* can produce nitrosamines that lead to the activation of proto‐oncogenes involved in the formation of cancerous lesions [41].

We acknowledge the limitations of our study, including the small sample size of 13 cancer patients and the limited number of microorganisms identified. Therefore, we cannot definitively establish these microorganisms as biomarkers for oral cancer. However, we demonstrate that MALDI Biotyper proved to be an efficient, noninvasive, rapid, and cost‐effective method for identifying salivary microorganisms in OSCC patients, marking a pioneering approach in this field.

## 6 Conclusion

Despite the limitations of our study, the microbial species identified here have the potential to serve as future biomarkers for the diagnosis and prognosis of oral cancer. Furthermore, our study revealed the presence of pathogenic microorganisms in the saliva of patients with OSCC, compared to individuals without the disease. Some of the microbial species identified have already been associated with carcinogenesis in other studies and could be further explored in future research.

## Author Contributions

**Milena da Silva Moreira:** investigation, writing – original draft, funding acquisition, resources, data curation, methodology, visualization. **Giovanna Schmitz:** data curation, resources, methodology, visualization, investigation, funding acquisition, writing – original draft. **Mariana de Sá Alves:** investigation, funding acquisition, writing – original draft, methodology, visualization, writing – review and editing, software, supervision. **Maria Anita Mendes:** methodology, supervision, software, project administration, conceptualization, investigation, validation, data curation, resources, writing – original draft, writing – review and editing, visualization. **Mônica Ghislaine Oliveira Alves:** writing – review and editing, writing – original draft, data curation, resources. **Levy Anderson César Alves:** conceptualization, software, data curation, supervision. **Meriellen Dias:** conceptualization, methodology, software, data curation, supervision. **Celso Muller Bandeira:** investigation. **Gabrielle Luana Jimenez Teodoro Nepomuceno:** investigation. **Herculano da Silva Martinho:** writing – original draft, conceptualization, funding acquisition, supervision, writing – review and editing. **Janete Dias Almeida:** conceptualization, writing – review and editing, project administration, funding acquisition, supervision.

## Conflicts of Interest

The authors declare no conflicts of interest.

### Peer Review

The peer review history for this article is available at https://www.webofscience.com/api/gateway/wos/peer‐review/10.1002/rcm.10063.

## Supporting information


**Data S1.** Supporting Information.

**Data S2.** Supporting Information.

## Data Availability

Research data are not shared.
