# Supplementary material for: Identification of Aerobic Salivary Microorganisms in Patients With Oral Squamous Cell Carcinoma Using MALDI‐TOF MS: Preliminary Findings From a Pilot Study
Source: Rapid Commun Mass Spectrom. 2025 May 5;39(15):e10063. doi: 10.1002/rcm.10063 (PMC12050990; doi:10.1002/rcm.10063)
Supplement: Supplementary file 1 — Data S1. Supporting Information. [file RCM-39-e10063-s001.pdf]

Bruker Daltonik MALDI Biotyper

Classification Results

Project Info:

Project Name:	**UNESP_03_12_2019**
Project Description:	
Project Owner:	Anita
Project Creation Date/Time:	2019-12-04T12:25:45.351
Project Analyte Count:	220
Project Type:	Development
Validation:	not present
Validation Position:	

Result Overview

Analyte Name	Analyte ID	Organism (best match)	Score Value	Organism (second best match)	Score Value
A1 (-) (C)	1	**not reliable identification**	1.498	**not reliable identification**	1.444
A2 (-) (C)	1	**not reliable identification**	1.506	**not reliable identification**	1.501
A3 (-) (C)	2	**not reliable identification**	1.422	**not reliable identification**	1.391
A4 (-) (C)	2	**not reliable identification**	1.376	**not reliable identification**	1.375
A5 (-) (C)	3	**not reliable identification**	1.428	**not reliable identification**	1.414
A6 (-) (C)	3	**not reliable identification**	1.438	**not reliable identification**	1.427
A7 (-) (C)	4	**not reliable identification**	1.449	**not reliable identification**	1.389
A8 (-) (C)	4	**not reliable identification**	1.517	**not reliable identification**	1.455
A9 (-) (C)	5	**not reliable identification**	1.583	**not reliable identification**	1.575

A10<br>**(-) (C)**	5	**not reliable identification**	1.641	**not reliable identification**	1.621
A11<br>**(-) (C)**	6	**not reliable identification**	1.423	**not reliable identification**	1.383
A12<br>**(-) (C)**	6	**not reliable identification**	1.409	**not reliable identification**	1.37
A13<br>**(-) (C)**	7	**not reliable identification**	1.382	**not reliable identification**	1.374
A14<br>**(-) (C)**	7	**not reliable identification**	1.409	**not reliable identification**	1.358
A15<br>**(-) (C)**	8	**not reliable identification**	1.434	**not reliable identification**	1.366
A16<br>**(-) (C)**	8	**not reliable identification**	1.439	**not reliable identification**	1.35
A17<br>**(-) (C)**	9	**not reliable identification**	1.403	**not reliable identification**	1.333
A18<br>**(-) (C)**	9	**not reliable identification**	1.402	**not reliable identification**	1.377
A19<br>**(-) (C)**	10	**not reliable identification**	1.394	**not reliable identification**	1.366
A20<br>**(-) (C)**	10	**not reliable identification**	1.437	**not reliable identification**	1.384
A21<br>**(-) (C)**	11	**not reliable identification**	1.527	**not reliable identification**	1.338
A22<br>**(-) (C)**	11	**not reliable identification**	1.462	**not reliable identification**	1.398
A23<br>**(++) (A)**	1A	Rothia mucilaginosa	2.089	Rothia mucilaginosa	2.084
A24<br>**(+) (B)**	1A	Rothia mucilaginosa	1.922	Rothia mucilaginosa	1.802
B1<br>**(-) (C)**	1B	**not reliable identification**	1.52	**not reliable identification**	1.447
B2<br>**(-) (C)**	1B	**not reliable identification**	1.501	**not reliable identification**	1.375
B3<br>**(-) (C)**	2A	**not reliable identification**	1.432	**not reliable identification**	1.404
B4<br>**(-) (C)**	2A	**not reliable identification**	1.697	**not reliable identification**	1.528
B5<br>**(+) (B)**	2B	Streptococcus vestibularis	1.788	Streptococcus vestibularis	1.74

B6<br>(-) (**C**)	2B	**not reliable identification**	1.551	**not reliable identification**	1.476
B7<br>(++) (**A**)	3A	Rothia mucilaginosa	2.032	Rothia mucilaginosa	1.942
B8<br>(+++) (**A**)	3A	Rothia mucilaginosa	2.37	**not reliable identification**	1.697
B9<br>(+) (**B**)	3B	Rothia mucilaginosa	1.95	Rothia mucilaginosa	1.807
B10<br>(++) (**A**)	3B	Rothia mucilaginosa	2.105	Rothia dentocariosa	1.769
B11<br>(-) (**C**)	4A	**not reliable identification**	1.561	**not reliable identification**	1.482
B12<br>(+) (**B**)	4A	Staphylococcus aureus	1.755	**not reliable identification**	1.548
B13<br>(-) (**C**)	4B	**not reliable identification**	1.666	**not reliable identification**	1.62
B14<br>(+) (**B**)	4B	Staphylococcus aureus	1.722	**not reliable identification**	1.554
B15<br>(-) (**C**)	5A	**not reliable identification**	1.4	**not reliable identification**	1.392
B16<br>(-) (**C**)	5A	**not reliable identification**	1.364	**not reliable identification**	1.343
B17<br>(+++) (**A**)	5B	Enterococcus faecalis	2.372	Enterococcus faecalis	2.075
B18<br>(+++) (**A**)	5B	Enterococcus faecalis	2.469	Enterococcus faecalis	2.166
B19<br>(-) (**C**)	7A	**not reliable identification**	1.374	**not reliable identification**	1.25
B20<br>(-) (**C**)	7A	**not reliable identification**	1.482	**not reliable identification**	1.289
B21<br>(++) (**A**)	7B	Rothia dentocariosa	2.162	Rothia dentocariosa	1.981
B22<br>(+) (**B**)	7B	Rothia dentocariosa	1.996	Rothia dentocariosa	1.767
B23<br>(-) (**C**)	9A	**not reliable identification**	1.646	**not reliable identification**	1.588
B24<br>(+) (**B**)	9A	Staphylococcus aureus	1.829	**not reliable identification**	1.499
C1<br>(-) (**C**)	9B	**not reliable identification**	1.37	**not reliable identification**	1.321

C2<br>**(+) (B)**	9B	Staphylococcus aureus	1.977	Staphylococcus aureus	1.794
C3<br>**(-) (C)**	10A	**not reliable identification**	1.51	**not reliable identification**	1.452
C4<br>**(-) (C)**	10A	**not reliable identification**	1.494	**not reliable identification**	1.429
C5<br>**(-) (C)**	10B	**not reliable identification**	1.391	**not reliable identification**	1.273
C6<br>**(-) (C)**	10B	**not reliable identification**	1.394	**not reliable identification**	1.394
C7<br>**(+) (B)**	16A	Candida albicans	1.924	Candida albicans	1.899
C8<br>**(-) (C)**	16A	**not reliable identification**	1.659	**not reliable identification**	1.658
C9<br>**(+) (B)**	16B	Filifactor villosus	1.72	**not reliable identification**	1.415
C10<br>**(-) (C)**	16B	**not reliable identification**	1.436	**not reliable identification**	1.4
C11<br>**(-) (C)**	17	**not reliable identification**	1.556	**not reliable identification**	1.553
C12<br>**(-) (C)**	17	**not reliable identification**	1.534	**not reliable identification**	1.414
C13<br>**(-) (C)**	19	**not reliable identification**	1.39	**not reliable identification**	1.383
C14<br>**(-) (C)**	19	**not reliable identification**	1.523	**not reliable identification**	1.511
C15<br>**(-) (C)**	20	**not reliable identification**	1.459	**not reliable identification**	1.409
C16<br>**(-) (C)**	20	**not reliable identification**	1.526	**not reliable identification**	1.488
C17<br>**(-) (C)**	21	**not reliable identification**	1.617	**not reliable identification**	1.507
C18<br>**(-) (C)**	21	**no peaks found**	< 0	**no peaks found**	< 0
C19<br>**(-) (C)**	24	**not reliable identification**	1.544	**not reliable identification**	1.458
C20<br>**(-) (C)**	24	**not reliable identification**	1.56	**not reliable identification**	1.443
C21<br>**(-) (C)**	25A	**not reliable identification**	1.604	**not reliable identification**	1.507

C22 (-) (C)	25A	**not reliable identification**	1.359	**not reliable identification**	1.359
C23 (+) (B)	25B	Rothia dentocariosa	1.838	**not reliable identification**	1.592
C24 (+) (B)	25B	Rothia dentocariosa	1.757	**not reliable identification**	1.625
D1 (-) (C)	26A	**not reliable identification**	1.449	**not reliable identification**	1.391
D2 (-) (C)	26A	**not reliable identification**	1.529	**not reliable identification**	1.501
D3 (-) (C)	26B	**not reliable identification**	1.438	**not reliable identification**	1.422
D4 (-) (C)	26B	**not reliable identification**	1.504	**not reliable identification**	1.455
D5 (-) (C)	27A	**not reliable identification**	1.652	**not reliable identification**	1.495
D6 (-) (C)	27A	**not reliable identification**	1.475	**not reliable identification**	1.452
D7 (+) (B)	27B	Rothia mucilaginosa	1.768	Rothia mucilaginosa	1.708
D8 (++) (A)	27B	Rothia mucilaginosa	2.046	Rothia mucilaginosa	1.938
D9 (-) (C)	30A	**not reliable identification**	1.46	**not reliable identification**	1.403
D10 (-) (C)	30A	**not reliable identification**	1.457	**not reliable identification**	1.433
D11 (-) (C)	30B	**not reliable identification**	1.509	**not reliable identification**	1.472
D12 (-) (C)	30B	**not reliable identification**	1.482	**not reliable identification**	1.389
D13 (++) (A)	31	Rothia dentocariosa	2.047	Rothia dentocariosa	1.983
D14 (++) (A)	31	Rothia dentocariosa	2.12	Rothia mucilaginosa	1.965
D15 (-) (C)	32	**not reliable identification**	1.698	**not reliable identification**	1.556
D16 (-) (C)	32	**not reliable identification**	1.455	**not reliable identification**	1.436
D17 (++) (A)	33A	Rothia mucilaginosa	2.115	Rothia dentocariosa	1.812

D18<br>**(++) (A)**	33A	Rothia mucilaginosa	2.298	Rothia mucilaginosa	2.044
D19<br>**(-) (C)**	33B	**not reliable identification**	1.503	**not reliable identification**	1.493
D20<br>**(-) (C)**	33B	**not reliable identification**	1.646	**not reliable identification**	1.624
D21<br>**(+) (B)**	35A	Staphylococcus aureus	1.705	**not reliable identification**	1.474
D22<br>**(-) (C)**	35A	**not reliable identification**	1.49	**not reliable identification**	1.448
D23<br>**(-) (C)**	35B	**not reliable identification**	1.478	**not reliable identification**	1.429
D24<br>**(-) (C)**	35B	**not reliable identification**	1.616	**not reliable identification**	1.484
E1<br>**(-) (C)**	37A	**not reliable identification**	1.516	**not reliable identification**	1.383
E2<br>**(-) (C)**	37A	**not reliable identification**	1.393	**not reliable identification**	1.317
E3<br>**(-) (C)**	37B	**not reliable identification**	1.528	**not reliable identification**	1.48
E4<br>**(-) (C)**	37B	**not reliable identification**	1.466	**not reliable identification**	1.428
E5<br>**(-) (C)**	37C	**not reliable identification**	1.415	**not reliable identification**	1.392
E6<br>**(-) (C)**	37C	**not reliable identification**	1.456	**not reliable identification**	1.374
E7<br>**(-) (C)**	39A	**not reliable identification**	1.363	**not reliable identification**	1.362
E8<br>**(-) (C)**	39A	**not reliable identification**	1.549	**not reliable identification**	1.509
E9<br>**(-) (C)**	39B	**not reliable identification**	1.48	**not reliable identification**	1.423
E10<br>**(-) (C)**	39B	**not reliable identification**	1.537	**not reliable identification**	1.519
E11<br>**(-) (C)**	39C	**not reliable identification**	1.605	**not reliable identification**	1.595
E12<br>**(-) (C)**	39C	**not reliable identification**	1.501	**not reliable identification**	1.427
E13<br>**(++) (A)**	40A	Rothia dentocariosa	2.094	Rothia mucilaginosa	1.942

E14 (++) (A)	40A	Rothia dentocariosa	2.022	**not reliable identification**	1.571
E15 (-) (C)	40B	**not reliable identification**	1.5	**not reliable identification**	1.426
E16 (-) (C)	40B	**not reliable identification**	1.475	**not reliable identification**	1.425
E17 (++) (A)	40C	Arthrobacter gandavensis	2.223	**not reliable identification**	1.556
E18 (++) (A)	40C	Arthrobacter gandavensis	2.193	**not reliable identification**	1.568
E19 (+) (B)	CN19A	Rothia dentocariosa	1.723	**not reliable identification**	1.498
E20 (+) (B)	CN19A	Rothia dentocariosa	1.717	**not reliable identification**	1.671
E21 (+) (B)	CN19B	Streptococcus salivarius	1.825	**not reliable identification**	1.549
E22 (+) (B)	CN19B	Streptococcus salivarius	1.911	**not reliable identification**	1.635
E23 (+) (B)	C30A	Rothia aeria	1.987	Rothia aeria	1.832
E24 (+) (C)	C30A	not reliable identification	1.7	**not reliable identification**	1.506
F1 (-) (C)	C30B	**not reliable identification**	1.68	**not reliable identification**	1.448
F2 (-) (C)	C30B	**not reliable identification**	1.432	**not reliable identification**	1.385
F3 (-) (C)	CN26A	**not reliable identification**	1.569	**not reliable identification**	1.511
F4 (-) (C)	CN26A	**not reliable identification**	1.515	**not reliable identification**	1.464
F5 (+) (B)	CN26B	Rothia dentocariosa	1.839	**not reliable identification**	1.608
F6 (-) (C)	CN26B	**not reliable identification**	1.44	**not reliable identification**	1.373
F7 (+) (B)	CN15A	Streptococcus salivarius	1.921	Streptococcus vestibularis	1.762
F8 (+) (B)	CN15A	Streptococcus salivarius	1.829	**not reliable identification**	1.577
F9 (-) (C)	CN15B	**not reliable identification**	1.539	**not reliable identification**	1.508

F10 **(-) (C)**	CN15B	**not reliable identification**	1.454	**not reliable identification**	1.417
F11 **(-) (C)**	CN3A	**not reliable identification**	1.682	**not reliable identification**	1.595
F12 **(+) (B)**	CN3A	Rothia aeria	1.819	**not reliable identification**	1.637
F13 **(-) (C)**	CN3B	**not reliable identification**	1.614	**not reliable identification**	1.419
F14 **(+) (B)**	CN3B	Rothia dentocariosa	1.879	Rothia dentocariosa	1.843
F15 **(+) (B)**	C5A	Rothia aeria	1.81	**not reliable identification**	1.594
F16 **(-) (C)**	C5A	**not reliable identification**	1.363	**not reliable identification**	1.362
F17 **(++) (A)**	C5B	Rothia dentocariosa	2.128	Rothia dentocariosa	1.866
F18 **(+) (B)**	C5B	Rothia dentocariosa	1.896	**not reliable identification**	1.668
F19 **(+) (B)**	C5C	Rothia dentocariosa	1.917	**not reliable identification**	1.511
F20 **(+) (B)**	C5C	Rothia dentocariosa	1.841	**not reliable identification**	1.464
F21 **(+) (B)**	CN13	Rothia mucilaginosa	1.993	Rothia mucilaginosa	1.736
F22 **(++) (A)**	CN13	Rothia mucilaginosa	2.156	Rothia mucilaginosa	2.016
F23 **(-) (C)**	CN4A	**not reliable identification**	1.668	**not reliable identification**	1.658
F24 **(+) (B)**	CN4A	Rothia dentocariosa	1.763	Rothia dentocariosa	1.723
G1 **(+) (B)**	CN4B	Rothia dentocariosa	1.762	Rothia dentocariosa	1.738
G2 **(-) (C)**	CN4B	**not reliable identification**	1.461	**not reliable identification**	1.455
G3 **(-) (C)**	CN5A	**not reliable identification**	1.477	**not reliable identification**	1.474
G4 **(-) (C)**	CN5A	**not reliable identification**	1.578	**not reliable identification**	1.511
G5 **(-) (C)**	CN5B	**not reliable identification**	1.599	**not reliable identification**	1.594

G6<br>(+) (**B**)	CN5B	Rothia dentocariosa	1.741	**not reliable identification**	1.492
G7<br>(-) (**C**)	CN18A	**not reliable identification**	1.417	**not reliable identification**	1.404
G8<br>(+) (**B**)	CN18A	Rothia aeria	1.815	Rothia aeria	1.749
G9<br>(-) (**C**)	CN18B	**not reliable identification**	1.61	**not reliable identification**	1.583
G10<br>(+) (**B**)	CN18B	Rothia dentocariosa	1.733	**not reliable identification**	1.604
G11<br>(-) (**C**)	CN32A	**not reliable identification**	1.444	**not reliable identification**	1.356
G12<br>(-) (**C**)	CN32A	**not reliable identification**	1.576	**not reliable identification**	1.552
G13<br>(-) (**C**)	CN32B	**not reliable identification**	1.452	**not reliable identification**	1.284
G14<br>(-) (**C**)	CN32B	**not reliable identification**	1.421	**not reliable identification**	1.399
G15<br>(-) (**C**)	CN32C	**not reliable identification**	1.41	**not reliable identification**	1.398
G16<br>(-) (**C**)	CN32C	**not reliable identification**	1.494	**not reliable identification**	1.343
G17<br>(-) (**C**)	CN20A	**not reliable identification**	1.355	**not reliable identification**	1.327
G18<br>(-) (**C**)	CN20A	**not reliable identification**	1.588	**not reliable identification**	1.502
G19<br>(-) (**C**)	CN20B	**not reliable identification**	1.553	**not reliable identification**	1.331
G20<br>(-) (**C**)	CN20B	**not reliable identification**	1.401	**not reliable identification**	1.339
G21<br>(-) (**C**)	CN21A	**not reliable identification**	1.294	**not reliable identification**	1.273
G22<br>(+) (**B**)	CN21A	Rothia mucilaginosa	1.804	Rothia mucilaginosa	1.732
G23<br>(++) (**A**)	CN21B	Rothia dentocariosa	2.095	Rothia dentocariosa	2.033
G24<br>(-) (**C**)	CN21B	**not reliable identification**	1.556	**not reliable identification**	1.466
H1<br>(-) (**C**)	CN21C	**not reliable identification**	1.56	**not reliable identification**	1.482

H2<br>**(-) (C)**	CN21C	**not reliable identification**	1.699	**not reliable identification**	1.534
H3<br>**(-) (C)**	CN2A	**not reliable identification**	1.391	**not reliable identification**	1.331
H4<br>**(-) (C)**	CN2A	**not reliable identification**	1.362	**not reliable identification**	1.356
H5<br>**(-) (C)**	CN2B	**not reliable identification**	1.54	**not reliable identification**	1.436
H6<br>**(+) (B)**	CN2B	Rothia mucilaginosa	1.8	**not reliable identification**	1.693
H7<br>**(-) (C)**	CN2C	**not reliable identification**	1.695	**not reliable identification**	1.42
H8<br>**(-) (C)**	CN2C	**not reliable identification**	1.269	**not reliable identification**	1.229
H9<br>**(+) (B)**	CN25A	Streptococcus salivarius	1.827	**not reliable identification**	1.452
H10<br>**(-) (C)**	CN25A	**not reliable identification**	1.532	**not reliable identification**	1.428
H11<br>**(+) (B)**	CN25B	Streptococcus salivarius	1.861	Streptococcus salivarius	1.793
H12<br>**(+) (B)**	CN25B	Streptococcus salivarius	1.981	**not reliable identification**	1.592
H13<br>**(+) (B)**	C33	Streptococcus salivarius	1.765	**not reliable identification**	1.603
H14<br>**(+) (B)**	C33	Streptococcus salivarius	1.836	Streptococcus salivarius	1.772
H15<br>**(-) (C)**	CN23A	**not reliable identification**	1.555	**not reliable identification**	1.437
H16<br>**(-) (C)**	CN23A	**not reliable identification**	1.381	**not reliable identification**	1.373
H17<br>**(-) (C)**	CN23B	**not reliable identification**	1.448	**not reliable identification**	1.432
H18<br>**(-) (C)**	CN23B	**not reliable identification**	1.444	**not reliable identification**	1.439
H19<br>**(+) (B)**	CN6A	Rothia aeria	1.868	**not reliable identification**	1.58
H20<br>**(-) (C)**	CN6A	**not reliable identification**	1.496	**not reliable identification**	1.49
H21<br>**(+) (B)**	CN6B	Streptococcus salivarius	1.951	Streptococcus salivarius	1.823

H22 (-) (C)	CN6B	**not reliable identification**	1.668	**not reliable identification**	1.568
H23 (-) (C)	CN10A	**not reliable identification**	1.378	**not reliable identification**	1.333
H24 (-) (C)	CN10A	**not reliable identification**	1.631	**not reliable identification**	1.495
I1 (-) (C)	CN10B	**not reliable identification**	1.528	**not reliable identification**	1.519
I2 (+) (B)	CN10B	Streptococcus salivarius	1.731	**not reliable identification**	1.636
I3 (-) (C)	CN31	**not reliable identification**	1.464	**not reliable identification**	1.399
I4 (-) (C)	CN31	**not reliable identification**	1.456	**not reliable identification**	1.425
I5 (-) (C)	CN11A	**not reliable identification**	1.542	**not reliable identification**	1.442
I6 (++) (A)	CN11A	Staphylococcus aureus	2.108	Staphylococcus aureus	1.766
I7 (-) (C)	CN11B	**not reliable identification**	1.693	**not reliable identification**	1.373
I8 (+) (B)	CN11B	Rothia dentocariosa	1.811	**not reliable identification**	1.235
I9 (+) (B)	CN11C	Rothia dentocariosa	1.793	**not reliable identification**	1.598
I10 (-) (C)	CN11C	**not reliable identification**	1.48	**not reliable identification**	1.405
I11 (-) (C)	CN11D	**not reliable identification**	1.392	**not reliable identification**	1.39
I12 (-) (C)	CN11D	**not reliable identification**	1.364	**not reliable identification**	1.308
I13 (-) (C)	C35	**not reliable identification**	1.452	**not reliable identification**	1.444
I14 (++) (A)	C35	Streptococcus salivarius	2.093	Streptococcus vestibularis	1.773
I15 (+) (B)	CN14A	Staphylococcus aureus	1.865	Staphylococcus aureus	1.839
I16 (-) (C)	CN14A	**not reliable identification**	1.624	**not reliable identification**	1.437
I17 (-) (C)	CN14B	**not reliable identification**	1.546	**not reliable identification**	1.489

I18<br>**(-) (C)**	CN14B	**not reliable identification**	1.369	**not reliable identification**	1.359
I19<br>**(-) (C)**	CN14C	**not reliable identification**	1.377	**not reliable identification**	1.353
I20<br>**(-) (C)**	CN14C	**not reliable identification**	1.517	**not reliable identification**	1.435
I21<br>**(+) (B)**	CN24A	Rothia mucilaginosa	1.773	**not reliable identification**	1.668
I22<br>**(++) (A)**	CN24A	Rothia dentocariosa	2.016	Rothia mucilaginosa	1.947
I23<br>**(-) (C)**	CN24B	**not reliable identification**	1.508	**not reliable identification**	1.489
I24<br>**(+) (B)**	CN24B	Streptococcus salivarius	1.756	**not reliable identification**	1.613
J1<br>**(+) (B)**	CN8A	Rothia dentocariosa	1.902	**not reliable identification**	1.58
J2<br>**(+) (B)**	CN8A	Rothia dentocariosa	1.736	**not reliable identification**	1.677
J3<br>**(+) (B)**	CN8B	Rothia dentocariosa	1.713	**not reliable identification**	1.514
J4<br>**(-) (C)**	CN8B	**not reliable identification**	1.492	**not reliable identification**	1.435

Matching Hints

Matched Pattern	Comment
Acinetobacter baumannii B389 UFL	Member of the Acinetobacter baumannii /calcoaceticus complex. Extraction must be performed to permit reliable species identification.
Acinetobacter calcoaceticus B388 UFL	Member of the Acinetobacter baumannii /calcoaceticus complex. Extraction must be performed to permit reliable species identification.
Acinetobacter haemolyticus LMG 1033 HAM	Species of this genus have very similar patterns: Therefore distinguishing their species is difficult.
Acinetobacter junii DSM 14968 HAM	Species of this genus have very similar patterns: Therefore distinguishing their species is difficult.
Aerococcus viridans CCM 2439 CCM	Aerococcus viridans is/are closely related to the rare species Aerococcus urinaeequi which is/are not included in the MALDI Biotyper database.
Aeromonas encheleia CECT 4342T DSM	Species of this genus have very similar patterns: Therefore distinguishing their species is difficult.
Aeromonas eucrenophila CECT 4224T DSM	Species of this genus have very similar patterns: Therefore distinguishing their species is difficult.
Aeromonas hydrophila CECT 839T DSM	Species of this genus have very similar patterns: Therefore distinguishing their species is difficult.
Aeromonas jandaei CECT 4228T DSM	Species of this genus have very similar patterns: Therefore distinguishing their species is difficult.
Aeromonas media CECT 4232T DSM	Species of this genus have very similar patterns: Therefore distinguishing their species is difficult.
Aeromonas molluscorum 848T DSM	Species of this genus have very similar patterns: Therefore distinguishing their species is difficult.
Aeromonas salmonicida ssp salmonicida CECT 894T DSM	Species of this genus have very similar patterns: Therefore distinguishing their species is difficult.
Aeromonas schubertii CECT 4240T DSM	Species of this genus have very similar patterns: Therefore distinguishing their species is difficult.
Aeromonas veronii CECT 4199T DSM	Species of this genus have very similar patterns: Therefore distinguishing their species is difficult.
Aeromonas veronii CECT 4257T DSM	Species of this genus have very similar patterns: Therefore distinguishing their species is difficult.
Aspergillus glaucus_CC7 120227_12 ETL	Isolates of DIFFERENT SPECIES are closely related and currently hard to distinguish on species level. Appropriate species of such a group are marked with the same number after the "_CC" extension. Members of _CC7 group are Aspergillus glaucus / Aspergillus_amstelodami[ana] Eurotium_amstelodami[teleo] and were marked as "Genus species_CC7" to show that these species

	belong to the same group.
Aspergillus_amstelodami[ana] Eurotium_amstelodami[teleo] _CC7 MPA 1332 MPA	Isolates of DIFFERENT SPECIES are closely related and currently hard to distinguish on species level. Appropriate species of such a group are marked with the same number after the "_CC" extension. Members of _CC7 group are Aspergillus glaucus / Aspergillus_amstelodami[ana] Eurotium_amstelodami[teleo] and were marked as "Genus species_CC7" to show that these species belong to the same group.
Bacillus aquimaris DSM 16205T DSM	The quality of spectra (score) depends on the degree of sporulation: Use fresh material.
Bacillus atrophaeus DSM 2277 DSM	is a member of Bacillus subtilis group. The quality of spectra (score) depends on the degree of sporulation: Use fresh material.
Bacillus atrophaeus DSM 5551 DSM	is a member of Bacillus subtilis group. The quality of spectra (score) depends on the degree of sporulation: Use fresh material.
Bacillus atrophaeus DSM 675 DSM	is a member of Bacillus subtilis group. The quality of spectra (score) depends on the degree of sporulation: Use fresh material.
Bacillus cereus DSM 31T DSM	Bacillus anthracis, cereus, mycoides, pseudomycoides, thuringiensis and weihenstephanensis are closely related and members of the Bacillus cereus group. In particular Bacillus cereus spectra are very similar to spectra from Bacillus anthracis. Bacillus anthracis is not included in the MALDI Biotyper database. For differentiation an adequate identification method has to be selected by an experienced professional. The quality of spectra (score) depends on the degree of sporulation: Use fresh material.
Bacillus drentensis DSM 15600T DSM	The quality of spectra (score) depends on the degree of sporulation: Use fresh material.
Bacillus farraginis DSM 16013T DSM	The quality of spectra (score) depends on the degree of sporulation: Use fresh material.
Bacillus gibsonii DSM 8722T DSM	The quality of spectra (score) depends on the degree of sporulation: Use fresh material.
Bacillus horikoshii DSM 8719T DSM	The quality of spectra (score) depends on the degree of sporulation: Use fresh material.
Bacillus litoralis DSM 16303T DSM	The quality of spectra (score) depends on the degree of sporulation: Use fresh material.
Bacillus megaterium DSM 32T DSM	The quality of spectra (score) depends on the degree of sporulation: Use fresh material.
Bacillus muralis DSM 16288T DSM	The quality of spectra (score) depends on the degree of sporulation: Use fresh material.
Bacillus pseudomycoides DSM 12442T DSM	Bacillus anthracis, cereus, mycoides, pseudomycoides, thuringiensis and weihenstephanensis are closely related and members of the Bacillus cereus group. In particular Bacillus cereus spectra are very similar to spectra from Bacillus anthracis. Bacillus anthracis is not included in the MALDI Biotyper database. For differentiation an adequate identification method has to be selected by an experienced

	professional. The quality of spectra (score) depends on the degree of sporulation: Use fresh material.
Burkholderia anthina LMG 16670 HAM	is a member of Burkholderia cepacia complex
Burkholderia cenocepacia LMG 12614 HAM	is a member of Burkholderia cepacia complex
Burkholderia cepacia LMG 2161 HAM	is a member of Burkholderia cepacia complex
Burkholderia cepacia MB_7544_05 THL	is a member of Burkholderia cepacia complex
Burkholderia cepacia_Group 18875_1 CHB	is a member of Burkholderia cepacia complex
Burkholderia pyrrocinia LMG 14191T HAM	is a member of Burkholderia cepacia complex
Burkholderia stabilis LMG 14294T HAM	is a member of Burkholderia cepacia complex
Burkholderia thailandensis DSM 13276T HAM	Burkholderia thailandensis is closely related and shows very similar spectra to the highly pathogenic Burkholderia pseudomallei / mallei which are not included in the MALDI Biotyper database. For differentiation an adequate identification method has to be selected by an experienced professional.
Citrobacter freundii 13158_2 CHB	Species of this genus have very similar patterns: Therefore distinguishing their species is difficult.
Clostridium clostridioforme 1021_NCTC 11224T BOG	Species bolteae / clostridioforme of the genus Clostridium have very similar patterns: Therefore distinguishing their species is difficult.
Clostridium haemolyticum 1069_ATCC 9650T BOG	Clostridium haemolyticum is closely related and shows very similar spectra to the strains of the highly pathogenic Clostridium botulinum groups C and D. Clostridium botulinum is not included in the MALDI Biotyper database. For differentiation an adequate identification method has to be selected by an experienced professional.
Clostridium sphenoides 1046_NCTC 507T BOG	Species celerecrescens / sphenoides of the genus Clostridium have very similar patterns: Therefore distinguishing their species is difficult.
Corynebacterium ciconiae DSM 44920T DSM	Species of this genus have very similar patterns: Therefore distinguishing their species is difficult.
Corynebacterium confusum DSM 44384T DSM	Species of this genus have very similar patterns: Therefore distinguishing their species is difficult.
Corynebacterium flavescens DSM 20296T DSM	Species of this genus have very similar patterns: Therefore distinguishing their species is difficult.
Corynebacterium flavescens IMET 11080T HKJ	Species of this genus have very similar patterns: Therefore distinguishing their species is difficult.
Corynebacterium	Species of this genus have very similar patterns: Therefore

kroppenstedtii DSM 44385T DSM	distinguishing their species is difficult.
Corynebacterium macginleyi DSM 44284T DSM	Species of this genus have very similar patterns: Therefore distinguishing their species is difficult.
Corynebacterium pilosum DSM 20521T DSM	Species of this genus have very similar patterns: Therefore distinguishing their species is difficult.
Corynebacterium pseudotuberculosis DSM 20689T DSM	Species pseudotuberculosis / ulcerans of the genus Corynebacterium have very similar patterns: Therefore distinguishing their species is difficult.
Corynebacterium thomssenii DSM 44276T DSM	Species of this genus have very similar patterns: Therefore distinguishing their species is difficult.
Corynebacterium urealyticum CCUG 17231 CCUG	Species of this genus have very similar patterns: Therefore distinguishing their species is difficult.
Corynebacterium urealyticum DSM 7110 DSM	Species of this genus have very similar patterns: Therefore distinguishing their species is difficult.
Elizabethkingia miricola DSM 14571T HAM	Species anophelis / meningoseptica / miricola of the genus Elizabethkingia have very similar patterns: Therefore distinguishing their species is difficult.
Enterobacter cloacae 13159_1 CHB	is a member of Enterobacter cloacae complex
Enterobacter cloacae DSM 3264 DSM	is a member of Enterobacter cloacae complex
Escherichia coli ATCC 25922 CHB	closely related to Shigella and not definitely distinguishable at the moment
Escherichia coli DH5alpha BRL	closely related to Shigella and not definitely distinguishable at the moment
Fusarium dimerum_BB 080809 VML	Two (or more) isolates from one species don't match (or match badly) with each other.
Fusarium proliferatum_CC3 080909 VML	Isolates of DIFFERENT SPECIES are closely related and currently hard to distinguish on species level. Appropriate species of such a group are marked with the same number after the "_CC" extension. Members of _CC3 group are Fusarium proliferatum / verticillioides / moniliforme and were marked as "Genus species_CC3" to show that these species belong to the same group.
Klebsiella oxytoca ATCC 700324 THL	Klebsiella oxytoca and species ornithinolytica / planticola / terrigena of the genus Raoultella have very similar patterns: Therefore distinguishing their species is difficult.
Lactobacillus acidophilus DSM 20079T DSM	Species acidophilus / amylovorus / gallinarum / kitasatonis of the genus Lactobacillus have very similar patterns: Therefore distinguishing their species is difficult.
Lactobacillus amylovorus DSM 20532 DSM	Species acidophilus / amylovorus / gallinarum / kitasatonis of the genus Lactobacillus have very similar patterns: Therefore distinguishing their species is difficult.

Lactobacillus crispatus DSM 20584T DSM	Species crispatus / ultunensis of the genus Lactobacillus have very similar patterns: Therefore distinguishing their species is difficult.
Mycobacterium abscessus ssp abscessus DSM 44196T DSM b	Mass spectra of Mycobacterium abscessus ssp abscessus and Mycobacterium abscessus ssp bolletii are very similar. For differentiation of both subspecies an adequate identification method has to be selected by an experienced professional.
Neisseria meningitidis 24086406 MLD	Non-pathogenic Neisseria species could be misidentified as Neisseria meningitidis. For differentiation an adequate identification method has to be selected by an experienced professional.
Neisseria meningitidis 639 PGM	Non-pathogenic Neisseria species could be misidentified as Neisseria meningitidis. For differentiation an adequate identification method has to be selected by an experienced professional.
Neisseria meningitidis C1 2 PGM	Non-pathogenic Neisseria species could be misidentified as Neisseria meningitidis. For differentiation an adequate identification method has to be selected by an experienced professional.
Neisseria meningitidis Serogroup_A BRL	Non-pathogenic Neisseria species could be misidentified as Neisseria meningitidis. For differentiation an adequate identification method has to be selected by an experienced professional.
Neisseria meningitidis Serogroup_X BRL	Non-pathogenic Neisseria species could be misidentified as Neisseria meningitidis. For differentiation an adequate identification method has to be selected by an experienced professional.
Neisseria meningitidis Serogroup_Y BRL	Non-pathogenic Neisseria species could be misidentified as Neisseria meningitidis. For differentiation an adequate identification method has to be selected by an experienced professional.
Pantoea agglomerans CCM 298 CCM	synonym of Erwinia herbicola
Pantoea agglomerans CCM 4412 CCM	synonym of Erwinia herbicola
Penicillium discolor_DD MPA 1338 MPA	Only ONE isolate represents the dedicated species in the current Filamentous Fungi DB and shows no relation to any other species. Therefore a MALDI based strain clustering to ensure the dedicated species was not possible.
Pseudomonas congelans DSM 14939T HAM	is a member of Pseudomonas fluorescens group
Pseudomonas mandelii CIP 105273T HAM	is a member of Pseudomonas fluorescens group
Pseudomonas marginalis DSM 13124T HAM	is a member of Pseudomonas fluorescens group
Pseudomonas migulae CIP 105470T HAM	is a member of Pseudomonas fluorescens group
Pseudomonas mucidolens LMG 2223T HAM	is a member of Pseudomonas fluorescens group
Pseudomonas oryzihabitans	is a member of Pseudomonas putida group

DSM 6835T HAM	
Pseudomonas putida B342T UFL	is a member of Pseudomonas putida group
Pseudomonas putida DSM 291T HAM	is a member of Pseudomonas putida group
Pseudomonas stutzeri B367 UFL	is a member of Pseudomonas stutzeri group
Pseudomonas stutzeri DSM 5190T HAM	is a member of Pseudomonas stutzeri group
Pseudomonas veronii B560 UFL	is a member of Pseudomonas fluorescens group
Psychrobacillus psychrodurans DSM 11713T DSM	The quality of spectra (score) depends on the degree of sporulation: Use fresh material.
Raoultella planticola DSM 3069T DSM	Klebsiella oxytoca and species ornithinolytica / planticola / terrigena of the genus Raoultella have very similar patterns: Therefore distinguishing their species is difficult.
Salmonella sp (enterica st Dublin) Sa05_188 VAB	Salmonella can only be identified on genus level.
Stenotrophomonas maltophilia (Pseudomonas beteli) LMG 978T HAM	is a member of Stenotrophomonas maltophilia group or closely related
Stenotrophomonas maltophilia (Pseudomonas geniculata) LMG 2195T HAM	is a member of Stenotrophomonas maltophilia group or closely related
Stenotrophomonas maltophilia 10942 CHB	is a member of Stenotrophomonas maltophilia group or closely related
Streptococcus dysgalactiae ssp equisimilis DSM 6176 DSM	Species canis / dysgalactiae / pyogenes of the genus Streptococcus have very similar patterns: Therefore distinguishing their species is difficult.
Streptococcus gallolyticus ssp gallolyticus DSM 16831T DSM	Species equinus / gallolyticus / infantarius / lutetiensis of the genus Streptococcus have very similar patterns: Therefore distinguishing their species is difficult.
Streptococcus gallolyticus ssp pasteurianus DSM 15351T DSM	Species equinus / gallolyticus / infantarius / lutetiensis of the genus Streptococcus have very similar patterns: Therefore distinguishing their species is difficult.
Streptococcus lutetiensis DSM 15350T DSM	Species equinus / gallolyticus / infantarius / lutetiensis of the genus Streptococcus have very similar patterns: Therefore distinguishing their species is difficult.
Streptococcus mitis NRZ 49039 NRZ	Streptococcus mitis / oralis / peroris / pneumoniae / pseudopneumoniae are closely related! The result may be confirmed by a further test, e.g. bile test or optochin test, according to standard clinical microbiological practice.

Streptococcus mitis NRZ 49925 NRZ	Streptococcus mitis / oralis / peroris / pneumoniae / pseudopneumoniae are closely related! The result may be confirmed by a further test, e.g. bile test or optochin test, according to standard clinical microbiological practice.
Streptococcus oralis DSM 20627T DSM	Streptococcus mitis / oralis / peroris / pneumoniae / pseudopneumoniae are closely related! The result may be confirmed by a further test, e.g. bile test or optochin test, according to standard clinical microbiological practice.
Streptococcus pneumoniae ATCC 49619 THL	Streptococcus mitis / oralis / peroris / pneumoniae / pseudopneumoniae are closely related! The result may be confirmed by a further test, e.g. bile test or optochin test, according to standard clinical microbiological practice.
Streptococcus pneumoniae besSt29 THL	Streptococcus mitis / oralis / peroris / pneumoniae / pseudopneumoniae are closely related! The result may be confirmed by a further test, e.g. bile test or optochin test, according to standard clinical microbiological practice.
Streptococcus pneumoniae DSM 20566T DSM	Streptococcus mitis / oralis / peroris / pneumoniae / pseudopneumoniae are closely related! The result may be confirmed by a further test, e.g. bile test or optochin test, according to standard clinical microbiological practice.
Streptococcus pneumoniae NRZ 28221 NRZ	Streptococcus mitis / oralis / peroris / pneumoniae / pseudopneumoniae are closely related! The result may be confirmed by a further test, e.g. bile test or optochin test, according to standard clinical microbiological practice.
Streptococcus pneumoniae NRZ 31870 NRZ	Streptococcus mitis / oralis / peroris / pneumoniae / pseudopneumoniae are closely related! The result may be confirmed by a further test, e.g. bile test or optochin test, according to standard clinical microbiological practice.
Streptococcus pneumoniae V17_201197 MUZ	Streptococcus mitis / oralis / peroris / pneumoniae / pseudopneumoniae are closely related! The result may be confirmed by a further test, e.g. bile test or optochin test, according to standard clinical microbiological practice.
Streptococcus pyogenes ATCC 19615 THL	Species canis / dysgalactiae / pyogenes of the genus Streptococcus have very similar patterns: Therefore distinguishing their species is difficult.
Trichoderma koningii_BB F59_2 LLH	Two (or more) isolates from one species don't match (or match badly) with each other.
Vibrio alginolyticus CCM 5941 CCM	is a member of Vibrio harveyi group

Meaning of Score Values

Range	Description	Symbols	Color
**2.300 ... 3.000**	**highly probable species identification**	**(+++)**	green
**2.000 ... 2.299**	**secure genus identification, probable species identification**	**(++)**	green
**1.700 ... 1.999**	**probable genus identification**	**(+)**	yellow
**0.000 ... 1.699**	**not reliable identification**	**(-)**	red

Meaning of Consistency Categories (A - C)

Category	Description
**A**	**Species Consistency:** The best match was classified as 'green' (see above). Further 'green' matches are of the same species as the first one. Further 'yellow' matches are at least of the same genus as the first one.
**B**	**Genus Consistency:** The best match was classified as 'green' or 'yellow' (see above). Further 'green' or 'yellow' matches have at least the same genus as the first one. The conditions of species consistency are not fulfilled.
**C**	**No Consistency:** Neither species nor genus consistency (Please check for synonyms of names or microbial mixture).

Analyte1

Analyte Name:	A1
Analyte Description:	
Analyte ID:	1
Analyte Creation Date/Time:	2019-12-04T13:54:31.799
Applied MSP Library(ies):	BDAL, Filamentous Fungi Library 1.0, Mycobacteria Library 1.0 (bead method), IVD, Listeria
Applied Taxonomy Tree:	

Rank (Quality)	Matched Pattern	Score Value	NCBI Identifier
1			

(-)	Lactobacillus paraplantarum DSM 10641 DSM	1.498	60520
2<br>(-)	Clostridium difficile MB_7869_05 THL	1.444	1496
3<br>(-)	Lactobacillus ruminis DSM 20511 DSM	1.442	1623
4<br>(-)	Lactobacillus gastricus DSM 16046 DSM	1.44	227942
5<br>(-)	Mycobacterium tuberculosis W148 R_722_HI PGM	1.439	1773
6<br>(-)	Staphylococcus sciuri ssp carnaticus DSM 15613T DSM	1.384	147468
7<br>(-)	Colletotrichum gloeosporioides CBS 100471 CBS	1.363	474922
8<br>(-)	Lactobacillus murinus DSM 20452T DSM	1.356	1622
9<br>(-)	Arthrobacter sulfonivorans DSM 14002T DSM	1.347	121292
10<br>(-)	Clostridium difficile MB_294_05 THL	1.341	1496

Analyte2

Analyte Name:	A2
Analyte Description:	
Analyte ID:	1
Analyte Creation Date/Time:	2019-12-04T13:54:31.941
Applied MSP Library(ies):	Listeria, IVD, Mycobacteria Library 1.0 (bead method), Filamentous Fungi Library 1.0, BDAL
Applied Taxonomy Tree:	

Rank (Quality)	Matched Pattern	Score Value	NCBI Identifier
1 (-)	Lactobacillus murinus DSM 20453 DSM	1.506	1622
2 (-)	Lactobacillus satsumensis DSM 16230T DSM	1.501	259059
3 (-)	Bifidobacterium saeculare DSM 6533 DSM	1.493	78257
4 (-)	Staphylococcus cohnii ssp cohnii DSM 20260T DSM	1.464	74704
5 (-)	Filifactor villosus 1051_NCTC 11220T BOG	1.427	29374
6 (-)	Lactobacillus paraplantarum DSM 10641 DSM	1.401	60520
7 (-)	Agromyces rhizospherae HKI 302_DSM 14597T HKJ	1.399	88374
8 (-)	Pseudomonas savastanoi ssp savastanoi LMG 5011 HAM	1.388	29438
9 (-)	Corynebacterium confusum DSM 44384T DSM	1.347	71254
10 (-)	Mycobacterium bovis BCG 1878 PGM	1.344	1765

Analyte3

Analyte Name:	A3
Analyte Description:	
Analyte ID:	2
Analyte Creation Date/Time:	2019-12-04T13:54:32.412
Applied MSP Library(ies):	BDAL, Filamentous Fungi Library 1.0, Mycobacteria Library 1.0 (bead method), IVD, Listeria
Applied Taxonomy Tree:	

Rank (Quality)	Matched Pattern	Score Value	NCBI Identifier
1 (-)	Lactobacillus agilis DSM 20510 DSM	1.422	1601
2 (-)	Arthrobacter crystallopoietes DSM 20117T DSM	1.391	37928
3 (-)	Staphylococcus hominis 18 ESL	1.336	1290
4 (-)	Agromyces salentinus HKI 320_DSM 16198T HKJ	1.33	269421
5 (-)	Moraxella_sg_Moraxella lincolnii DSM 19150T DSM	1.323	90241
6 (-)	Cryptococcus neoformans 29 PSB	1.323	5207
7 (-)	Actinocorallia libanotica B246 UFL	1.305	46162
8 (-)	Staphylococcus vitulinus DSM 15615T DSM	1.293	71237
9 (-)	Candida guilliermondii CBS 566 CBS	1.278	4929
10 (-)	Candida lambica CBS 603 CBS	1.277	53655

Analyte4

Analyte Name:	A4
Analyte Description:	
Analyte ID:	2
Analyte Creation Date/Time:	2019-12-04T13:54:32.630
Applied MSP Library(ies):	BDAL, Filamentous Fungi Library 1.0, Mycobacteria Library 1.0 (bead method), IVD, Listeria
Applied Taxonomy Tree:	

Rank (Quality)	Matched Pattern	Score Value	NCBI Identifier
1 (-)	Lactobacillus fermentum DSM 20391 DSM	1.376	1613
2 (-)	Staphylococcus epidermidis 6b_s ESL	1.375	1282
3 (-)	Lactobacillus satsumensis DSM 16230T DSM	1.36	259059
4 (-)	Aromatoleum aromaticum EbN1 MPB	1.348	12960
5 (-)	Myroides odoratus DSM 2801T HAM	1.344	256
6 (-)	Thauera terpenica 58Eu MPB	1.325	76113
7 (-)	Mycobacterium tuberculosis AI60 R_434 PGM	1.299	1773
8 (-)	Corynebacterium accolens 87_D5_coll ISB	1.299	38284
9 (-)	Staphylococcus aureus ssp aureus DSM 4910 DSM	1.292	46170
10 (-)	Lactobacillus murinus DSM 20453 DSM	1.287	1622

Analyte5

Analyte Name:	A5
Analyte Description:	
Analyte ID:	3
Analyte Creation Date/Time:	2019-12-04T13:54:31.846
Applied MSP Library(ies):	BDAL, Filamentous Fungi Library 1.0, Mycobacteria Library 1.0 (bead method), IVD, Listeria
Applied Taxonomy Tree:	

Rank (Quality)	Matched Pattern	Score Value	NCBI Identifier
1 (-)	Lactobacillus murinus DSM 20453 DSM	1.428	1622
2 (-)	Streptomyces lavendulae B264 UFL	1.414	1914
3 (-)	Streptococcus salivarius DSM 20067 DSM	1.403	1304
4 (-)	Streptomyces phaeochromogenes B265 UFL	1.401	1923
5 (-)	Nocardia cyriacigeorgica DSM 44484T DSM	1.389	135487
6 (-)	Serratia rubidaea CCM 4684 CCM	1.359	61652
7 (-)	Mycobacterium bovis BCG 1878 PGM	1.352	1765
8 (-)	Arthrobacter nicotinovorans DSM 420T DSM	1.35	29320
9 (-)	Aromatoleum anaerobicus LuFRes1 MPB	1.342	182180
10 (-)	Cryptococcus neoformans RV07_02 18 VML	1.342	5207

Analyte6

Analyte Name:	A6
Analyte Description:	
Analyte ID:	3
Analyte Creation Date/Time:	2019-12-04T13:54:32.695
Applied MSP Library(ies):	Listeria, IVD, Mycobacteria Library 1.0 (bead method), Filamentous Fungi Library 1.0, BDAL
Applied Taxonomy Tree:	

Rank (Quality)	Matched Pattern	Score Value	NCBI Identifier
1 (-)	Pseudomonas pertucinogena LMG 1874T HAM	1.438	86175
2 (-)	Agromyces rhizospherae HKI 302_DSM 14597T HKJ	1.427	88374
3 (-)	Pseudomonas viridiflava DSM 11124T HAM	1.414	33069
4 (-)	Aromatoleum toluvorans Td21 MPB	1.384	92002
5 (-)	Pseudomonas syringae ssp syringae LMG 1247T HAM	1.337	317
6 (-)	Arthrobacter oxydans DSM 20119T DSM	1.324	1671
7 (-)	Aeromonas media CECT 4232T DSM	1.314	651
8 (-)	Mycobacterium bovis BCG 1878 PGM	1.313	1765
9 (-)	Aromatoleum anaerobicus LuFRes1 MPB	1.308	182180
10 (-)	Aeromonas encheleia CECT 4342T DSM	1.303	73010

Analyte7

Analyte Name:	A7
Analyte Description:	
Analyte ID:	4
Analyte Creation Date/Time:	2019-12-04T13:54:32.469
Applied MSP Library(ies):	BDAL, Filamentous Fungi Library 1.0, Mycobacteria Library 1.0 (bead method), IVD, Listeria
Applied Taxonomy Tree:	

Rank (Quality)	Matched Pattern	Score Value	NCBI Identifier
1 (-)	Clostridium novyi 1082_ATCC 17861T BOG	1.449	1542
2 (-)	Mycobacterium tuberculosis W336 R_880 PGM	1.389	1773
3 (-)	Nocardia cyriacigeorgica DSM 44484T DSM	1.373	135487
4 (-)	Lactobacillus satsumensis DSM 16230T DSM	1.345	259059
5 (-)	Mycobacterium tuberculosis W148 R_722_HI PGM	1.338	1773
6 (-)	Lactobacillus agilis DSM 20509T DSM	1.314	1601
7 (-)	Agromyces salentinus HKI 320_DSM 16198T HKJ	1.313	269421
8 (-)	Lactobacillus ruminis DSM 20404 DSM	1.309	1623
9 (-)	Thauera terpenica 58Eu MPB	1.307	76113
10 (-)	Lactobacillus murinus DSM 20453 DSM	1.305	1622

Analyte8

Analyte Name:	A8
Analyte Description:	
Analyte ID:	4
Analyte Creation Date/Time:	2019-12-04T13:54:32.019
Applied MSP Library(ies):	BDAL, Filamentous Fungi Library 1.0, Mycobacteria Library 1.0 (bead method), IVD, Listeria
Applied Taxonomy Tree:	

Rank (Quality)	Matched Pattern	Score Value	NCBI Identifier
1 (-)	Agromyces rhizospherae HKI 302_DSM 14597T HKJ	1.517	88374
2 (-)	Filifactor villosus 1051_NCTC 11220T BOG	1.455	29374
3 (-)	Candida guilliermondii CBS 566 CBS	1.441	4929
4 (-)	Candida lambica CBS 603 CBS	1.433	53655
5 (-)	Alishewanella fetalis DSM 16032T HAM	1.348	111143
6 (-)	Nocardia ignorata DSM 44496T DSM	1.345	145285
7 (-)	Lactobacillus antri DSM 16041T DSM	1.344	227943
8 (-)	Agromyces salentinus HKI 320_DSM 16198T HKJ	1.342	269421
9 (-)	Aeromonas media CECT 4232T DSM	1.341	651
10 (-)	Pichia occidentalis CBS 1910 CBS	1.34	54552

Analyte9

Analyte Name:	A9
Analyte Description:	
Analyte ID:	5
Analyte Creation Date/Time:	2019-12-04T13:54:32.502
Applied MSP Library(ies):	BDAL, Filamentous Fungi Library 1.0, Mycobacteria Library 1.0 (bead method), IVD, Listeria
Applied Taxonomy Tree:	

Rank (Quality)	Matched Pattern	Score Value	NCBI Identifier
1 (-)	Lactobacillus plantarum DSM 13273 DSM	1.583	1590
2 (-)	Lactobacillus plantarum DSM 2601 DSM	1.575	1590
3 (-)	Lactobacillus plantarum DSM 12028 DSM	1.571	1590
4 (-)	Lactobacillus plantarum ssp argentoratensis DSM 16365T DSM	1.558	271881
5 (-)	Lactobacillus plantarum DSM 20246 DSM	1.541	1590
6 (-)	Lactobacillus plantarum DSM 2648 DSM	1.537	1590
7 (-)	Lactobacillus plantarum ssp plantarum DSM 20174T DSM	1.517	337330
8 (-)	Hydrogenophaga flava B339 UFL	1.419	65657
9 (-)	Escherichia coli ATCC 25922 CHB	1.405	562
10 (-)	Lactobacillus plantarum DSM 1055 DSM	1.389	1590

Analyte10

Analyte Name:	A10
Analyte Description:	
Analyte ID:	5
Analyte Creation Date/Time:	2019-12-04T13:54:32.545
Applied MSP Library(ies):	BDAL, Filamentous Fungi Library 1.0, Mycobacteria Library 1.0 (bead method), IVD, Listeria
Applied Taxonomy Tree:	

Rank (Quality)	Matched Pattern	Score Value	NCBI Identifier
1 (-)	Lactobacillus plantarum DSM 20205 DSM	1.641	1590
2 (-)	Lactobacillus plantarum ssp plantarum DSM 20174T DSM	1.621	337330
3 (-)	Lactobacillus plantarum DSM 2601 DSM	1.618	1590
4 (-)	Lactobacillus plantarum DSM 13273 DSM	1.611	1590
5 (-)	Lactobacillus plantarum DSM 20246 DSM	1.492	1590
6 (-)	Lactobacillus plantarum DSM 12028 DSM	1.446	1590
7 (-)	Lactobacillus plantarum DSM 2648 DSM	1.441	1590
8 (-)	Lactobacillus gasseri DSM 20077 DSM	1.33	1596
9 (-)	Lactobacillus plantarum DSM 1055 DSM	1.328	1590
10 (-)	Lactobacillus plantarum ssp argentoratensis DSM 16365T DSM	1.328	271881

Analyte11

Analyte Name:	A11
Analyte Description:	
Analyte ID:	6
Analyte Creation Date/Time:	2019-12-04T13:54:32.554
Applied MSP Library(ies):	Filamentous Fungi Library 1.0, Mycobacteria Library 1.0 (bead method), IVD, Listeria, BDAL
Applied Taxonomy Tree:	

Rank (Quality)	Matched Pattern	Score Value	NCBI Identifier
1 (-)	Clostridium bifermentans 2273_CCUG 35297 BOG	1.423	1490
2 (-)	Pseudomonas stutzeri B367 UFL	1.383	316
3 (-)	Pseudomonas stutzeri DSM 5190T HAM	1.382	316
4 (-)	Photobacterium iliopiscarium DSM 9896T HAM	1.364	56192
5 (-)	Rhizobium radiobacter B167 UFL	1.358	358
6 (-)	Lactobacillus curvatus DSM 20495 DSM	1.317	28038
7 (-)	Clostridium bifermentans 1027_NCTC 1341 BOG	1.302	1490
8 (-)	Aeromonas veronii CECT 4199T DSM	1.297	654
9 (-)	Rhizobium radiobacter B178 UFL	1.29	358
10 (-)	Aeromonas veronii CECT 4257T DSM	1.276	654

Analyte12

Analyte Name:	A12
Analyte Description:	
Analyte ID:	6
Analyte Creation Date/Time:	2019-12-04T13:54:32.589
Applied MSP Library(ies):	BDAL, Filamentous Fungi Library 1.0, Mycobacteria Library 1.0 (bead method), IVD, Listeria
Applied Taxonomy Tree:	

Rank (Quality)	Matched Pattern	Score Value	NCBI Identifier
1 (-)	Corynebacterium pseudotuberculosis DSM 20689T DSM	1.409	1719
2 (-)	Clostridium bifermentans 1027_NCTC 1341 BOG	1.37	1490
3 (-)	Staphylococcus simulans DSM 20324 DSM	1.368	1286
4 (-)	Clostridium difficile 1020_NCTC 11206 BOG	1.348	1496
5 (-)	Cryptococcus neoformans ATCC 14116 THL	1.346	5207
6 (-)	Staphylococcus condimenti DSM 11674T DSM	1.34	70255
7 (-)	Pseudomonas putida B342T UFL	1.338	303
8 (-)	Pseudomonas thermotolerans DSM 14292T HAM	1.336	157784
9 (-)	Rhizobium radiobacter B167 UFL	1.314	358
10 (-)	Pasteurella multocida FI FLR	1.313	747

Analyte13

Analyte Name: A13
Analyte Description:
Analyte ID: 7
Analyte Creation Date/Time: 2019-12-04T13:54:31.756
Applied MSP Library(ies): BDAL, Filamentous Fungi Library 1.0, Mycobacteria Library 1.0 (bead method), IVD, Listeria
Applied Taxonomy Tree:

Rank (Quality)	Matched Pattern	Score Value	NCBI Identifier
1 (-)	Clostridium bifermentans 2274_CCUG 35556 A BOG	1.382	1490
2 (-)	Myroides odoratus DSM 2801T HAM	1.374	256
3 (-)	Clostridium histolyticum 1036_NCTC 503T BOG	1.366	1498
4 (-)	Stenotrophomonas maltophilia (Pseudomonas geniculata) LMG 2195T HAM	1.302	40324
5 (-)	Pasteurella multocida FI FLR	1.299	747
6 (-)	Klebsiella pneumoniae ssp pneumoniae 9295_1 CHB	1.294	72407
7 (-)	Stenotrophomonas maltophilia (Pseudomonas beteli) LMG 978T HAM	1.294	40324
8 (-)	Filifactor villosus 1051_NCTC 11220T BOG	1.286	29374
9 (-)	Actinocorallia libanotica B246 UFL	1.267	46162
10 (-)	Trichosporon debeurmannianum VML	1.266	129131

Analyte14

Analyte Name:	A14
Analyte Description:	
Analyte ID:	7
Analyte Creation Date/Time:	2019-12-04T13:54:32.297
Applied MSP Library(ies):	BDAL, Filamentous Fungi Library 1.0, Mycobacteria Library 1.0 (bead method), IVD, Listeria
Applied Taxonomy Tree:	

Rank (Quality)	Matched Pattern	Score Value	NCBI Identifier
1 (-)	Filifactor villosus 1051_NCTC 11220T BOG	1.409	29374
2 (-)	Pichia occidentalis CBS 1910 CBS	1.358	54552
3 (-)	Paenibacillus kobensis DSM 10250 DSM	1.279	59841
4 (-)	Arthrobacter nicotinovorans DSM 420T DSM	1.263	29320
5 (-)	Clostridium bifermentans 1027_NCTC 1341 BOG	1.248	1490
6 (-)	Aerococcus viridans CCM 2439 CCM	1.244	1377
7 (-)	Staphylococcus simulans DSM 20324 DSM	1.24	1286
8 (-)	Rhizobium radiobacter B177 UFL	1.23	358
9 (-)	Penicillium roqueforti DSM 1812 DSM	1.23	123269315
10 (-)	Lactobacillus satsumensis DSM 16230T DSM	1.224	259059

Analyte15

Analyte Name:	A15
Analyte Description:	
Analyte ID:	8
Analyte Creation Date/Time:	2019-12-04T13:54:32.319
Applied MSP Library(ies):	BDAL, Filamentous Fungi Library 1.0, Mycobacteria Library 1.0 (bead method), IVD, Listeria
Applied Taxonomy Tree:	

Rank (Quality)	Matched Pattern	Score Value	NCBI Identifier
1 (-)	Staphylococcus simulans DSM 20324 DSM	1.434	1286
2 (-)	Clostridium histolyticum 1036_NCTC 503T BOG	1.366	1498
3 (-)	Lactobacillus saerimneri DSM 16027 DSM	1.35	228229
4 (-)	Myroides odoratus DSM 2801T HAM	1.292	256
5 (-)	Streptomyces albus B262 UFL	1.28	1888
6 (-)	Kingella kingae CCM 5679T CCM	1.278	504
7 (-)	Paenibacillus kobensis DSM 10250 DSM	1.264	59841
8 (-)	Tsukamurella sp RV_Feb_05 MLD	1.261	2060
9 (-)	Filifactor villosus 1051_NCTC 11220T BOG	1.253	29374
10 (-)	Kingella kingae CCM 6198 CCM	1.249	504

Analyte16

Analyte Name:	A16
Analyte Description:	
Analyte ID:	8
Analyte Creation Date/Time:	2019-12-04T13:54:32.485
Applied MSP Library(ies):	Listeria, BDAL, Filamentous Fungi Library 1.0, Mycobacteria Library 1.0 (bead method), IVD
Applied Taxonomy Tree:	

Rank (Quality)	Matched Pattern	Score Value	NCBI Identifier
1 (-)	Herbaspirillum rubrisubalbicans DSM 9440T DSM	1.439	80842
2 (-)	Lactobacillus plantarum DSM 13273 DSM	1.35	1590
3 (-)	Candida parapsilosis ATCC 22019 THL	1.339	5480
4 (-)	Myroides odoratus DSM 2801T HAM	1.331	256
5 (-)	Staphylococcus simulans DSM 20324 DSM	1.329	1286
6 (-)	Aspergillus_amstelodami[ana] Eurotium_amstelodami[teleo]_CC7 MPA 1332 MPA	1.312	123269315
7 (-)	Arthrobacter nasiphocae DSM 13988T DSM	1.299	189863
8 (-)	Corynebacterium accolens 87_D5_coll ISB	1.287	38284
9 (-)	Sphingobacterium thalpophilum DSM 11723T HAM	1.287	259
10 (-)	Pseudomonas grimontii CIP 106645T HAM	1.257	129847

Analyte17

Analyte Name:	A17
Analyte Description:	
Analyte ID:	9
Analyte Creation Date/Time:	2019-12-04T13:54:32.004
Applied MSP Library(ies):	BDAL, Filamentous Fungi Library 1.0, Mycobacteria Library 1.0 (bead method), IVD, Listeria
Applied Taxonomy Tree:	

Rank (Quality)	Matched Pattern	Score Value	NCBI Identifier
1 (-)	Rhizobium radiobacter B167 UFL	1.403	358
2 (-)	Lactobacillus plantarum DSM 13273 DSM	1.333	1590
3 (-)	Streptomyces albus B262 UFL	1.275	1888
4 (-)	Clostridium bifermentans 1027_NCTC 1341 BOG	1.265	1490
5 (-)	Clostridium histolyticum 1036_NCTC 503T BOG	1.262	1498
6 (-)	Paracoccus versutus B352 UFL	1.252	34007
7 (-)	Corynebacterium macginleyi DSM 44284T DSM	1.248	38290
8 (-)	Lactobacillus sakei DSM 6333 DSM	1.244	1599
9 (-)	Pseudomonas stutzeri B367 UFL	1.242	316
10 (-)	Lactobacillus satsumensis DSM 16230T DSM	1.24	259059

Analyte18

Analyte Name:	A18
Analyte Description:	
Analyte ID:	9
Analyte Creation Date/Time:	2019-12-04T13:54:31.875
Applied MSP Library(ies):	BDAL, Filamentous Fungi Library 1.0, Mycobacteria Library 1.0 (bead method), IVD, Listeria
Applied Taxonomy Tree:	

Rank (Quality)	Matched Pattern	Score Value	NCBI Identifier
1 (-)	Clostridium bifermentans 2273_CCUG 35297 BOG	1.402	1490
2 (-)	Gordonia bronchialis DSM 43247T DSM	1.377	2054
3 (-)	Clostridium bifermentans 1027_NCTC 1341 BOG	1.359	1490
4 (-)	Candida krusei DSM 11956 DSM	1.319	4909
5 (-)	Herbaspirillum rubrisubalbicans DSM 9440T DSM	1.287	80842
6 (-)	Arthrobacter luteolus DSM 13067T DSM	1.274	98672
7 (-)	Udeniomyces puniceus DSM 4657 DSM	1.26	42660
8 (-)	Rhizobium radiobacter B336 UFL	1.226	358
9 (-)	Mycobacterium kansasii 0219 BSI	1.22	1768
10 (-)	Rhizobium radiobacter B167 UFL	1.214	358

Analyte19

Analyte Name:	A19
Analyte Description:	
Analyte ID:	10
Analyte Creation Date/Time:	2019-12-04T13:54:31.968
Applied MSP Library(ies):	Listeria, BDAL, Filamentous Fungi Library 1.0, Mycobacteria Library 1.0 (bead method), IVD
Applied Taxonomy Tree:	

Rank (Quality)	Matched Pattern	Score Value	NCBI Identifier
1 (-)	Arthrobacter histidinolovorans DSM 20115T DSM	1.394	43664
2 (-)	Enterococcus faecium 20218_1 CHB	1.366	1352
3 (-)	Candida krusei DSM 11956 DSM	1.295	4909
4 (-)	Weissella minor DSM 20014T DSM	1.232	1620
5 (-)	Lactobacillus saerimneri DSM 16027 DSM	1.221	228229
6 (-)	Staphylococcus simulans DSM 20324 DSM	1.213	1286
7 (-)	Rhizobium radiobacter B167 UFL	1.206	358
8 (-)	Arthrobacter sulfonivorans DSM 14002T DSM	1.206	121292
9 (-)	Tsukamurella paurometabola DSM 46042 DSM	1.194	2061
10 (-)	Myroides odoratus DSM 2801T HAM	1.188	256

Analyte20

Analyte Name:	A20
Analyte Description:	
Analyte ID:	10
Analyte Creation Date/Time:	2019-12-04T13:54:31.964
Applied MSP Library(ies):	BDAL, Filamentous Fungi Library 1.0, Mycobacteria Library 1.0 (bead method), IVD, Listeria
Applied Taxonomy Tree:	

Rank (Quality)	Matched Pattern	Score Value	NCBI Identifier
1 (-)	Enterococcus faecium 20218_1 CHB	1.437	1352
2 (-)	Clostridium bifermentans 2274_CCUG 35556 A BOG	1.384	1490
3 (-)	Mycobacterium tuberculosis W336 R_880 PGM	1.341	1773
4 (-)	Bergeyella zoohelcum LMG 8351T HAM	1.33	1015
5 (-)	Rhizobium radiobacter B167 UFL	1.302	358
6 (-)	Arthrobacter histidinolovorans DSM 20115T DSM	1.298	43664
7 (-)	Pseudomonas stutzeri B367 UFL	1.292	316
8 (-)	Candida krusei CBS 2107 CBS	1.272	4909
9 (-)	Staphylococcus simulans DSM 20324 DSM	1.266	1286
10 (-)	Aerococcus viridans CCM 2439 CCM	1.26	1377

Analyte21

Analyte Name:	A21
Analyte Description:	
Analyte ID:	11
Analyte Creation Date/Time:	2019-12-04T13:54:32.292
Applied MSP Library(ies):	BDAL, Filamentous Fungi Library 1.0, Mycobacteria Library 1.0 (bead method), IVD, Listeria
Applied Taxonomy Tree:	

Rank (Quality)	Matched Pattern	Score Value	NCBI Identifier
1 (-)	Candida krusei DSM 11956 DSM	1.527	4909
2 (-)	Lactobacillus paracasei ssp tolerans DSM 20012 DSM	1.338	113557
3 (-)	Aspergillus glaucus_CC7 120227_12 ETL	1.331	123269315
4 (-)	Herbaspirillum rubrisubalbicans DSM 9440T DSM	1.326	80842
5 (-)	Lactobacillus plantarum ssp argentoratensis DSM 16365T DSM	1.266	271881
6 (-)	Lactobacillus plantarum DSM 13273 DSM	1.221	1590
7 (-)	Arthrobacter crystallopoietes DSM 20117T DSM	1.209	37928
8 (-)	Gordonia aichiensis DSM 43978T DSM	1.205	36820
9 (-)	Sporopachydermia cereana 82 PIM	1.201	54093
10 (-)	Arthrobacter roseus DSM 14508T DSM	1.198	136274

Analyte22

Analyte Name:	A22
Analyte Description:	
Analyte ID:	11
Analyte Creation Date/Time:	2019-12-04T13:54:31.954
Applied MSP Library(ies):	Listeria, IVD, Mycobacteria Library 1.0 (bead method), Filamentous Fungi Library 1.0, BDAL
Applied Taxonomy Tree:	

Rank (Quality)	Matched Pattern	Score Value	NCBI Identifier
1 (-)	Streptomyces albus B262 UFL	1.462	1888
2 (-)	Myroides odoratus DSM 2801T HAM	1.398	256
3 (-)	Herbaspirillum rubrisubalbicans DSM 9440T DSM	1.395	80842
4 (-)	Candida krusei DSM 11956 DSM	1.361	4909
5 (-)	Aromatoleum bremensis PbN1 MPB	1.331	12960
6 (-)	Lactobacillus plantarum DSM 13273 DSM	1.3	1590
7 (-)	Corynebacterium pseudotuberculosis DSM 20689T DSM	1.293	1719
8 (-)	Azoarcus indigens VB32 MPB	1.291	29545
9 (-)	Cryptococcus neoformans 29 PSB	1.29	5207
10 (-)	Filifactor villosus 1051_NCTC 11220T BOG	1.272	29374

Analyte23

Analyte Name:	A23
Analyte Description:	
Analyte ID:	1A
Analyte Creation Date/Time:	2019-12-04T13:54:32.188
Applied MSP Library(ies):	BDAL, Filamentous Fungi Library 1.0, Mycobacteria Library 1.0 (bead method), IVD, Listeria
Applied Taxonomy Tree:	

Rank (Quality)	Matched Pattern	Score Value	NCBI Identifier
1 (++)	Rothia mucilaginosa DSM 20445 DSM	2.089	43675
2 (++)	Rothia mucilaginosa DSM 20746T DSM	2.084	43675
3 (++)	Rothia mucilaginosa CCUG 31189 CCUG	2.022	43675
4 (++)	Rothia mucilaginosa CCUG 44966 CCUG	2.015	43675
5 (+)	Rothia dentocariosa CCUG 29965 CCUG	1.702	2047
6 (-)	Rothia mucilaginosa BK2995_09 ERL	1.646	43675
7 (-)	Rothia mucilaginosa DSM 30548 DSM	1.641	43675
8 (-)	Rothia mucilaginosa DSM 20446 BRB	1.595	43675
9 (-)	Rothia mucilaginosa CCUG 52532 CCUG	1.49	43675
10 (-)	Paenibacillus lactis DSM 15596T DSM	1.315	228574

Analyte24

Analyte Name:	A24
Analyte Description:	
Analyte ID:	1A
Analyte Creation Date/Time:	2019-12-04T13:54:31.795
Applied MSP Library(ies):	BDAL, Filamentous Fungi Library 1.0, Mycobacteria Library 1.0 (bead method), IVD, Listeria
Applied Taxonomy Tree:	

Rank (Quality)	Matched Pattern	Score Value	NCBI Identifier
1 (+)	Rothia mucilaginosa CCUG 31189 CCUG	1.922	43675
2 (+)	Rothia mucilaginosa CCUG 44966 CCUG	1.802	43675
3 (-)	Rothia mucilaginosa DSM 30548 DSM	1.694	43675
4 (-)	Rothia dentocariosa CCUG 29965 CCUG	1.553	2047
5 (-)	Rothia mucilaginosa DSM 20446 BRB	1.498	43675
6 (-)	Rothia dentocariosa DSM 43762T DSM	1.447	2047
7 (-)	Paenibacillus lactis DSM 15596T DSM	1.405	228574
8 (-)	Arthrobacter stackebrandtii DSM 16005T DSM	1.364	272161
9 (-)	Paenibacillus glucanolyticus DSM 5162T DSM	1.363	59843
10 (-)	Paenibacillus taiwanensis DSM 18679T DSM	1.351	401638

Analyte25

Analyte Name:	B1
Analyte Description:	
Analyte ID:	1B
Analyte Creation Date/Time:	2019-12-04T13:54:31.607
Applied MSP Library(ies):	BDAL, Filamentous Fungi Library 1.0, Mycobacteria Library 1.0 (bead method), IVD, Listeria
Applied Taxonomy Tree:	

Rank (Quality)	Matched Pattern	Score Value	NCBI Identifier
1 (-)	Streptococcus sobrinus DSM 20742T DSM	1.52	1310
2 (-)	Streptococcus salivarius 0807M25049501 IBS	1.447	1304
3 (-)	Pseudomonas aeruginosa DSM 50071T HAM	1.389	287
4 (-)	Rhodococcus erythropolis DSM 43933 DSM	1.37	1833
5 (-)	Clostridium cadaveris 1074_ATCC 25783T BOG	1.369	1529
6 (-)	Pseudomonas syringae ssp syringae LMG 1247T HAM	1.359	317
7 (-)	Pseudomonas congelans DSM 14939T HAM	1.338	200452
8 (-)	Pseudomonas savastanoi ssp savastanoi LMG 5011 HAM	1.284	29438
9 (-)	Pseudomonas syringae ssp syringae DSM 6693 HAM	1.278	317
10 (-)	Lactobacillus sharpeae DSM 20504 DSM	1.225	1626

Analyte26

Analyte Name:	B2
Analyte Description:	
Analyte ID:	1B
Analyte Creation Date/Time:	2019-12-04T13:54:32.529
Applied MSP Library(ies):	BDAL, Filamentous Fungi Library 1.0, Mycobacteria Library 1.0 (bead method), IVD, Listeria
Applied Taxonomy Tree:	

Rank (Quality)	Matched Pattern	Score Value	NCBI Identifier
1 (-)	Streptococcus sobrinus DSM 20742T DSM	1.501	1310
2 (-)	Streptococcus salivarius 0807M25049501 IBS	1.375	1304
3 (-)	Staphylococcus xylosus DSM 6179 DSM	1.373	1288
4 (-)	Streptococcus vestibularis CCUG 51352 CCUG	1.338	1343
5 (-)	Bacillus drentensis DSM 15600T DSM	1.328	220684
6 (-)	Pseudomonas congelans DSM 14939T HAM	1.328	200452
7 (-)	Pseudomonas putida DSM 291T HAM	1.316	303
8 (-)	Pseudomonas aeruginosa DSM 50071T HAM	1.309	287
9 (-)	Staphylococcus saprophyticus ssp bovis DSM 18669T DSM	1.289	29385
10 (-)	Rhizobium radiobacter LMG 142 LMG	1.285	358

Analyte27

Analyte Name:	B3
Analyte Description:	
Analyte ID:	2A
Analyte Creation Date/Time:	2019-12-04T13:54:32.163
Applied MSP Library(ies):	BDAL, Filamentous Fungi Library 1.0, Mycobacteria Library 1.0 (bead method), IVD, Listeria
Applied Taxonomy Tree:	

Rank (Quality)	Matched Pattern	Score Value	NCBI Identifier
1 (-)	Weissella viridescens DSM 20248 DSM	1.432	1629
2 (-)	Paenibacillus xylanilyticus DSM 17255T DSM	1.404	248903
3 (-)	Arthrobacter stackebrandtii DSM 16005T DSM	1.341	272161
4 (-)	Bacillus atrophaeus DSM 2277 DSM	1.302	1452
5 (-)	Balneatrix alpica CIP 103589T HAM	1.3	75684
6 (-)	Paenibacillus alvei DSM 5557 DSM	1.295	44250
7 (-)	Bacillus atrophaeus DSM 5551 DSM	1.295	1452
8 (-)	Paenibacillus sp DSM 1352 DSM	1.281	44249
9 (-)	Thauera aminoaromatica S2 MPB	1.274	164330
10 (-)	Paenibacillus phyllosphaerae DSM 17399T DSM	1.252	274593

Analyte28

Analyte Name:	B4
Analyte Description:	
Analyte ID:	2A
Analyte Creation Date/Time:	2019-12-04T13:54:32.198
Applied MSP Library(ies):	BDAL, Filamentous Fungi Library 1.0, Mycobacteria Library 1.0 (bead method), IVD, Listeria
Applied Taxonomy Tree:	

Rank (Quality)	Matched Pattern	Score Value	NCBI Identifier
1 (-)	Rothia dentocariosa DSM 43762T DSM	1.697	2047
2 (-)	Rothia dentocariosa CCUG 29965 CCUG	1.528	2047
3 (-)	Rothia mucilaginosa DSM 20445 DSM	1.382	43675
4 (-)	Paenibacillus agarexedens DSM 1327T DSM	1.373	171401
5 (-)	Paenibacillus xinjiangensis DSM 16970T DSM	1.34	459527
6 (-)	Paenibacillus agarexedens DSM 1479 DSM	1.325	171401
7 (-)	Paenibacillus agarexedens DSM 1478 DSM	1.282	171401
8 (-)	Arthrobacter stackebrandtii DSM 16005T DSM	1.281	272161
9 (-)	Paenibacillus alvei DSM 5557 DSM	1.273	44250
10 (-)	Arthrobacter ureafaciens DSM 20126T DSM	1.257	37931

Analyte29

Analyte Name:	B5
Analyte Description:	
Analyte ID:	2B
Analyte Creation Date/Time:	2019-12-04T13:54:31.535
Applied MSP Library(ies):	BDAL, Filamentous Fungi Library 1.0, Mycobacteria Library 1.0 (bead method), IVD, Listeria
Applied Taxonomy Tree:	

Rank (Quality)	Matched Pattern	Score Value	NCBI Identifier
1 (+)	Streptococcus vestibularis CCUG 61229 CCUG	1.788	1343
2 (+)	Streptococcus vestibularis CCUG 51352 CCUG	1.74	1343
3 (-)	Streptococcus downei DSM 5635T DSM	1.643	1317
4 (-)	Streptococcus sobrinus DSM 20742T DSM	1.616	1310
5 (-)	Streptococcus salivarius_ssp_thermophilus DSM 8713 DSM	1.391	1304
6 (-)	Streptococcus macacae DSM 20724T DSM	1.354	1339
7 (-)	Streptococcus salivarius_ssp_thermophilus 39 RLT	1.354	1304
8 (-)	Streptococcus salivarius 0807M25049501 IBS	1.342	1304
9 (-)	Streptococcus vestibularis 14147704_2 MVD	1.333	1343
10 (-)	Streptococcus hyovaginalis DSM 12220 DSM	1.329	149015

Analyte30

Analyte Name:	B6
Analyte Description:	
Analyte ID:	2B
Analyte Creation Date/Time:	2019-12-04T13:54:32.441
Applied MSP Library(ies):	Listeria, BDAL, Filamentous Fungi Library 1.0, Mycobacteria Library 1.0 (bead method), IVD
Applied Taxonomy Tree:	

Rank (Quality)	Matched Pattern	Score Value	NCBI Identifier
1 (-)	Lactobacillus aviarius ssp aviarius DSM 20654 DSM	1.551	147810
2 (-)	Streptococcus sobrinus DSM 20742T DSM	1.476	1310
3 (-)	Streptococcus salivarius 0807M25049501 IBS	1.466	1304
4 (-)	Lactobacillus sharpeae DSM 20506 DSM	1.407	1626
5 (-)	Lactobacillus sharpeae DSM 20504 DSM	1.406	1626
6 (-)	Streptococcus salivarius_ssp_thermophilus 38 RLT	1.34	1304
7 (-)	Streptococcus vestibularis 1C15011954_3 MVD	1.335	1343
8 (-)	Streptococcus orisratti DSM 15617T DSM	1.335	114652
9 (-)	Lactobacillus sharpeae DSM 20505T DSM	1.33	1626
10 (-)	Streptococcus ferus DSM 20646T DSM	1.325	1345

Analyte31

Analyte Name:	B7
Analyte Description:	
Analyte ID:	3A
Analyte Creation Date/Time:	2019-12-04T13:54:32.422
Applied MSP Library(ies):	BDAL, Filamentous Fungi Library 1.0, Mycobacteria Library 1.0 (bead method), IVD, Listeria
Applied Taxonomy Tree:	

Rank (Quality)	Matched Pattern	Score Value	NCBI Identifier
1 (++)	Rothia mucilaginosa BK2995_09 ERL	2.032	43675
2 (+)	Rothia mucilaginosa DSM 20445 DSM	1.942	43675
3 (+)	Rothia mucilaginosa DSM 20746T DSM	1.892	43675
4 (-)	Rothia dentocariosa CCUG 29965 CCUG	1.563	2047
5 (-)	Rothia mucilaginosa DSM 30548 DSM	1.544	43675
6 (-)	Rothia mucilaginosa CCUG 52532 CCUG	1.47	43675
7 (-)	Rothia dentocariosa RV_BA1_032010_D LBK	1.428	2047
8 (-)	Sinomonas atrocyanea DSM 20127T DSM	1.358	37927
9 (-)	Empedobacter brevis LMG 4011T HAM	1.324	247
10 (-)	Burkholderia fungorum LMG 20227T HAM	1.314	134537

Analyte32

Analyte Name:	B8
Analyte Description:	
Analyte ID:	3A
Analyte Creation Date/Time:	2019-12-04T13:54:32.272
Applied MSP Library(ies):	BDAL, Filamentous Fungi Library 1.0, Mycobacteria Library 1.0 (bead method), IVD, Listeria
Applied Taxonomy Tree:	

Rank (Quality)	Matched Pattern	Score Value	NCBI Identifier
1 (+++)	Rothia mucilaginosa BK2995_09 ERL	2.37	43675
2 (-)	Rothia dentocariosa CCUG 29965 CCUG	1.697	2047
3 (-)	Rothia mucilaginosa DSM 20445 DSM	1.634	43675
4 (-)	Rothia mucilaginosa DSM 30548 DSM	1.627	43675
5 (-)	Rothia dentocariosa RV_BA1_032010_D LBK	1.52	2047
6 (-)	Sinomonas atrocyanea DSM 20127T DSM	1.412	37927
7 (-)	Rothia mucilaginosa DSM 20446 BRB	1.375	43675
8 (-)	Rhizobium radiobacter B166 UFL	1.342	358
9 (-)	Rothia mucilaginosa CCUG 52532 CCUG	1.337	43675
10 (-)	Arthrobacter stackebrandtii DSM 16005T DSM	1.301	272161

Analyte33

Analyte Name:	B9
Analyte Description:	
Analyte ID:	3B
Analyte Creation Date/Time:	2019-12-04T13:54:32.452
Applied MSP Library(ies):	BDAL, Filamentous Fungi Library 1.0, Mycobacteria Library 1.0 (bead method), IVD, Listeria
Applied Taxonomy Tree:	

Rank (Quality)	Matched Pattern	Score Value	NCBI Identifier
1 (+)	Rothia mucilaginosa DSM 20445 DSM	1.95	43675
2 (+)	Rothia mucilaginosa CCUG 52532 CCUG	1.807	43675
3 (+)	Rothia dentocariosa DSM 43762T DSM	1.754	2047
4 (+)	Rothia mucilaginosa BK2995_09 ERL	1.73	43675
5 (-)	Rothia mucilaginosa CCUG 44966 CCUG	1.641	43675
6 (-)	Rothia mucilaginosa DSM 20446 BRB	1.478	43675
7 (-)	Comamonas testosteroni B337 UFL	1.467	285
8 (-)	Hydrogenophaga flava B339 UFL	1.411	65657
9 (-)	Rothia dentocariosa CCUG 29965 CCUG	1.383	2047
10 (-)	Paenibacillus chinjuensis DSM 15045T DSM	1.37	103815

Analyte34

Analyte Name:	B10
Analyte Description:	
Analyte ID:	3B
Analyte Creation Date/Time:	2019-12-04T13:54:32.214
Applied MSP Library(ies):	BDAL, Filamentous Fungi Library 1.0, Mycobacteria Library 1.0 (bead method), IVD, Listeria
Applied Taxonomy Tree:	

Rank (Quality)	Matched Pattern	Score Value	NCBI Identifier
1 (++)	Rothia mucilaginosa BK2995_09 ERL	2.105	43675
2 (+)	Rothia dentocariosa CCUG 29965 CCUG	1.769	2047
3 (-)	Rothia mucilaginosa CCUG 52532 CCUG	1.581	43675
4 (-)	Morganella morganii 9544_1 CHB	1.533	582
5 (-)	Rothia mucilaginosa DSM 20445 DSM	1.471	43675
6 (-)	Streptococcus gordonii DSM 6777T DSM	1.461	1302
7 (-)	Sinomonas atrocyanea HKI 10432 HKJ	1.46	37927
8 (-)	Rothia mucilaginosa DSM 30548 DSM	1.4	43675
9 (-)	Rothia dentocariosa RV_BA1_032010_D LBK	1.366	2047
10 (-)	Streptococcus infantis DSM 12492T DSM	1.342	68892

Analyte35

Analyte Name:	B11
Analyte Description:	
Analyte ID:	4A
Analyte Creation Date/Time:	2019-12-04T13:54:32.177
Applied MSP Library(ies):	BDAL, Filamentous Fungi Library 1.0, Mycobacteria Library 1.0 (bead method), IVD, Listeria
Applied Taxonomy Tree:	

Rank (Quality)	Matched Pattern	Score Value	NCBI Identifier
1 (-)	Staphylococcus aureus ssp aureus DSM 20231T DSM	1.561	46170
2 (-)	Staphylococcus aureus ssp aureus DSM 3463 DSM	1.482	46170
3 (-)	Staphylococcus aureus ssp aureus DSM 20232 DSM	1.433	46170
4 (-)	Bacillus aquimaris DSM 16205T DSM	1.418	189382
5 (-)	Streptomyces griseus B261 UFL	1.391	1911
6 (-)	Staphylococcus simiae DSM 17636T DSM	1.369	308354
7 (-)	Staphylococcus simiae DSM 17639 DSM	1.349	308354
8 (-)	Lactobacillus helveticus DSM 20075T DSM	1.326	1587
9 (-)	Staphylococcus xylosus FI FLR	1.32	1288
10 (-)	Lactobacillus saerimneri DSM 16049T DSM	1.318	228229

Analyte36

Analyte Name:	B12
Analyte Description:	
Analyte ID:	4A
Analyte Creation Date/Time:	2019-12-04T13:54:31.703
Applied MSP Library(ies):	BDAL, Filamentous Fungi Library 1.0, Mycobacteria Library 1.0 (bead method), IVD, Listeria
Applied Taxonomy Tree:	

Rank (Quality)	Matched Pattern	Score Value	NCBI Identifier
1 (+)	Staphylococcus aureus ssp aureus DSM 3463 DSM	1.755	46170
2 (-)	Staphylococcus simiae DSM 17636T DSM	1.548	308354
3 (-)	Staphylococcus aureus ssp aureus DSM 20232 DSM	1.462	46170
4 (-)	Alicyclobacillus hesperidum DSM 12766 DSM	1.443	89784
5 (-)	Staphylococcus simiae DSM 17639 DSM	1.39	308354
6 (-)	Staphylococcus auricularis DSM 20609 DSM	1.362	29379
7 (-)	Staphylococcus simulans DSM 20723 DSM	1.334	1286
8 (-)	Staphylococcus aureus ssp aureus DSM 4910 DSM	1.323	46170
9 (-)	Staphylococcus warneri Mb18796_1 CHB	1.314	1292
10 (-)	Staphylococcus xylosus FI FLR	1.275	1288

Analyte37

Analyte Name:	B13
Analyte Description:	
Analyte ID:	4B
Analyte Creation Date/Time:	2019-12-04T13:54:32.676
Applied MSP Library(ies):	BDAL, Filamentous Fungi Library 1.0, Mycobacteria Library 1.0 (bead method), IVD, Listeria
Applied Taxonomy Tree:	

Rank (Quality)	Matched Pattern	Score Value	NCBI Identifier
1 (-)	Staphylococcus aureus ssp aureus DSM 4910 DSM	1.666	46170
2 (-)	Staphylococcus aureus ATCC 29213 THL	1.62	1280
3 (-)	Staphylococcus aureus ssp aureus DSM 3463 DSM	1.602	46170
4 (-)	Staphylococcus aureus ssp aureus DSM 20231T DSM	1.557	46170
5 (-)	Staphylococcus aureus ATCC 25923 THL	1.542	1280
6 (-)	Staphylococcus aureus ATCC 33591 THL	1.534	1280
7 (-)	Staphylococcus aureus ATCC 33862 THL	1.498	1280
8 (-)	Corynebacterium ciconiae DSM 44920T DSM	1.487	227319
9 (-)	Staphylococcus simiae DSM 17637 DSM	1.398	308354
10 (-)	Staphylococcus aureus ssp aureus DSM 799 DSM	1.373	46170

Analyte38

Analyte Name:	B14
Analyte Description:	
Analyte ID:	4B
Analyte Creation Date/Time:	2019-12-04T13:54:32.303
Applied MSP Library(ies):	BDAL, Filamentous Fungi Library 1.0, Mycobacteria Library 1.0 (bead method), IVD, Listeria
Applied Taxonomy Tree:	

Rank (Quality)	Matched Pattern	Score Value	NCBI Identifier
1 (+)	Staphylococcus aureus ATCC 33591 THL	1.722	1280
2 (-)	Staphylococcus aureus ssp aureus DSM 4910 DSM	1.554	46170
3 (-)	Staphylococcus aureus ssp aureus DSM 20652 DSM	1.466	46170
4 (-)	Staphylococcus aureus ATCC 29213 THL	1.428	1280
5 (-)	Staphylococcus aureus ssp aureus DSM 20231T DSM	1.396	46170
6 (-)	Rhizobium radiobacter B166 UFL	1.361	358
7 (-)	Staphylococcus schleiferi ssp schleiferi DSM 4807T DSM	1.341	74707
8 (-)	Staphylococcus aureus ssp aureus DSM 3463 DSM	1.336	46170
9 (-)	Candida parapsilosis ATCC 22019 THL	1.328	5480
10 (-)	Staphylococcus felis DSM 7377T DSM	1.325	46127

Analyte39

Analyte Name:	B15
Analyte Description:	
Analyte ID:	5A
Analyte Creation Date/Time:	2019-12-04T13:54:31.999
Applied MSP Library(ies):	BDAL, Filamentous Fungi Library 1.0, Mycobacteria Library 1.0 (bead method), IVD, Listeria
Applied Taxonomy Tree:	

Rank (Quality)	Matched Pattern	Score Value	NCBI Identifier
1 (-)	Arthrobacter mysorens DSM 12798T DSM	1.4	257984
2 (-)	Staphylococcus epidermidis ATCC 14990T THL	1.392	1282
3 (-)	Clostridium novyi 1082_ATCC 17861T BOG	1.386	1542
4 (-)	Azoarcus indigens VB32 MPB	1.358	29545
5 (-)	Pseudomonas syringae ssp syringae LMG 1247T HAM	1.353	317
6 (-)	Stenotrophomonas sp 109_Neb28 NFI	1.334	40323
7 (-)	Pseudomonas graminis DSM 11363T HAM	1.316	158627
8 (-)	Pseudomonas syringae ssp syringae DSM 6693 HAM	1.3	317
9 (-)	Staphylococcus simulans DSM 20324 DSM	1.299	1286
10 (-)	Staphylococcus pasteuri DSM 10656T DSM	1.297	45972

Analyte40

Analyte Name:	B16
Analyte Description:	
Analyte ID:	5A
Analyte Creation Date/Time:	2019-12-04T13:54:31.902
Applied MSP Library(ies):	BDAL, Filamentous Fungi Library 1.0, Mycobacteria Library 1.0 (bead method), IVD, Listeria
Applied Taxonomy Tree:	

Rank (Quality)	Matched Pattern	Score Value	NCBI Identifier
1 (-)	Staphylococcus lutrae DSM 10244T DSM	1.364	155085
2 (-)	Lactobacillus reuteri DSM 20053 DSM	1.343	1598
3 (-)	Pseudomonas stutzeri B367 UFL	1.314	316
4 (-)	Agromyces bracchium HKI 303 DSM 14596T HKJ	1.297	88376
5 (-)	Staphylococcus epidermidis 6b_s ESL	1.294	1282
6 (-)	Agromyces hippuratus HKI 11533_DSM 8598T HKJ	1.291	286438
7 (-)	Staphylococcus aureus ssp aureus DSM 20491 DSM	1.284	46170
8 (-)	Arthrobacter mysorens DSM 12798T DSM	1.274	257984
9 (-)	Staphylococcus aureus ssp aureus DSM 4910 DSM	1.266	46170
10 (-)	Staphylococcus lentus DSM 20352T DSM	1.263	42858

Analyte41

Analyte Name:	B17
Analyte Description:	
Analyte ID:	5B
Analyte Creation Date/Time:	2019-12-04T13:54:32.517
Applied MSP Library(ies):	BDAL, Filamentous Fungi Library 1.0, Mycobacteria Library 1.0 (bead method), IVD, Listeria
Applied Taxonomy Tree:	

Rank (Quality)	Matched Pattern	Score Value	NCBI Identifier
1 (+++)	Enterococcus faecalis DSM 2570 DSM	2.372	1351
2 (++)	Enterococcus faecalis 104575 LDW	2.075	1351
3 (+)	Enterococcus faecalis DSM 20478T JUG	1.971	1351
4 (-)	Enterococcus faecalis DSM 6134 DSM	1.679	1351
5 (-)	Lactobacillus mali DSM 20444T DSM	1.524	1618
6 (-)	Enterococcus faecalis ATCC 7080 THL	1.362	1351
7 (-)	Sporosarcina psychrophila DSM 3T DSM	1.343	1476
8 (-)	Streptococcus pneumoniae ATCC 49619 THL	1.287	1313
9 (-)	Lactococcus lactis ssp lactis DSM 20661 DSM	1.27	1360
10 (-)	Pseudomonas boreopolis LMG 979T HAM	1.262	86183

Analyte42

Analyte Name:	B18
Analyte Description:	
Analyte ID:	5B
Analyte Creation Date/Time:	2019-12-04T13:54:31.820
Applied MSP Library(ies):	BDAL, Filamentous Fungi Library 1.0, Mycobacteria Library 1.0 (bead method), IVD, Listeria
Applied Taxonomy Tree:	

Rank (Quality)	Matched Pattern	Score Value	NCBI Identifier
1 (+++)	Enterococcus faecalis DSM 2570 DSM	2.469	1351
2 (++)	Enterococcus faecalis 104575 LDW	2.166	1351
3 (++)	Enterococcus faecalis DSM 20478T JUG	2.111	1351
4 (+)	Enterococcus faecalis DSM 6134 DSM	1.985	1351
5 (+)	Enterococcus faecalis ATCC 29212 CHB	1.833	1351
6 (-)	Enterococcus caccae DSM 19114T JUG	1.364	317735
7 (-)	Sporosarcina psychrophila DSM 3T DSM	1.326	1476
8 (-)	Nocardia sp MB_9090_05 THL	1.308	1817
9 (-)	Lactobacillus pentosus DSM 20314T DSM	1.281	1589
10 (-)	Lactobacillus malefermentans DSM 20177 DSM	1.269	176292

Analyte43

Analyte Name:	B19
Analyte Description:	
Analyte ID:	7A
Analyte Creation Date/Time:	2019-12-04T13:54:31.840
Applied MSP Library(ies):	BDAL, Filamentous Fungi Library 1.0, Mycobacteria Library 1.0 (bead method), IVD, Listeria
Applied Taxonomy Tree:	

Rank (Quality)	Matched Pattern	Score Value	NCBI Identifier
1 (-)	Stenotrophomonas acidaminiphila DSM 13117T HAM	1.374	128780
2 (-)	Cryptococcus gattii EJB15 CBS	1.25	292817
3 (-)	Staphylococcus auricularis DSM 20609 DSM	1.247	29379
4 (-)	Kytococcus sedentarius IMET 11362T HKJ	1.242	1276
5 (-)	Paenibacillus stellifer DSM 14472T DSM	1.202	169760
6 (-)	Cryptococcus neoformans ATCC 14116 THL	1.182	5207
7 (-)	Bacillus atrophaeus DSM 675 DSM	1.166	1452
8 (-)	Sphingomonas yabuuchiae DSM 14562T HAM	1.164	172044
9 (-)	Lactobacillus amylovorus DSM 20532 DSM	1.15	1604
10 (-)	Brevibacillus formosus DSM 9885T DSM	1.145	54913

Analyte44

Analyte Name:	B20
Analyte Description:	
Analyte ID:	7A
Analyte Creation Date/Time:	2019-12-04T13:54:31.771
Applied MSP Library(ies):	BDAL, Filamentous Fungi Library 1.0, Mycobacteria Library 1.0 (bead method), IVD, Listeria
Applied Taxonomy Tree:	

Rank (Quality)	Matched Pattern	Score Value	NCBI Identifier
1 (-)	Paenibacillus stellifer DSM 14472T DSM	1.482	169760
2 (-)	Paenibacillus zanthoxyli DSM 18202T DSM	1.289	369399
3 (-)	Stenotrophomonas acidaminiphila DSM 13117T HAM	1.243	128780
4 (-)	Paenibacillus durus DSM 5976T DSM	1.24	44251
5 (-)	Weissella viridescens DSM 20248 DSM	1.234	1629
6 (-)	Rothia aeria CCUG 50760 CCUG	1.191	172042
7 (-)	Staphylococcus lugdunensis DSM 4805 DSM	1.179	28035
8 (-)	Clostridium baratii 1018_NCTC 10986 BOG	1.161	1561
9 (-)	Rothia mucilaginosa DSM 30548 DSM	1.155	43675
10 (-)	Agromyces neolithicus HKI 321 HKJ	1.153	269420

Analyte45

Analyte Name:	B21
Analyte Description:	
Analyte ID:	7B
Analyte Creation Date/Time:	2019-12-04T13:54:32.550
Applied MSP Library(ies):	BDAL, Filamentous Fungi Library 1.0, Mycobacteria Library 1.0 (bead method), IVD, Listeria
Applied Taxonomy Tree:	

Rank (Quality)	Matched Pattern	Score Value	NCBI Identifier
1 (++)	Rothia dentocariosa CCUG 29965 CCUG	2.162	2047
2 (+)	Rothia dentocariosa RV_BA1_032010_D LBK	1.981	2047
3 (+)	Rothia dentocariosa DSM 43762T DSM	1.727	2047
4 (-)	Rothia dentocariosa G18709 IBS	1.649	2047
5 (-)	Rothia mucilaginosa BK2995_09 ERL	1.445	43675
6 (-)	Rothia mucilaginosa DSM 20746T DSM	1.383	43675
7 (-)	Streptococcus mitis NRZ 49925 NRZ	1.371	28037
8 (-)	Burkholderia cepacia LMG 2161 HAM	1.344	292
9 (-)	Burkholderia pyrrocinia LMG 14191T HAM	1.336	60550
10 (-)	Streptococcus salivarius DSM 20560T DSM	1.335	1304

Analyte46

Analyte Name:	B22
Analyte Description:	
Analyte ID:	7B
Analyte Creation Date/Time:	2019-12-04T13:54:31.987
Applied MSP Library(ies):	BDAL, Filamentous Fungi Library 1.0, Mycobacteria Library 1.0 (bead method), IVD, Listeria
Applied Taxonomy Tree:	

Rank (Quality)	Matched Pattern	Score Value	NCBI Identifier
1 (+)	Rothia dentocariosa CCUG 29965 CCUG	1.996	2047
2 (+)	Rothia dentocariosa DSM 43762T DSM	1.767	2047
3 (+)	Rothia dentocariosa RV_BA1_032010_D LBK	1.759	2047
4 (-)	Rothia dentocariosa G18709 IBS	1.683	2047
5 (-)	Streptococcus pneumoniae NRZ 31870 NRZ	1.421	1313
6 (-)	Streptococcus pneumoniae V17_201197 MUZ	1.408	1313
7 (-)	Streptococcus mitis NRZ 49039 NRZ	1.408	28037
8 (-)	Rothia mucilaginosa DSM 20746T DSM	1.365	43675
9 (-)	Streptococcus gordonii DSM 6777T DSM	1.359	1302
10 (-)	Chryseobacterium scophthalmum LMG 13028T HAM	1.348	59733

Analyte47

Analyte Name:	B23
Analyte Description:	
Analyte ID:	9A
Analyte Creation Date/Time:	2019-12-04T13:54:32.626
Applied MSP Library(ies):	BDAL, Filamentous Fungi Library 1.0, Mycobacteria Library 1.0 (bead method), IVD, Listeria
Applied Taxonomy Tree:	

Rank (Quality)	Matched Pattern	Score Value	NCBI Identifier
1 (-)	Staphylococcus aureus ATCC 33862 THL	1.646	1280
2 (-)	Staphylococcus simiae DSM 17639 DSM	1.588	308354
3 (-)	Staphylococcus aureus ssp aureus DSM 20231T DSM	1.579	46170
4 (-)	Staphylococcus aureus ssp aureus DSM 3463 DSM	1.522	46170
5 (-)	Staphylococcus aureus ssp aureus DSM 4910 DSM	1.417	46170
6 (-)	Streptomyces avidinii B190 UFL	1.407	1895
7 (-)	Staphylococcus aureus ATCC 29213 THL	1.371	1280
8 (-)	Staphylococcus xylosus FI FLR	1.361	1288
9 (-)	Streptococcus parauberis DSM 6631T DSM	1.333	1348
10 (-)	Staphylococcus auricularis DSM 20609 DSM	1.33	29379

Analyte48

Analyte Name:	B24
Analyte Description:	
Analyte ID:	9A
Analyte Creation Date/Time:	2019-12-04T13:54:32.267
Applied MSP Library(ies):	BDAL, Filamentous Fungi Library 1.0, Mycobacteria Library 1.0 (bead method), IVD, Listeria
Applied Taxonomy Tree:	

Rank (Quality)	Matched Pattern	Score Value	NCBI Identifier
1 (+)	Staphylococcus aureus ssp aureus DSM 20231T DSM	1.829	46170
2 (-)	Staphylococcus aureus ssp aureus DSM 3463 DSM	1.499	46170
3 (-)	Clostridium paraputrificum 1083_ATCC 17796 BOG	1.415	29363
4 (-)	Staphylococcus auricularis DSM 20609 DSM	1.361	29379
5 (-)	Staphylococcus warneri DSM 20036 DSM	1.337	1292
6 (-)	Lactobacillus salivarius DSM 20554 DSM	1.322	1624
7 (-)	Staphylococcus aureus ssp aureus DSM 346 DSM	1.309	46170
8 (-)	Staphylococcus aureus ssp aureus DSM 4910 DSM	1.306	46170
9 (-)	Staphylococcus xylosus FI FLR	1.306	1288
10 (-)	Lactobacillus kimchii DSM 13961T DSM	1.293	103818

Analyte49

Analyte Name:	C1
Analyte Description:	
Analyte ID:	9B
Analyte Creation Date/Time:	2019-12-04T13:54:31.681
Applied MSP Library(ies):	Listeria, BDAL, Filamentous Fungi Library 1.0, Mycobacteria Library 1.0 (bead method), IVD
Applied Taxonomy Tree:	

Rank (Quality)	Matched Pattern	Score Value	NCBI Identifier
1 (-)	Candida glabrata ATCC 2001T THL	1.37	5478
2 (-)	Streptosporangium sibiricum HKI 30 HKJ	1.321	2000
3 (-)	Clostridium novyi A 1025_NCTC 538 BOG	1.319	1542
4 (-)	Clostridium clostridioforme 1021_NCTC 11224T BOG	1.314	1531
5 (-)	Candida dubliniensis SA 108 CBS	1.292	42374
6 (-)	Arthrobacter ramosus IMET 10685T HKJ	1.279	1672
7 (-)	Arthrobacter tecti DSM 16407T DSM	1.27	271433
8 (-)	Staphylococcus aureus ssp aureus DSM 20232 DSM	1.26	46170
9 (-)	Lactobacillus fermentum DSM 20391 DSM	1.238	1613
10 (-)	Pseudomonas veronii B560 UFL	1.238	76761

Analyte50

Analyte Name:	C2
Analyte Description:	
Analyte ID:	9B
Analyte Creation Date/Time:	2019-12-04T13:54:32.563
Applied MSP Library(ies):	BDAL, Filamentous Fungi Library 1.0, Mycobacteria Library 1.0 (bead method), IVD, Listeria
Applied Taxonomy Tree:	

Rank (Quality)	Matched Pattern	Score Value	NCBI Identifier
1 (+)	Staphylococcus aureus ssp aureus DSM 20231T DSM	1.977	46170
2 (+)	Staphylococcus aureus ssp aureus DSM 3463 DSM	1.794	46170
3 (+)	Staphylococcus aureus ssp aureus DSM 799 DSM	1.705	46170
4 (-)	Staphylococcus simiae DSM 17636T DSM	1.465	308354
5 (-)	Staphylococcus capitis ssp capitis DSM 20325 DSM	1.377	72758
6 (-)	Staphylococcus simiae DSM 17638 DSM	1.322	308354
7 (-)	Staphylococcus capitis ssp capitis DSM 20326T DSM	1.298	72758
8 (-)	Staphylococcus aureus ssp aureus DSM 20232 DSM	1.28	46170
9 (-)	Staphylococcus xylosus FI FLR	1.275	1288
10 (-)	Streptococcus ferus DSM 20646T DSM	1.262	1345

Analyte51

Analyte Name:	C3
Analyte Description:	
Analyte ID:	10A
Analyte Creation Date/Time:	2019-12-04T13:54:32.144
Applied MSP Library(ies):	Listeria, IVD, Mycobacteria Library 1.0 (bead method), Filamentous Fungi Library 1.0, BDAL
Applied Taxonomy Tree:	

Rank (Quality)	Matched Pattern	Score Value	NCBI Identifier
1 (-)	Rothia mucilaginosa CCUG 31189 CCUG	1.51	43675
2 (-)	Hydrogenophaga flava B339 UFL	1.452	65657
3 (-)	Rothia mucilaginosa CCUG 44966 CCUG	1.426	43675
4 (-)	Streptococcus salivarius DSM 20560T DSM	1.42	1304
5 (-)	Rothia mucilaginosa CCUG 52532 CCUG	1.358	43675
6 (-)	Janthinobacterium lividum CIP 106720T HAM	1.349	29581
7 (-)	Streptococcus salivarius DSM 20560T BRB	1.336	1304
8 (-)	Rhizobium radiobacter B166 UFL	1.323	358
9 (-)	Mycobacterium smegmatis 19 PGM	1.318	1772
10 (-)	Rothia mucilaginosa BK2995_09 ERL	1.316	43675

Analyte52

Analyte Name:	C4
Analyte Description:	
Analyte ID:	10A
Analyte Creation Date/Time:	2019-12-04T13:54:32.309
Applied MSP Library(ies):	BDAL, Filamentous Fungi Library 1.0, Mycobacteria Library 1.0 (bead method), IVD, Listeria
Applied Taxonomy Tree:	

Rank (Quality)	Matched Pattern	Score Value	NCBI Identifier
1 (-)	Lactobacillus satsumensis DSM 16230T DSM	1.494	259059
2 (-)	Fusarium dimerum_BB 080809 VML	1.429	123269315
3 (-)	Thauera linaloolentis 47Lol MPB	1.392	76112
4 (-)	Lactobacillus plantarum DSM 20205 DSM	1.38	1590
5 (-)	Lactobacillus coryniformis ssp coryniformis DSM 20007 DSM	1.371	115541
6 (-)	Lactobacillus pentosus DSM 20199 DSM	1.346	1589
7 (-)	Lactobacillus plantarum ssp plantarum DSM 20174T DSM	1.341	337330
8 (-)	Clostridium tertium 1048_NCTC 541 BOG	1.32	1559
9 (-)	Staphylococcus fleurettii DSM 13212T DSM	1.296	150056
10 (-)	Pseudomonas mandelii CIP 105273T HAM	1.294	75612

Analyte53

Analyte Name:	C5
Analyte Description:	
Analyte ID:	10B
Analyte Creation Date/Time:	2019-12-04T13:54:32.109
Applied MSP Library(ies):	BDAL, Filamentous Fungi Library 1.0, Mycobacteria Library 1.0 (bead method), IVD, Listeria
Applied Taxonomy Tree:	

Rank (Quality)	Matched Pattern	Score Value	NCBI Identifier
1 (-)	Clostridium novyi A 1025_NCTC 538 BOG	1.391	1542
2 (-)	Rothia mucilaginosa CCUG 44966 CCUG	1.273	43675
3 (-)	Lactobacillus johnsonii DSM 20553 DSM	1.27	33959
4 (-)	Lactobacillus gasseri DSM 20077 DSM	1.216	1596
5 (-)	Rhodotorula mucilaginosa CBS 316T CBS	1.21	5537
6 (-)	Paenibacillus mendelii DSM 19248T DSM	1.205	206163
7 (-)	Staphylococcus simulans CCM 2724 CCM	1.204	1286
8 (-)	Sphingomonas adhaesiva DSM 7418T HAM	1.197	28212
9 (-)	Ideonella dechloratans CCUG 30977T PAH	1.192	36863
10 (-)	Corynebacterium amycolatum 100_28_B77962_42 IBS	1.189	43765

Analyte54

Analyte Name:	C6
Analyte Description:	
Analyte ID:	10B
Analyte Creation Date/Time:	2019-12-04T13:54:32.023
Applied MSP Library(ies):	BDAL, Filamentous Fungi Library 1.0, Mycobacteria Library 1.0 (bead method), IVD, Listeria
Applied Taxonomy Tree:	

Rank (Quality)	Matched Pattern	Score Value	NCBI Identifier
1 (-)	Corynebacterium confusum DSM 44384T DSM	1.394	71254
2 (-)	Cellulomonas gelida IMET 11078 HKJ	1.394	1712
3 (-)	Lactobacillus saerimneri DSM 16049T DSM	1.382	228229
4 (-)	Burkholderia pyrrocinia LMG 14191T HAM	1.335	60550
5 (-)	Arthrobacter nicotinovorans DSM 420T DSM	1.319	29320
6 (-)	Clostridium novyi A 1025_NCTC 538 BOG	1.311	1542
7 (-)	Nocardia cyriacigeorgica 121106_21 HUA	1.288	135487
8 (-)	Jonesia denitrificans DSM 20603T DSM	1.273	43674
9 (-)	Rhizobium radiobacter B170 UFL	1.264	358
10 (-)	Burkholderia cepacia_Group 18875_1 CHB	1.257	292

Analyte55

Analyte Name:	C7
Analyte Description:	
Analyte ID:	16A
Analyte Creation Date/Time:	2019-12-04T13:54:32.037
Applied MSP Library(ies):	BDAL, Filamentous Fungi Library 1.0, Mycobacteria Library 1.0 (bead method), IVD, Listeria
Applied Taxonomy Tree:	

Rank (Quality)	Matched Pattern	Score Value	NCBI Identifier
1 (+)	Candida albicans DSM 1577 DSM	1.924	5476
2 (+)	Candida albicans DSM 5817 DSM	1.899	5476
3 (+)	Candida albicans DSM 11949 DSM	1.831	5476
4 (+)	Candida albicans DSM 6569 DSM	1.766	5476
5 (-)	Candida albicans DSM 3454 DSM	1.692	5476
6 (-)	Candida albicans DSM 11945 DSM	1.67	5476
7 (-)	Candida albicans ATCC 10231 THL	1.639	5476
8 (-)	Candida albicans_(africana) VML	1.628	241526
9 (-)	Candida albicans RV_D VML	1.6	5476
10 (-)	Candida albicans DSM 11943 DSM	1.594	5476

Analyte56

Analyte Name:	C8
Analyte Description:	
Analyte ID:	16A
Analyte Creation Date/Time:	2019-12-04T13:54:31.621
Applied MSP Library(ies):	BDAL, Filamentous Fungi Library 1.0, Mycobacteria Library 1.0 (bead method), IVD, Listeria
Applied Taxonomy Tree:	

Rank (Quality)	Matched Pattern	Score Value	NCBI Identifier
1 (-)	Candida albicans DSM 11949 DSM	1.659	5476
2 (-)	Candida albicans DSM 1577 DSM	1.658	5476
3 (-)	Candida albicans DSM 3454 DSM	1.622	5476
4 (-)	Candida albicans DSM 5817 DSM	1.616	5476
5 (-)	Candida albicans RV_D VML	1.61	5476
6 (-)	Candida albicans ATCC 10231 THL	1.496	5476
7 (-)	Candida albicans DSM 11945 DSM	1.485	5476
8 (-)	Clostridium bifermentans 2274_CCUG 35556 A BOG	1.462	1490
9 (-)	Candida albicans DSM 1665 DSM	1.458	5476
10 (-)	Candida albicans DSM 11943 DSM	1.421	5476

Analyte57

Analyte Name:	C9
Analyte Description:	
Analyte ID:	16B
Analyte Creation Date/Time:	2019-12-04T13:54:31.708
Applied MSP Library(ies):	BDAL, Filamentous Fungi Library 1.0, Mycobacteria Library 1.0 (bead method), IVD, Listeria
Applied Taxonomy Tree:	

Rank (Quality)	Matched Pattern	Score Value	NCBI Identifier
1 (+)	Filifactor villosus 1051_NCTC 11220T BOG	1.72	29374
2 (-)	Pichia occidentalis CBS 1910 CBS	1.415	54552
3 (-)	Staphylococcus schleiferi ssp schleiferi DSM 4807T DSM	1.395	74707
4 (-)	Clostridium bifermentans 2274_CCUG 35556 A BOG	1.38	1490
5 (-)	Vibrio vulnificus CCM 2840 CCM	1.365	672
6 (-)	Streptomyces lavendulae B264 UFL	1.35	1914
7 (-)	Mycobacterium tuberculosis W336 R_880 PGM	1.316	1773
8 (-)	Candida guilliermondii CBS 566 CBS	1.314	4929
9 (-)	Lactobacillus sakei ssp sakei DSM 20017T DSM	1.311	214326
10 (-)	Staphylococcus simulans DSM 20324 DSM	1.308	1286

Analyte58

Analyte Name:	C10
Analyte Description:	
Analyte ID:	16B
Analyte Creation Date/Time:	2019-12-04T13:54:32.714
Applied MSP Library(ies):	BDAL, Filamentous Fungi Library 1.0, Mycobacteria Library 1.0 (bead method), IVD, Listeria
Applied Taxonomy Tree:	

Rank (Quality)	Matched Pattern	Score Value	NCBI Identifier
1 (-)	Staphylococcus vitulinus DSM 15615T DSM	1.436	71237
2 (-)	Lactobacillus paralimentarius DSM 13238T DSM	1.4	83526
3 (-)	Trueperella pyogenes DSM 20630T DSM	1.392	1661
4 (-)	Thauera mechernichensis Tl1 MPB	1.36	82788
5 (-)	Erysipelothrix rhusiopathiae DSM 5055T DSM	1.358	1648
6 (-)	Candida parapsilosis ATCC 22019 THL	1.343	5480
7 (-)	Clostridium difficile 1020_NCTC 11206 BOG	1.335	1496
8 (-)	Lactobacillus paracasei ssp paracasei DSM 5622T DSM	1.333	47714
9 (-)	Erysipelothrix rhusiopathiae DSM 5058 DSM	1.331	1648
10 (-)	Candida dubliniensis SA 121 CBS	1.31	42374

Analyte59

Analyte Name:	C11
Analyte Description:	
Analyte ID:	17
Analyte Creation Date/Time:	2019-12-04T13:54:32.749
Applied MSP Library(ies):	BDAL, Filamentous Fungi Library 1.0, Mycobacteria Library 1.0 (bead method), IVD, Listeria
Applied Taxonomy Tree:	

Rank (Quality)	Matched Pattern	Score Value	NCBI Identifier
1 (-)	Streptococcus salivarius 0807M25049501 IBS	1.556	1304
2 (-)	Streptococcus salivarius DSM 20560T DSM	1.553	1304
3 (-)	Neisseria meningitidis 639 PGM	1.54	487
4 (-)	Neisseria meningitidis C1 2 PGM	1.501	487
5 (-)	Clostridium bifermentans 2274_CCUG 35556 A BOG	1.452	1490
6 (-)	Pichia occidentalis CBS 1910 CBS	1.424	54552
7 (-)	Pseudomonas syringae ssp syringae LMG 1247T HAM	1.422	317
8 (-)	Streptococcus salivarius DSM 20560T BRB	1.395	1304
9 (-)	Clostridium cadaveris 1074_ATCC 25783T BOG	1.389	1529
10 (-)	Filifactor villosus 1051_NCTC 11220T BOG	1.357	29374

Analyte60

Analyte Name:	C12
Analyte Description:	
Analyte ID:	17
Analyte Creation Date/Time:	2019-12-04T13:54:32.636
Applied MSP Library(ies):	BDAL, Filamentous Fungi Library 1.0, Mycobacteria Library 1.0 (bead method), IVD, Listeria
Applied Taxonomy Tree:	

Rank (Quality)	Matched Pattern	Score Value	NCBI Identifier
1 (-)	Streptococcus salivarius 0807M25049501 IBS	1.534	1304
2 (-)	Pseudomonas mandelii CIP 105273T HAM	1.414	75612
3 (-)	Streptococcus downei DSM 5635T DSM	1.384	1317
4 (-)	Streptococcus cristatus DSM 8249T DSM	1.379	45634
5 (-)	Lactobacillus sakei DSM 6333 DSM	1.314	1599
6 (-)	Lactobacillus sakei ssp carnosus DSM 15831T DSM	1.303	214325
7 (-)	Sphingobacterium spiritivorum DSM 11722T HAM	1.296	258
8 (-)	Neisseria weaveri DSM 17688T DSM	1.291	28091
9 (-)	Lactobacillus sharpeae DSM 20506 DSM	1.287	1626
10 (-)	Streptococcus sobrinus DSM 20742T DSM	1.282	1310

Analyte61

Analyte Name:	C13
Analyte Description:	
Analyte ID:	19
Analyte Creation Date/Time:	2019-12-04T13:54:32.428
Applied MSP Library(ies):	Listeria, BDAL, Filamentous Fungi Library 1.0, Mycobacteria Library 1.0 (bead method), IVD
Applied Taxonomy Tree:	

Rank (Quality)	Matched Pattern	Score Value	NCBI Identifier
1 (-)	Streptomyces badius B192 UFL	1.39	1941
2 (-)	Staphylococcus epidermidis DSM 1798 DSM	1.383	1282
3 (-)	Streptomyces albus B262 UFL	1.365	1888
4 (-)	Lactobacillus paracasei ssp paracasei DSM 8742 DSM	1.343	47714
5 (-)	Aspergillus versicolor F68 RLH	1.337	123269315
6 (-)	Trichosporon sp 122 PSB	1.328	5552
7 (-)	Staphylococcus capitis ssp capitis DSM 20326T DSM	1.328	72758
8 (-)	Lactobacillus saerimneri DSM 16049T DSM	1.319	228229
9 (-)	Myroides odoratus DSM 2801T HAM	1.309	256
10 (-)	Streptomyces lavendulae B264 UFL	1.302	1914

Analyte62

Analyte Name:	C14
Analyte Description:	
Analyte ID:	19
Analyte Creation Date/Time:	2019-12-04T13:54:32.690
Applied MSP Library(ies):	BDAL, Filamentous Fungi Library 1.0, Mycobacteria Library 1.0 (bead method), IVD, Listeria
Applied Taxonomy Tree:	

Rank (Quality)	Matched Pattern	Score Value	NCBI Identifier
1 (-)	Lactobacillus paracasei ssp paracasei DSM 5622T DSM	1.523	47714
2 (-)	Clostridium difficile MB_7869_05 THL	1.511	1496
3 (-)	Filifactor villosus 1051_NCTC 11220T BOG	1.479	29374
4 (-)	Lactobacillus satsumensis DSM 16230T DSM	1.476	259059
5 (-)	Clostridium difficile MB_294_05 THL	1.446	1496
6 (-)	Bacillus gibsonii DSM 8722T DSM	1.411	79881
7 (-)	Thauera phenylacetica B4P MPB	1.386	164400
8 (-)	Elizabethkingia miricola DSM 14571T HAM	1.381	172045
9 (-)	Staphylococcus epidermidis ATCC 12228 CHB	1.372	1282
10 (-)	Clostridium septicum 1026_NCTC 547T BOG	1.37	1504

Analyte63

Analyte Name:	C15
Analyte Description:	
Analyte ID:	20
Analyte Creation Date/Time:	2019-12-04T13:54:31.933
Applied MSP Library(ies):	BDAL, Filamentous Fungi Library 1.0, Mycobacteria Library 1.0 (bead method), IVD, Listeria
Applied Taxonomy Tree:	

Rank (Quality)	Matched Pattern	Score Value	NCBI Identifier
1 (-)	Streptomyces lavendulae B264 UFL	1.459	1914
2 (-)	Lactobacillus equi DSM 15833T DSM	1.409	137357
3 (-)	Candida kefyr CBS 834 CBS	1.398	374272
4 (-)	Streptomyces avidinii B190 UFL	1.384	1895
5 (-)	Fusarium proliferatum_CC3 080909 VML	1.377	123269315
6 (-)	Aromatoleum buckelii U120 MPB	1.375	200254
7 (-)	Weissella minor DSM 20014T DSM	1.359	1620
8 (-)	Arthrobacter ramosus IMET 10685T HKJ	1.357	1672
9 (-)	Filifactor villosus 1051_NCTC 11220T BOG	1.354	29374
10 (-)	Nocardia sp N394 IBS	1.346	1817

Analyte64

Analyte Name:	C16
Analyte Description:	
Analyte ID:	20
Analyte Creation Date/Time:	2019-12-04T13:54:31.573
Applied MSP Library(ies):	BDAL, Filamentous Fungi Library 1.0, Mycobacteria Library 1.0 (bead method), IVD, Listeria
Applied Taxonomy Tree:	

Rank (Quality)	Matched Pattern	Score Value	NCBI Identifier
1 (-)	Filifactor villosus 1051_NCTC 11220T BOG	1.526	29374
2 (-)	Mycobacterium bovis BCG 1878 PGM	1.488	1765
3 (-)	Lactobacillus fructivorans DSM 20203T DSM	1.406	1614
4 (-)	Staphylococcus saprophyticus ssp saprophyticus CCM 2602 CCM	1.4	29385
5 (-)	Lactobacillus sakei ssp sakei DSM 20017T DSM	1.395	214326
6 (-)	Mycobacterium bovis Bovinus An_1 PGM	1.39	1765
7 (-)	Lactobacillus plantarum DSM 20205 DSM	1.375	1590
8 (-)	Agromyces rhizospherae HKI 302_DSM 14597T HKJ	1.368	88374
9 (-)	Lactobacillus paracasei ssp paracasei DSM 20207 DSM	1.354	47714
10 (-)	Trichosporon cutaneum 120 PSB	1.343	5554

Analyte65

Analyte Name:	C17
Analyte Description:	
Analyte ID:	21
Analyte Creation Date/Time:	2019-12-04T13:54:31.950
Applied MSP Library(ies):	BDAL, Filamentous Fungi Library 1.0, Mycobacteria Library 1.0 (bead method), IVD, Listeria
Applied Taxonomy Tree:	

Rank (Quality)	Matched Pattern	Score Value	NCBI Identifier
1 (-)	Filifactor villosus 1051_NCTC 11220T BOG	1.617	29374
2 (-)	Staphylococcus epidermidis 4b_r ESL	1.507	1282
3 (-)	Streptomyces hirsutus B267 UFL	1.419	35620
4 (-)	Aeromonas veronii CECT 4199T DSM	1.385	654
5 (-)	Aeromonas veronii CECT 4257T DSM	1.382	654
6 (-)	Staphylococcus simulans DSM 20324 DSM	1.373	1286
7 (-)	Paecilomyces lilacinus 85 VML	1.368	33203
8 (-)	Candida parapsilosis ATCC 22019 THL	1.339	5480
9 (-)	Lactobacillus satsumensis DSM 16230T DSM	1.334	259059
10 (-)	Streptomyces lavendulae B264 UFL	1.333	1914

Analyte66

Analyte Name:	C18
Analyte Description:	
Analyte ID:	21
Analyte Creation Date/Time:	2019-12-04T13:54:32.032
Applied MSP Library(ies):	BDAL, Filamentous Fungi Library 1.0, Mycobacteria Library 1.0 (bead method), IVD, Listeria
Applied Taxonomy Tree:	

Rank (Quality)	Matched Pattern	Score Value	NCBI Identifier
1 (-)	**no peaks found**	< 0	-
2 (-)	**no peaks found**	< 0	-
3 (-)	**no peaks found**	< 0	-
4 (-)	**no peaks found**	< 0	-
5 (-)	**no peaks found**	< 0	-
6 (-)	**no peaks found**	< 0	-
7 (-)	**no peaks found**	< 0	-
8 (-)	**no peaks found**	< 0	-
9 (-)	**no peaks found**	< 0	-
10 (-)	**no peaks found**	< 0	-

Analyte67

Analyte Name:	C19
Analyte Description:	
Analyte ID:	24
Analyte Creation Date/Time:	2019-12-04T13:54:31.781
Applied MSP Library(ies):	BDAL, Filamentous Fungi Library 1.0, Mycobacteria Library 1.0 (bead method), IVD, Listeria
Applied Taxonomy Tree:	

Rank (Quality)	Matched Pattern	Score Value	NCBI Identifier
1 (-)	Lactobacillus paracasei ssp paracasei DSM 20207 DSM	1.544	47714
2 (-)	Lactobacillus paralimentarius DSM 13238T DSM	1.458	83526
3 (-)	Lactobacillus fructivorans DSM 20203T DSM	1.451	1614
4 (-)	Lactobacillus plantarum DSM 20205 DSM	1.45	1590
5 (-)	Acinetobacter haemolyticus LMG 1033 HAM	1.413	29430
6 (-)	Shewanella algae DSM 9167T HAM	1.396	38313
7 (-)	Lactobacillus paracasei ssp tolerans DSM 20258T DSM	1.378	113557
8 (-)	Clostridium bifermentans 2274_CCUG 35556 A BOG	1.375	1490
9 (-)	Lactobacillus sakei ssp sakei DSM 20017T DSM	1.36	214326
10 (-)	Cryptococcus neoformans ATCC 14116 THL	1.348	5207

Analyte68

Analyte Name:	C20
Analyte Description:	
Analyte ID:	24
Analyte Creation Date/Time:	2019-12-04T13:54:32.654
Applied MSP Library(ies):	BDAL, Filamentous Fungi Library 1.0, Mycobacteria Library 1.0 (bead method), IVD, Listeria
Applied Taxonomy Tree:	

Rank (Quality)	Matched Pattern	Score Value	NCBI Identifier
1 (-)	Streptomyces lavendulae B264 UFL	1.56	1914
2 (-)	Streptomyces phaeochromogenes B265 UFL	1.443	1923
3 (-)	Mycobacterium tuberculosis W148 R_722_HI PGM	1.406	1773
4 (-)	Paracoccus versutus B352 UFL	1.366	34007
5 (-)	Mycobacterium tuberculosis W336 R_880 PGM	1.339	1773
6 (-)	Acinetobacter calcoaceticus B388 UFL	1.339	471
7 (-)	Actinocorallia libanotica B246 UFL	1.338	46162
8 (-)	Mycobacterium chelonae ssp chelonae DSM 43486 DSM b	1.33	122893407
9 (-)	Candida lambica CBS 603 CBS	1.329	53655
10 (-)	Mycobacterium abscessus ssp abscessus DSM 44196T DSM b	1.311	122893407

Analyte69

Analyte Name:	C21
Analyte Description:	
Analyte ID:	25A
Analyte Creation Date/Time:	2019-12-04T13:54:32.462
Applied MSP Library(ies):	BDAL, Filamentous Fungi Library 1.0, Mycobacteria Library 1.0 (bead method), IVD, Listeria
Applied Taxonomy Tree:	

Rank (Quality)	Matched Pattern	Score Value	NCBI Identifier
1 (-)	Pichia occidentalis CBS 1910 CBS	1.604	54552
2 (-)	Colletotrichum gloeosporioides CBS 100471 CBS	1.507	474922
3 (-)	Filifactor villosus 1051_NCTC 11220T BOG	1.409	29374
4 (-)	Lactobacillus satsumensis DSM 16230T DSM	1.353	259059
5 (-)	Candida guilliermondii CBS 566 CBS	1.342	4929
6 (-)	Vibrio furnissii LMG 7910T HAM	1.34	29494
7 (-)	Lactobacillus paracasei ssp paracasei DSM 5622T DSM	1.322	47714
8 (-)	Shewanella algae DSM 9167T HAM	1.315	38313
9 (-)	Staphylococcus aureus ssp aureus DSM 20232 DSM	1.311	46170
10 (-)	Candida parapsilosis ATCC 22019 THL	1.301	5480

Analyte70

Analyte Name:	C22
Analyte Description:	
Analyte ID:	25A
Analyte Creation Date/Time:	2019-12-04T13:54:31.922
Applied MSP Library(ies):	BDAL, Filamentous Fungi Library 1.0, Mycobacteria Library 1.0 (bead method), IVD, Listeria
Applied Taxonomy Tree:	

Rank (Quality)	Matched Pattern	Score Value	NCBI Identifier
1 (-)	Thauera aromatica K172 MPB	1.359	44139
2 (-)	Agromyces salentinus HKI 320_DSM 16198T HKJ	1.359	269421
3 (-)	Candida allociferrii VML	1.346	191333
4 (-)	Lactobacillus plantarum DSM 13273 DSM	1.345	1590
5 (-)	Curtobacterium luteum HKI 10360 HKJ	1.321	33881
6 (-)	Candida guilliermondii CBS 566 CBS	1.313	4929
7 (-)	Candida tropicalis ATCC 13803 THL	1.31	5482
8 (-)	Lactobacillus plantarum DSM 20205 DSM	1.298	1590
9 (-)	Bacteroides fragilis MB_5088_05 THL	1.291	817
10 (-)	Arthrobacter ramosus IMET 10685T HKJ	1.286	1672

Analyte71

Analyte Name:	C23
Analyte Description:	
Analyte ID:	25B
Analyte Creation Date/Time:	2019-12-04T13:54:32.406
Applied MSP Library(ies):	BDAL, Filamentous Fungi Library 1.0, Mycobacteria Library 1.0 (bead method), IVD, Listeria
Applied Taxonomy Tree:	

Rank (Quality)	Matched Pattern	Score Value	NCBI Identifier
1 (+)	Rothia dentocariosa DSM 43762T DSM	1.838	2047
2 (-)	Rothia dentocariosa CCUG 29965 CCUG	1.592	2047
3 (-)	Candida albicans DSM 1665 DSM	1.475	5476
4 (-)	Candida albicans DSM 6659 DSM	1.438	5476
5 (-)	Paenibacillus glucanolyticus DSM 5162T DSM	1.429	59843
6 (-)	Candida albicans DSM 5817 DSM	1.407	5476
7 (-)	Rothia mucilaginosa DSM 20446 DSM	1.406	43675
8 (-)	Candida albicans DSM 6569 DSM	1.389	5476
9 (-)	Paenibacillus brasilensis DSM 14914T DSM	1.381	128574
10 (-)	Rothia mucilaginosa DSM 20445 DSM	1.379	43675

Analyte72

Analyte Name:	C24
Analyte Description:	
Analyte ID:	25B
Analyte Creation Date/Time:	2019-12-04T13:54:32.080
Applied MSP Library(ies):	BDAL, Filamentous Fungi Library 1.0, Mycobacteria Library 1.0 (bead method), IVD, Listeria
Applied Taxonomy Tree:	

Rank (Quality)	Matched Pattern	Score Value	NCBI Identifier
1 (+)	Rothia dentocariosa DSM 43762T DSM	1.757	2047
2 (-)	Rothia dentocariosa CCUG 29965 CCUG	1.625	2047
3 (-)	Rothia dentocariosa RV_BA1_032010_D LBK	1.523	2047
4 (-)	Lactobacillus jensenii DSM 20557T DSM	1.445	109790
5 (-)	Paenibacillus macerans DSM 1574 DSM	1.356	44252
6 (-)	Streptococcus gordonii DSM 6777T DSM	1.349	1302
7 (-)	Paenibacillus phyllosphaerae DSM 17399T DSM	1.333	274593
8 (-)	Rothia aeria NO_11 HUA	1.314	172042
9 (-)	Weissella viridescens DSM 20248 DSM	1.311	1629
10 (-)	Candida albicans DSM 11949 DSM	1.303	5476

Analyte73

Analyte Name:	D1
Analyte Description:	
Analyte ID:	26A
Analyte Creation Date/Time:	2019-12-04T13:54:32.480
Applied MSP Library(ies):	BDAL, Filamentous Fungi Library 1.0, Mycobacteria Library 1.0 (bead method), IVD, Listeria
Applied Taxonomy Tree:	

Rank (Quality)	Matched Pattern	Score Value	NCBI Identifier
1 (-)	Agromyces rhizospherae HKI 302_DSM 14597T HKJ	1.449	88374
2 (-)	Nocardiopsis alba DSM 43377T DSM	1.391	53437
3 (-)	Agromyces bracchium HKI 303 DSM 14596T HKJ	1.384	88376
4 (-)	Lactobacillus paracasei ssp paracasei DSM 5457 DSM	1.377	47714
5 (-)	Lactobacillus fructivorans DSM 20203T DSM	1.367	1614
6 (-)	Streptomyces lavendulae B264 UFL	1.358	1914
7 (-)	Candida lambica CBS 603 CBS	1.356	53655
8 (-)	Aromatoleum bremensis PbN1 MPB	1.353	12960
9 (-)	Arthrobacter sulfonivorans DSM 14002T DSM	1.351	121292
10 (-)	Filifactor villosus 1051_NCTC 11220T BOG	1.336	29374

Analyte74

Analyte Name:	D2
Analyte Description:	
Analyte ID:	26A
Analyte Creation Date/Time:	2019-12-04T13:54:31.825
Applied MSP Library(ies):	BDAL, Filamentous Fungi Library 1.0, Mycobacteria Library 1.0 (bead method), IVD, Listeria
Applied Taxonomy Tree:	

Rank (Quality)	Matched Pattern	Score Value	NCBI Identifier
1 (-)	Paracoccus versutus B352 UFL	1.529	34007
2 (-)	Pichia occidentalis CBS 1910 CBS	1.501	54552
3 (-)	Staphylococcus epidermidis 4b_r ESL	1.496	1282
4 (-)	Candida lambica CBS 603 CBS	1.429	53655
5 (-)	Staphylococcus epidermidis 6b_s ESL	1.319	1282
6 (-)	Clostridium cadaveris 1074_ATCC 25783T BOG	1.309	1529
7 (-)	Avibacterium avium DSM 18557T DSM	1.307	751
8 (-)	Candida albicans RV_D VML	1.297	5476
9 (-)	Lactobacillus coryniformis ssp coryniformis DSM 20007 DSM	1.28	115541
10 (-)	Agromyces fucosus HKI 11529_DSM 8597T HKJ	1.271	41985

Analyte75

Analyte Name:	D3
Analyte Description:	
Analyte ID:	26B
Analyte Creation Date/Time:	2019-12-04T13:54:31.892
Applied MSP Library(ies):	BDAL, Filamentous Fungi Library 1.0, Mycobacteria Library 1.0 (bead method), IVD, Listeria
Applied Taxonomy Tree:	

Rank (Quality)	Matched Pattern	Score Value	NCBI Identifier
1 (-)	Pseudomonas mandelii CIP 105273T HAM	1.438	75612
2 (-)	Sporosarcina psychrophila DSM 3T DSM	1.422	1476
3 (-)	Streptococcus orisratti DSM 15617T DSM	1.402	114652
4 (-)	Lactobacillus sharpeae DSM 20504 DSM	1.385	1626
5 (-)	Chryseobacterium scophthalmum LMG 13028T HAM	1.382	59733
6 (-)	Aeromonas salmonicida ssp salmonicida CECT 894T DSM	1.37	29491
7 (-)	Lactobacillus malefermentans DSM 20570 DSM	1.351	176292
8 (-)	Trichoderma koningii_BB F59_2 LLH	1.342	123269315
9 (-)	Streptococcus cristatus DSM 8249T DSM	1.329	45634
10 (-)	Streptococcus vestibularis DSM 5636T DSM	1.327	1343

Analyte76

Analyte Name:	D4
Analyte Description:	
Analyte ID:	26B
Analyte Creation Date/Time:	2019-12-04T13:54:32.154
Applied MSP Library(ies):	BDAL, Filamentous Fungi Library 1.0, Mycobacteria Library 1.0 (bead method), IVD, Listeria
Applied Taxonomy Tree:	

Rank (Quality)	Matched Pattern	Score Value	NCBI Identifier
1 (-)	Sporosarcina psychrophila DSM 3T DSM	1.504	1476
2 (-)	Streptococcus orisratti DSM 15617T DSM	1.455	114652
3 (-)	Streptococcus cristatus DSM 8249T DSM	1.419	45634
4 (-)	Streptococcus pneumoniae ATCC 49619 THL	1.391	1313
5 (-)	Streptococcus salivarius 0807M25049501 IBS	1.312	1304
6 (-)	Neisseria meningitidis C1 2 PGM	1.311	487
7 (-)	Streptococcus parauberis DSM 6631T DSM	1.297	1348
8 (-)	Propionibacterium acnes DSM 1897T DSM	1.291	1747
9 (-)	Streptococcus parauberis DSM 6632 DSM	1.279	1348
10 (-)	Streptococcus hyointestinalis DSM 20770T DSM	1.266	1337

Analyte77

Analyte Name:	D5
Analyte Description:	
Analyte ID:	27A
Analyte Creation Date/Time:	2019-12-04T13:54:31.617
Applied MSP Library(ies):	BDAL, Filamentous Fungi Library 1.0, Mycobacteria Library 1.0 (bead method), IVD, Listeria
Applied Taxonomy Tree:	

Rank (Quality)	Matched Pattern	Score Value	NCBI Identifier
1 (-)	Staphylococcus aureus ATCC 33591 THL	1.652	1280
2 (-)	Staphylococcus simiae DSM 17636T DSM	1.495	308354
3 (-)	Staphylococcus simiae DSM 17637 DSM	1.47	308354
4 (-)	Candida guilliermondii CBS 566 CBS	1.342	4929
5 (-)	Lactobacillus satsumensis DSM 16230T DSM	1.341	259059
6 (-)	Clostridium novyi A 1025_NCTC 538 BOG	1.339	1542
7 (-)	Candida tropicalis ATCC 13803 THL	1.325	5482
8 (-)	Arthrobacter ramosus IMET 10685T HKJ	1.324	1672
9 (-)	Lactobacillus paracasei ssp paracasei DSM 5457 DSM	1.308	47714
10 (-)	Candida parapsilosis ATCC 22019 THL	1.297	5480

Analyte78

Analyte Name:	D6
Analyte Description:	
Analyte ID:	27A
Analyte Creation Date/Time:	2019-12-04T13:54:31.690
Applied MSP Library(ies):	BDAL, Filamentous Fungi Library 1.0, Mycobacteria Library 1.0 (bead method), IVD, Listeria
Applied Taxonomy Tree:	

Rank (Quality)	Matched Pattern	Score Value	NCBI Identifier
1 (-)	Staphylococcus aureus ssp aureus DSM 3463 DSM	1.475	46170
2 (-)	Staphylococcus aureus ssp aureus DSM 20231T DSM	1.452	46170
3 (-)	Lactobacillus brevis DSM 1267 DSM	1.358	1580
4 (-)	Clostridium novyi A 1025_NCTC 538 BOG	1.353	1542
5 (-)	Arthrobacter mysorens DSM 12798T DSM	1.351	257984
6 (-)	Staphylococcus simiae DSM 17636T DSM	1.345	308354
7 (-)	Staphylococcus simiae DSM 17637 DSM	1.332	308354
8 (-)	Staphylococcus aureus ssp aureus DSM 799 DSM	1.314	46170
9 (-)	Mycoplasma hyorhinis FLR	1.3	2100
10 (-)	Actinocorallia libanotica B246 UFL	1.281	46162

Analyte79

Analyte Name:	D7
Analyte Description:	
Analyte ID:	27B
Analyte Creation Date/Time:	2019-12-04T13:54:32.710
Applied MSP Library(ies):	BDAL, Filamentous Fungi Library 1.0, Mycobacteria Library 1.0 (bead method), IVD, Listeria
Applied Taxonomy Tree:	

Rank (Quality)	Matched Pattern	Score Value	NCBI Identifier
1 (+)	Rothia mucilaginosa BK2995_09 ERL	1.768	43675
2 (+)	Rothia mucilaginosa CCUG 44966 CCUG	1.708	43675
3 (-)	Rothia mucilaginosa CCUG 52532 CCUG	1.627	43675
4 (-)	Rothia mucilaginosa DSM 20446 BRB	1.556	43675
5 (-)	Rothia dentocariosa CCUG 29965 CCUG	1.49	2047
6 (-)	Rothia mucilaginosa DSM 30548 DSM	1.363	43675
7 (-)	Streptococcus gordonii DSM 6777T DSM	1.291	1302
8 (-)	Lactobacillus acidophilus DSM 20079T DSM	1.267	1579
9 (-)	Staphylococcus capitis ssp capitis DSM 6180 DSM	1.266	72758
10 (-)	Arthrobacter oxydans IMET 10684T HKJ	1.262	1671

Analyte80

Analyte Name:	D8
Analyte Description:	
Analyte ID:	27B
Analyte Creation Date/Time:	2019-12-04T13:54:31.887
Applied MSP Library(ies):	BDAL, Filamentous Fungi Library 1.0, Mycobacteria Library 1.0 (bead method), IVD, Listeria
Applied Taxonomy Tree:	

Rank (Quality)	Matched Pattern	Score Value	NCBI Identifier
1 (++)	Rothia mucilaginosa BK2995_09 ERL	2.046	43675
2 (+)	Rothia mucilaginosa DSM 20445 DSM	1.938	43675
3 (+)	Rothia mucilaginosa CCUG 44966 CCUG	1.777	43675
4 (+)	Rothia mucilaginosa DSM 20446 BRB	1.709	43675
5 (-)	Rothia mucilaginosa CCUG 52532 CCUG	1.516	43675
6 (-)	Rothia mucilaginosa CCUG 31189 CCUG	1.51	43675
7 (-)	Rothia mucilaginosa DSM 30548 DSM	1.503	43675
8 (-)	Streptococcus gordonii DSM 6777T DSM	1.443	1302
9 (-)	Paenibacillus alvei DSM 5557 DSM	1.425	44250
10 (-)	Rothia dentocariosa CCUG 29965 CCUG	1.397	2047

Analyte81

Analyte Name:	D9
Analyte Description:	
Analyte ID:	30A
Analyte Creation Date/Time:	2019-12-04T13:54:31.533
Applied MSP Library(ies):	BDAL, Filamentous Fungi Library 1.0, Mycobacteria Library 1.0 (bead method), IVD, Listeria
Applied Taxonomy Tree:	

Rank (Quality)	Matched Pattern	Score Value	NCBI Identifier
1 (-)	Lactobacillus plantarum ssp argentoratensis DSM 16365T DSM	1.46	271881
2 (-)	Trichosporon cutaneum 120 PSB	1.403	5554
3 (-)	Aromatoleum aromaticum EbN1 MPB	1.358	12960
4 (-)	Lactobacillus plantarum DSM 12028 DSM	1.339	1590
5 (-)	Clostridium novyi 1082_ATCC 17861T BOG	1.334	1542
6 (-)	Staphylococcus lentus DSM 20352T DSM	1.327	42858
7 (-)	Lactobacillus plantarum DSM 13273 DSM	1.314	1590
8 (-)	Starkeya novella B351 UFL	1.312	921
9 (-)	Lactobacillus paraplantarum DSM 10667T DSM	1.312	60520
10 (-)	Lactobacillus paracasei ssp paracasei DSM 20207 DSM	1.294	47714

Analyte82

Analyte Name:	D10
Analyte Description:	
Analyte ID:	30A
Analyte Creation Date/Time:	2019-12-04T13:54:32.123
Applied MSP Library(ies):	BDAL, Filamentous Fungi Library 1.0, Mycobacteria Library 1.0 (bead method), IVD, Listeria
Applied Taxonomy Tree:	

Rank (Quality)	Matched Pattern	Score Value	NCBI Identifier
1 (-)	Clostridium bifermentans 2274_CCUG 35556 A BOG	1.457	1490
2 (-)	Weissella halotolerans DSM 20190T DSM	1.433	1615
3 (-)	Streptomyces lavendulae B264 UFL	1.386	1914
4 (-)	Microbacterium liquefaciens HKI 11374 HKJ	1.374	33918
5 (-)	Lodderomyces elongisporus CBS 2605T CBS	1.37	36914
6 (-)	Arthrobacter parietis DSM 16404T DSM	1.37	271434
7 (-)	Lactobacillus agilis DSM 20510 DSM	1.346	1601
8 (-)	Lactobacillus fructivorans DSM 20203T DSM	1.346	1614
9 (-)	Actinocorallia libanotica B246 UFL	1.344	46162
10 (-)	Acinetobacter junii DSM 14968 HAM	1.328	40215

Analyte83

Analyte Name:	D11
Analyte Description:	
Analyte ID:	30B
Analyte Creation Date/Time:	2019-12-04T13:54:31.870
Applied MSP Library(ies):	BDAL, Filamentous Fungi Library 1.0, Mycobacteria Library 1.0 (bead method), IVD, Listeria
Applied Taxonomy Tree:	

Rank (Quality)	Matched Pattern	Score Value	NCBI Identifier
1 (-)	Filifactor villosus 1051_NCTC 11220T BOG	1.509	29374
2 (-)	Agromyces rhizospherae HKI 302_DSM 14597T HKJ	1.472	88374
3 (-)	Agromyces salentinus HKI 320_DSM 16198T HKJ	1.44	269421
4 (-)	Geotrichum sp 116 PSB	1.424	43987
5 (-)	Staphylococcus haemolyticus Mb18803_2 CHB	1.423	1283
6 (-)	Staphylococcus haemolyticus 10024 CHB	1.419	1283
7 (-)	Staphylococcus pasteuri DSM 10657 DSM	1.417	45972
8 (-)	Staphylococcus epidermidis 4b_r ESL	1.395	1282
9 (-)	Staphylococcus capitis ssp capitis DSM 20325 DSM	1.39	72758
10 (-)	Staphylococcus capitis ssp capitis DSM 20326T DSM	1.389	72758

Analyte84

Analyte Name:	D12
Analyte Description:	
Analyte ID:	30B
Analyte Creation Date/Time:	2019-12-04T13:54:32.202
Applied MSP Library(ies):	BDAL, Filamentous Fungi Library 1.0, Mycobacteria Library 1.0 (bead method), IVD, Listeria
Applied Taxonomy Tree:	

Rank (Quality)	Matched Pattern	Score Value	NCBI Identifier
1 (-)	Arthrobacter ramosus IMET 10685T HKJ	1.482	1672
2 (-)	Candida lusitaniae CBS 4413T CBS	1.389	36911
3 (-)	Lactobacillus malefermentans DSM 20177 DSM	1.379	176292
4 (-)	Clostridium difficile MB_4499_05 THL	1.358	1496
5 (-)	Lactobacillus brevis DSM 2647 DSM	1.356	1580
6 (-)	Filifactor villosus 1051_NCTC 11220T BOG	1.35	29374
7 (-)	Staphylococcus haemolyticus 19 ESL	1.339	1283
8 (-)	Staphylococcus haemolyticus Mb18803_2 CHB	1.336	1283
9 (-)	Candida krusei ATCC 6258 THL	1.308	4909
10 (-)	Streptomyces violaceoruber B263 UFL	1.301	1935

Analyte85

Analyte Name:	D13
Analyte Description:	
Analyte ID:	31
Analyte Creation Date/Time:	2019-12-04T13:54:32.028
Applied MSP Library(ies):	BDAL, Filamentous Fungi Library 1.0, Mycobacteria Library 1.0 (bead method), IVD, Listeria
Applied Taxonomy Tree:	

Rank (Quality)	Matched Pattern	Score Value	NCBI Identifier
1 (++)	Rothia dentocariosa DSM 43762T DSM	2.047	2047
2 (+)	Rothia dentocariosa CCUG 29965 CCUG	1.983	2047
3 (+)	Rothia mucilaginosa CCUG 44966 CCUG	1.797	43675
4 (+)	Rothia mucilaginosa CCUG 52532 CCUG	1.787	43675
5 (+)	Rothia mucilaginosa DSM 20446 DSM	1.77	43675
6 (+)	Rothia mucilaginosa BK2995_09 ERL	1.752	43675
7 (-)	Rothia mucilaginosa DSM 20445 DSM	1.687	43675
8 (-)	Rothia mucilaginosa DSM 20446 BRB	1.603	43675
9 (-)	Rothia mucilaginosa CCUG 31189 CCUG	1.582	43675
10 (-)	Rothia dentocariosa RV_BA1_032010_D LBK	1.484	2047

Analyte86

Analyte Name:	D14
Analyte Description:	
Analyte ID:	31
Analyte Creation Date/Time:	2019-12-04T13:54:31.728
Applied MSP Library(ies):	BDAL, Filamentous Fungi Library 1.0, Mycobacteria Library 1.0 (bead method), IVD, Listeria
Applied Taxonomy Tree:	

Rank (Quality)	Matched Pattern	Score Value	NCBI Identifier
1 (++)	Rothia dentocariosa DSM 43762T DSM	2.12	2047
2 (+)	Rothia mucilaginosa DSM 20446 DSM	1.965	43675
3 (+)	Rothia mucilaginosa BK2995_09 ERL	1.834	43675
4 (+)	Rothia mucilaginosa CCUG 44966 CCUG	1.794	43675
5 (+)	Rothia mucilaginosa DSM 20445 DSM	1.721	43675
6 (-)	Rothia dentocariosa CCUG 29965 CCUG	1.586	2047
7 (-)	Rothia mucilaginosa CCUG 31189 CCUG	1.557	43675
8 (-)	Rothia mucilaginosa CCUG 52532 CCUG	1.476	43675
9 (-)	Rothia dentocariosa RV_BA1_032010_D LBK	1.4	2047
10 (-)	Burkholderia anthina LMG 16670 HAM	1.35	179879

Analyte87

Analyte Name:	D15
Analyte Description:	
Analyte ID:	32
Analyte Creation Date/Time:	2019-12-04T13:54:32.276
Applied MSP Library(ies):	BDAL, Filamentous Fungi Library 1.0, Mycobacteria Library 1.0 (bead method), IVD, Listeria
Applied Taxonomy Tree:	

Rank (Quality)	Matched Pattern	Score Value	NCBI Identifier
1 (-)	Filifactor villosus 1051_NCTC 11220T BOG	1.698	29374
2 (-)	Lactobacillus paracasei ssp paracasei DSM 20207 DSM	1.556	47714
3 (-)	Lactobacillus plantarum DSM 20205 DSM	1.465	1590
4 (-)	Clostridium bifermentans 2274_CCUG 35556 A BOG	1.392	1490
5 (-)	Candida tropicalis VML	1.388	5482
6 (-)	Aeromonas encheleia CECT 4342T DSM	1.376	73010
7 (-)	Aspergillus versicolor F68 RLH	1.348	123269315
8 (-)	Mycobacterium bovis BCG 1878 PGM	1.329	1765
9 (-)	Lactobacillus gastricus DSM 16046 DSM	1.306	227942
10 (-)	Aeromonas jandaei CECT 4228T DSM	1.305	650

Analyte88

Analyte Name:	D16
Analyte Description:	
Analyte ID:	32
Analyte Creation Date/Time:	2019-12-04T13:54:32.095
Applied MSP Library(ies):	BDAL, Filamentous Fungi Library 1.0, Mycobacteria Library 1.0 (bead method), IVD, Listeria
Applied Taxonomy Tree:	

Rank (Quality)	Matched Pattern	Score Value	NCBI Identifier
1 (-)	Arthrobacter ramosus IMET 10685T HKJ	1.455	1672
2 (-)	Nocardia pneumoniae DSM 44730T DSM	1.436	228601
3 (-)	Enterobacter cloacae 13159_1 CHB	1.417	550
4 (-)	Vibrio penaeicida DSM 14398T HAM	1.361	104609
5 (-)	Tsukamurella paurometabola DSM 43345 DSM	1.351	2061
6 (-)	Actinocorallia libanotica B246 UFL	1.35	46162
7 (-)	Clostridium bifermentans 2274_CCUG 35556 A BOG	1.34	1490
8 (-)	Colletotrichum gloeosporioides CBS 100471 CBS	1.327	474922
9 (-)	Rubrivivax gelatinosus DSMZ 1709T PAH	1.32	28068
10 (-)	Avibacterium avium DSM 18557T DSM	1.316	751

Analyte89

Analyte Name:	D17
Analyte Description:	
Analyte ID:	33A
Analyte Creation Date/Time:	2019-12-04T13:54:31.761
Applied MSP Library(ies):	BDAL, Filamentous Fungi Library 1.0, Mycobacteria Library 1.0 (bead method), IVD, Listeria
Applied Taxonomy Tree:	

Rank (Quality)	Matched Pattern	Score Value	NCBI Identifier
1 (++)	Rothia mucilaginosa CCUG 44966 CCUG	2.115	43675
2 (+)	Rothia dentocariosa CCUG 29965 CCUG	1.812	2047
3 (+)	Rothia mucilaginosa CCUG 31189 CCUG	1.809	43675
4 (+)	Rothia mucilaginosa DSM 20446 BRB	1.757	43675
5 (+)	Rothia mucilaginosa BK2995_09 ERL	1.736	43675
6 (-)	Rothia mucilaginosa DSM 30548 DSM	1.422	43675
7 (-)	Rothia mucilaginosa DSM 20445 DSM	1.403	43675
8 (-)	Rothia dentocariosa DSM 43762T DSM	1.395	2047
9 (-)	Arthrobacter stackebrandtii DSM 16005T DSM	1.359	272161
10 (-)	Sinomonas atrocyanea DSM 20127T DSM	1.343	37927

Analyte90

Analyte Name:	D18
Analyte Description:	
Analyte ID:	33A
Analyte Creation Date/Time:	2019-12-04T13:54:32.507
Applied MSP Library(ies):	BDAL, Filamentous Fungi Library 1.0, Mycobacteria Library 1.0 (bead method), IVD, Listeria
Applied Taxonomy Tree:	

Rank (Quality)	Matched Pattern	Score Value	NCBI Identifier
1 (++)	Rothia mucilaginosa BK2995_09 ERL	2.298	43675
2 (++)	Rothia mucilaginosa CCUG 31189 CCUG	2.044	43675
3 (++)	Rothia mucilaginosa CCUG 44966 CCUG	2.042	43675
4 (+)	Rothia mucilaginosa DSM 20445 DSM	1.98	43675
5 (+)	Rothia mucilaginosa DSM 20446 BRB	1.889	43675
6 (+)	Rothia mucilaginosa CCUG 52532 CCUG	1.856	43675
7 (-)	Rothia dentocariosa CCUG 29965 CCUG	1.628	2047
8 (-)	Rothia mucilaginosa DSM 30548 DSM	1.535	43675
9 (-)	Sinomonas atrocyanea DSM 20127T DSM	1.325	37927
10 (-)	Tatumella citrea DSM 13699T HAM	1.321	53336

Analyte91

Analyte Name:	D19
Analyte Description:	
Analyte ID:	33B
Analyte Creation Date/Time:	2019-12-04T13:54:32.584
Applied MSP Library(ies):	BDAL, Filamentous Fungi Library 1.0, Mycobacteria Library 1.0 (bead method), IVD, Listeria
Applied Taxonomy Tree:	

Rank (Quality)	Matched Pattern	Score Value	NCBI Identifier
1 (-)	Mycoplasma hyorhinis FLR	1.503	2100
2 (-)	Colletotrichum gloeosporioides CBS 100471 CBS	1.493	474922
3 (-)	Staphylococcus warneri CCM 2604 CCM	1.467	1292
4 (-)	Mycobacterium bovis BCG 1878 PGM	1.411	1765
5 (-)	Filifactor villosus 1051_NCTC 11220T BOG	1.395	29374
6 (-)	Agromyces salentinus HKI 320_DSM 16198T HKJ	1.391	269421
7 (-)	Lactobacillus bifermentans DSM 20003T DSM	1.378	1607
8 (-)	Lactobacillus fermentum DSM 20391 DSM	1.371	1613
9 (-)	Lactobacillus plantarum DSM 20205 DSM	1.361	1590
10 (-)	Bergeyella zoohelcum LMG 8351T HAM	1.345	1015

Analyte92

Analyte Name:	D20
Analyte Description:	
Analyte ID:	33B
Analyte Creation Date/Time:	2019-12-04T13:54:32.723
Applied MSP Library(ies):	BDAL, Filamentous Fungi Library 1.0, Mycobacteria Library 1.0 (bead method), IVD, Listeria
Applied Taxonomy Tree:	

Rank (Quality)	Matched Pattern	Score Value	NCBI Identifier
1 (-)	Mycoplasma hyorhinis FLR	1.646	2100
2 (-)	Agromyces rhizospherae HKI 302_DSM 14597T HKJ	1.624	88374
3 (-)	Streptomyces lavendulae B264 UFL	1.495	1914
4 (-)	Lactobacillus ingluviei DSM 15946T DSM	1.448	148604
5 (-)	Agromyces bracchium HKI 303 DSM 14596T HKJ	1.439	88376
6 (-)	Lactobacillus agilis DSM 20509T DSM	1.416	1601
7 (-)	Lactobacillus agilis DSM 20510 DSM	1.394	1601
8 (-)	Thauera aromatica strain XyN1 MPB	1.382	59405
9 (-)	Pichia occidentalis CBS 1910 CBS	1.377	54552
10 (-)	Vibrio alginolyticus CCM 5941 CCM	1.365	663

Analyte93

Analyte Name:	D21
Analyte Description:	
Analyte ID:	35A
Analyte Creation Date/Time:	2019-12-04T13:54:32.641
Applied MSP Library(ies):	BDAL, Filamentous Fungi Library 1.0, Mycobacteria Library 1.0 (bead method), IVD, Listeria
Applied Taxonomy Tree:	

Rank (Quality)	Matched Pattern	Score Value	NCBI Identifier
1 (+)	Staphylococcus aureus ssp aureus DSM 4910 DSM	1.705	46170
2 (-)	Arthrobacter ramosus IMET 10685T HKJ	1.474	1672
3 (-)	Staphylococcus aureus ATCC 33862 THL	1.435	1280
4 (-)	Staphylococcus aureus ssp aureus DSM 3463 DSM	1.434	46170
5 (-)	Clostridium difficile 1020_NCTC 11206 BOG	1.41	1496
6 (-)	Lactobacillus salivarius DSM 20554 DSM	1.37	1624
7 (-)	Staphylococcus aureus ATCC 33591 THL	1.368	1280
8 (-)	Staphylococcus aureus ssp aureus DSM 20231T DSM	1.357	46170
9 (-)	Staphylococcus aureus ATCC 29213 THL	1.352	1280
10 (-)	Staphylococcus aureus ssp aureus DSM 346 DSM	1.349	46170

Analyte94

Analyte Name:	D22
Analyte Description:	
Analyte ID:	35A
Analyte Creation Date/Time:	2019-12-04T13:54:31.551
Applied MSP Library(ies):	BDAL, Filamentous Fungi Library 1.0, Mycobacteria Library 1.0 (bead method), IVD, Listeria
Applied Taxonomy Tree:	

Rank (Quality)	Matched Pattern	Score Value	NCBI Identifier
1 (-)	Janthinobacterium lividum CIP 106720T HAM	1.49	29581
2 (-)	Pichia occidentalis CBS 1910 CBS	1.448	54552
3 (-)	Rhizobium rubi DSM 6772T HAM	1.438	28099
4 (-)	Rhizobium radiobacter B166 UFL	1.396	358
5 (-)	Clostridium cochlearium 1080_ATCC 17794T BOG	1.372	1494
6 (-)	Lactobacillus salivarius DSM 20554 DSM	1.36	1624
7 (-)	Staphylococcus aureus ssp aureus DSM 4910 DSM	1.36	46170
8 (-)	Pseudomonas marginalis DSM 13124T HAM	1.334	298
9 (-)	Balneatrix alpica CIP 103589T HAM	1.329	75684
10 (-)	Arthrobacter oxydans DSM 20119T DSM	1.326	1671

Analyte95

Analyte Name:	D23
Analyte Description:	
Analyte ID:	35B
Analyte Creation Date/Time:	2019-12-04T13:54:31.659
Applied MSP Library(ies):	BDAL, Filamentous Fungi Library 1.0, Mycobacteria Library 1.0 (bead method), IVD, Listeria
Applied Taxonomy Tree:	

Rank (Quality)	Matched Pattern	Score Value	NCBI Identifier
1 (-)	Lactobacillus satsumensis DSM 16230T DSM	1.478	259059
2 (-)	Streptomyces avidinii B190 UFL	1.429	1895
3 (-)	Vibrio vulnificus CCM 2840 CCM	1.427	672
4 (-)	Filifactor villosus 1051_NCTC 11220T BOG	1.42	29374
5 (-)	Mycobacterium bovis Bovinus An_1 PGM	1.41	1765
6 (-)	Clostridium bifermentans 2274_CCUG 35556 A BOG	1.397	1490
7 (-)	Streptomyces lavendulae B264 UFL	1.356	1914
8 (-)	Thauera phenylacetica B4P MPB	1.316	164400
9 (-)	Colletotrichum gloeosporioides CBS 100471 CBS	1.315	474922
10 (-)	Staphylococcus equorum ssp equorum DSM 20674T DSM	1.307	29383

Analyte96

Analyte Name:	D24
Analyte Description:	
Analyte ID:	35B
Analyte Creation Date/Time:	2019-12-04T13:54:31.982
Applied MSP Library(ies):	Listeria, IVD, Mycobacteria Library 1.0 (bead method), Filamentous Fungi Library 1.0, BDAL
Applied Taxonomy Tree:	

Rank (Quality)	Matched Pattern	Score Value	NCBI Identifier
1 (-)	Candida lambica CBS 603 CBS	1.616	53655
2 (-)	Streptomyces lavendulae B264 UFL	1.484	1914
3 (-)	Arthrobacter ramosus IMET 10685T HKJ	1.421	1672
4 (-)	Cryptococcus neoformans ATCC 14116 THL	1.411	5207
5 (-)	Colletotrichum gloeosporioides CBS 100471 CBS	1.403	474922
6 (-)	Candida krusei ATCC 6258 THL	1.4	4909
7 (-)	Lactobacillus coryniformis ssp torquens DSM 20004T DSM	1.355	115542
8 (-)	Streptomyces griseus B261 UFL	1.347	1911
9 (-)	Mycobacterium chelonae ssp chelonae DSM 43217 DSM b	1.33	122893407
10 (-)	Agromyces rhizospherae HKI 302_DSM 14597T HKJ	1.33	88374

Analyte97

Analyte Name:	E1
Analyte Description:	
Analyte ID:	37A
Analyte Creation Date/Time:	2019-12-04T13:54:32.540
Applied MSP Library(ies):	BDAL, Filamentous Fungi Library 1.0, Mycobacteria Library 1.0 (bead method), IVD, Listeria
Applied Taxonomy Tree:	

Rank (Quality)	Matched Pattern	Score Value	NCBI Identifier
1 (-)	Rothia mucilaginosa CCUG 52532 CCUG	1.516	43675
2 (-)	Arthrobacter stackebrandtii DSM 16005T DSM	1.383	272161
3 (-)	Paenibacillus sp DSM 1487 DSM	1.306	44249
4 (-)	Rothia mucilaginosa DSM 20445 DSM	1.305	43675
5 (-)	Lactobacillus saerimneri DSM 16049T DSM	1.293	228229
6 (-)	Rothia dentocariosa CCUG 29965 CCUG	1.287	2047
7 (-)	Corynebacterium confusum DSM 44384T DSM	1.272	71254
8 (-)	Weissella viridescens DSM 20248 DSM	1.258	1629
9 (-)	Burkholderia cepacia MB_7544_05 THL	1.244	292
10 (-)	Brevibacillus agri DSM 6348T DSM	1.227	51101

Analyte98

Analyte Name:	E2
Analyte Description:	
Analyte ID:	37A
Analyte Creation Date/Time:	2019-12-04T13:54:31.547
Applied MSP Library(ies):	BDAL, Filamentous Fungi Library 1.0, Mycobacteria Library 1.0 (bead method), IVD, Listeria
Applied Taxonomy Tree:	

Rank (Quality)	Matched Pattern	Score Value	NCBI Identifier
1 (-)	Paenibacillus taiwanensis DSM 18679T DSM	1.393	401638
2 (-)	Paenibacillus gansuensis DSM 16968T DSM	1.317	44249
3 (-)	Paenibacillus alvei DSM 5560 DSM	1.307	44250
4 (-)	Rothia dentocariosa RV_BA1_032010_D LBK	1.306	2047
5 (-)	Ralstonia pickettii 21323_1 CHB	1.263	329
6 (-)	Paenibacillus mendelii DSM 19248T DSM	1.261	206163
7 (-)	Acinetobacter haemolyticus LMG 1033 HAM	1.26	29430
8 (-)	Thauera aromatica strain XyN1 MPB	1.258	59405
9 (-)	Weissella viridescens DSM 20248 DSM	1.249	1629
10 (-)	Paenibacillus glucanolyticus DSM 5162T DSM	1.233	59843

Analyte99

Analyte Name:	E3
Analyte Description:	
Analyte ID:	37B
Analyte Creation Date/Time:	2019-12-04T13:54:31.851
Applied MSP Library(ies):	BDAL, Filamentous Fungi Library 1.0, Mycobacteria Library 1.0 (bead method), IVD, Listeria
Applied Taxonomy Tree:	

Rank (Quality)	Matched Pattern	Score Value	NCBI Identifier
1 (-)	Streptococcus salivarius 0807M25049501 IBS	1.528	1304
2 (-)	Streptococcus salivarius ssp salivarius 140417_01 ETL	1.48	1304
3 (-)	Terrimonas ferruginea DSM 30193T HAM	1.462	249
4 (-)	Streptococcus macacae DSM 20724T DSM	1.381	1339
5 (-)	Lactobacillus curvatus DSM 20496 DSM	1.352	28038
6 (-)	Lactobacillus sharpeae DSM 20504 DSM	1.347	1626
7 (-)	Lactobacillus paracasei ssp paracasei DSM 8741 DSM	1.339	47714
8 (-)	Dermatophilus congolensis DSM 44172 DSM	1.31	1863
9 (-)	Streptococcus sanguinis DSM 20567T DSM	1.304	1305
10 (-)	Azoarcus indigens VB32 MPB	1.302	29545

Analyte100

Analyte Name:	E4
Analyte Description:	
Analyte ID:	37B
Analyte Creation Date/Time:	2019-12-04T13:54:32.668
Applied MSP Library(ies):	BDAL, Filamentous Fungi Library 1.0, Mycobacteria Library 1.0 (bead method), IVD, Listeria
Applied Taxonomy Tree:	

Rank (Quality)	Matched Pattern	Score Value	NCBI Identifier
1 (-)	Lactobacillus antri DSM 16041T DSM	1.466	227943
2 (-)	Aeromonas salmonicida ssp salmonicida CECT 894T DSM	1.428	29491
3 (-)	Mycoplasma hyorhinis FLR	1.372	2100
4 (-)	Pseudomonas mucidolens LMG 2223T HAM	1.357	46679
5 (-)	Streptomyces griseus B261 UFL	1.356	1911
6 (-)	Malassezia furfur DSM 6170 DSM	1.32	55194
7 (-)	Dermatophilus congolensis DSM 44174 DSM	1.315	1863
8 (-)	Rhodococcus gordoniae DSM 44690 DSM	1.311	223392
9 (-)	Agromyces rhizospherae HKI 302_DSM 14597T HKJ	1.31	88374
10 (-)	Streptococcus minor DSM 17118T DSM	1.308	229549

Analyte101

Analyte Name:	E5
Analyte Description:	
Analyte ID:	37C
Analyte Creation Date/Time:	2019-12-04T13:54:32.699
Applied MSP Library(ies):	BDAL, Filamentous Fungi Library 1.0, Mycobacteria Library 1.0 (bead method), IVD, Listeria
Applied Taxonomy Tree:	

Rank (Quality)	Matched Pattern	Score Value	NCBI Identifier
1 (-)	Weissella viridescens DSM 20248 DSM	1.415	1629
2 (-)	Rothia mucilaginosa DSM 20445 DSM	1.392	43675
3 (-)	Paenibacillus xinjiangensis DSM 16970T DSM	1.362	459527
4 (-)	Paenibacillus glucanolyticus DSM 5162T DSM	1.323	59843
5 (-)	Paenibacillus assamensis DSM 18201T DSM	1.307	311244
6 (-)	Rothia aeria CCUG 50760 CCUG	1.305	172042
7 (-)	Cellulosimicrobium cellulans B480 UFL	1.29	1710
8 (-)	Rothia aeria DSM 14556T DSM	1.273	172042
9 (-)	Arthrobacter nasiphocae DSM 13988T DSM	1.269	189863
10 (-)	Rothia aeria 120619_15_b HUA	1.268	172042

Analyte102

Analyte Name:	E6
Analyte Description:	
Analyte ID:	37C
Analyte Creation Date/Time:	2019-12-04T13:54:31.694
Applied MSP Library(ies):	BDAL, Filamentous Fungi Library 1.0, Mycobacteria Library 1.0 (bead method), IVD, Listeria
Applied Taxonomy Tree:	

Rank (Quality)	Matched Pattern	Score Value	NCBI Identifier
1 (-)	Paenibacillus glucanolyticus DSM 5162T DSM	1.456	59843
2 (-)	Paenibacillus jamilae DSM 13815T DSM	1.374	114136
3 (-)	Aromatoleum aromaticum EbN1 MPB	1.342	12960
4 (-)	Halomonas elongata B558 UFL	1.308	2746
5 (-)	Ochrobactrum anthropi DSM 20150 DSM	1.281	529
6 (-)	Arthrobacter ramosus IMET 10685T HKJ	1.276	1672
7 (-)	Cupriavidus necator DSM 531 HAM	1.275	106590
8 (-)	Weissella viridescens DSM 20248 DSM	1.266	1629
9 (-)	Pseudomonas nitroreducens LMG 20221T HAM	1.266	46680
10 (-)	Ochrobactrum grignonense DSM 13338T HAM	1.264	94627

Analyte103

Analyte Name:	E7
Analyte Description:	
Analyte ID:	39A
Analyte Creation Date/Time:	2019-12-04T13:54:31.627
Applied MSP Library(ies):	BDAL, Filamentous Fungi Library 1.0, Mycobacteria Library 1.0 (bead method), IVD, Listeria
Applied Taxonomy Tree:	

Rank (Quality)	Matched Pattern	Score Value	NCBI Identifier
1 (-)	Raoultella planticola DSM 3069T DSM	1.363	575
2 (-)	Arthrobacter ramosus IMET 10685T HKJ	1.362	1672
3 (-)	Paracoccus versutus B352 UFL	1.333	34007
4 (-)	Pichia occidentalis CBS 1910 CBS	1.311	54552
5 (-)	Clostridium chauvoei 1023_NCTC 8070 BOG	1.307	46867
6 (-)	Candida guilliermondii CBS 566 CBS	1.298	4929
7 (-)	Pseudomonas syringae ssp syringae LMG 1247T HAM	1.298	317
8 (-)	Actinomyces funkei DSM 15537T DSM	1.28	132933
9 (-)	Candida lambica CBS 603 CBS	1.279	53655
10 (-)	Clostridium baratii 1084_ATCC 25782 BOG	1.262	1561

Analyte104

Analyte Name:	E8
Analyte Description:	
Analyte ID:	39A
Analyte Creation Date/Time:	2019-12-04T13:54:32.159
Applied MSP Library(ies):	BDAL, Filamentous Fungi Library 1.0, Mycobacteria Library 1.0 (bead method), IVD, Listeria
Applied Taxonomy Tree:	

Rank (Quality)	Matched Pattern	Score Value	NCBI Identifier
1 (-)	Streptococcus salivarius DSM 20560T DSM	1.549	1304
2 (-)	Candida lambica CBS 603 CBS	1.509	53655
3 (-)	Streptococcus salivarius IBS_MS_23 IBS	1.5	1304
4 (-)	Lactobacillus brevis DSM 1268 DSM	1.436	1580
5 (-)	Lactobacillus sharpeae DSM 20505T DSM	1.428	1626
6 (-)	Lactobacillus aviarius ssp aviarius DSM 20654 DSM	1.398	147810
7 (-)	Lactobacillus sharpeae DSM 20504 DSM	1.381	1626
8 (-)	Lactobacillus plantarum DSM 20205 DSM	1.336	1590
9 (-)	Streptomyces lavendulae B264 UFL	1.332	1914
10 (-)	Lactobacillus plantarum ssp argentoratensis DSM 16365T DSM	1.322	271881

Analyte105

Analyte Name:	E9
Analyte Description:	
Analyte ID:	39B
Analyte Creation Date/Time:	2019-12-04T13:54:31.927
Applied MSP Library(ies):	BDAL, Filamentous Fungi Library 1.0, Mycobacteria Library 1.0 (bead method), IVD, Listeria
Applied Taxonomy Tree:	

Rank (Quality)	Matched Pattern	Score Value	NCBI Identifier
1 (-)	Clostridium bifermentans 2274_CCUG 35556 A BOG	1.48	1490
2 (-)	Filifactor villosus 1051_NCTC 11220T BOG	1.423	29374
3 (-)	Sphingomonas sp B605 UFL	1.335	13687
4 (-)	Aspergillus flavus 1081 PFM	1.314	5059
5 (-)	Pichia occidentalis CBS 1910 CBS	1.294	54552
6 (-)	Staphylococcus simulans DSM 20324 DSM	1.291	1286
7 (-)	Sinomonas atrocyanea DSM 20127T DSM	1.281	37927
8 (-)	Lactobacillus murinus DSM 20452T DSM	1.28	1622
9 (-)	Pseudomonas syringae ssp syringae LMG 1247T HAM	1.279	317
10 (-)	Lodderomyces elongisporus CBS 2605T CBS	1.276	36914

Analyte106

Analyte Name:	E10
Analyte Description:	
Analyte ID:	39B
Analyte Creation Date/Time:	2019-12-04T13:54:32.491
Applied MSP Library(ies):	BDAL, Filamentous Fungi Library 1.0, Mycobacteria Library 1.0 (bead method), IVD, Listeria
Applied Taxonomy Tree:	

Rank (Quality)	Matched Pattern	Score Value	NCBI Identifier
1 (-)	Actinomyces suimastitidis DSM 15538T DSM	1.537	121163
2 (-)	Lactobacillus satsumensis DSM 16230T DSM	1.519	259059
3 (-)	Candida lusitaniae CBS 4413T CBS	1.478	36911
4 (-)	Arthrobacter ramosus IMET 10685T HKJ	1.434	1672
5 (-)	Filifactor villosus 1051_NCTC 11220T BOG	1.372	29374
6 (-)	Lodderomyces elongisporus CBS 2605T CBS	1.369	36914
7 (-)	Lactobacillus equi DSM 15833T DSM	1.34	137357
8 (-)	Lactobacillus paracasei ssp paracasei DSM 20207 DSM	1.328	47714
9 (-)	Staphylococcus simulans DSM 20723 DSM	1.321	1286
10 (-)	Lactobacillus paracasei ssp paracasei DSM 5622T DSM	1.321	47714

Analyte107

Analyte Name:	E11
Analyte Description:	
Analyte ID:	39C
Analyte Creation Date/Time:	2019-12-04T13:54:32.727
Applied MSP Library(ies):	BDAL, Filamentous Fungi Library 1.0, Mycobacteria Library 1.0 (bead method), IVD, Listeria
Applied Taxonomy Tree:	

Rank (Quality)	Matched Pattern	Score Value	NCBI Identifier
1 (-)	Candida lambica CBS 603 CBS	1.605	53655
2 (-)	Filifactor villosus 1051_NCTC 11220T BOG	1.595	29374
3 (-)	Clostridium novyi 1082_ATCC 17861T BOG	1.57	1542
4 (-)	Cryptococcus neoformans ATCC 14116 THL	1.512	5207
5 (-)	Agromyces fucosus HKI 11529_DSM 8597T HKJ	1.492	41985
6 (-)	Pichia occidentalis CBS 1910 CBS	1.433	54552
7 (-)	Clostridium difficile MB_7869_05 THL	1.397	1496
8 (-)	Magnusiomyces capitatus CBS 571_82 CBS	1.393	43960
9 (-)	Agromyces rhizospherae HKI 302_DSM 14597T HKJ	1.386	88374
10 (-)	Arthrobacter ramosus IMET 10685T HKJ	1.335	1672

Analyte108

Analyte Name:	E12
Analyte Description:	
Analyte ID:	39C
Analyte Creation Date/Time:	2019-12-04T13:54:31.601
Applied MSP Library(ies):	BDAL, Filamentous Fungi Library 1.0, Mycobacteria Library 1.0 (bead method), IVD, Listeria
Applied Taxonomy Tree:	

Rank (Quality)	Matched Pattern	Score Value	NCBI Identifier
1 (-)	Cryptococcus neoformans ATCC 14116 THL	1.501	5207
2 (-)	Clostridium bifermentans 2274_CCUG 35556 A BOG	1.427	1490
3 (-)	Arthrobacter ramosus IMET 10685T HKJ	1.426	1672
4 (-)	Streptomyces albus B262 UFL	1.419	1888
5 (-)	Pichia occidentalis CBS 1910 CBS	1.405	54552
6 (-)	Clostridium difficile MB_7869_05 THL	1.403	1496
7 (-)	Mycobacterium szulgai DSM 44166T DSM	1.395	1787
8 (-)	Clostridium novyi A 1025_NCTC 538 BOG	1.38	1542
9 (-)	Staphylococcus epidermidis ATCC 12228 CHB	1.374	1282
10 (-)	Clostridium novyi 1082_ATCC 17861T BOG	1.333	1542

Analyte109

Analyte Name:	E13
Analyte Description:	
Analyte ID:	40A
Analyte Creation Date/Time:	2019-12-04T13:54:32.745
Applied MSP Library(ies):	BDAL, Filamentous Fungi Library 1.0, Mycobacteria Library 1.0 (bead method), IVD, Listeria
Applied Taxonomy Tree:	

Rank (Quality)	Matched Pattern	Score Value	NCBI Identifier
1 (++)	Rothia dentocariosa DSM 43762T DSM	2.094	2047
2 (+)	Rothia mucilaginosa DSM 20746T DSM	1.942	43675
3 (+)	Rothia mucilaginosa BK2995_09 ERL	1.794	43675
4 (+)	Rothia dentocariosa CCUG 29965 CCUG	1.763	2047
5 (+)	Rothia mucilaginosa CCUG 44966 CCUG	1.744	43675
6 (-)	Rothia mucilaginosa CCUG 52532 CCUG	1.69	43675
7 (-)	Rothia mucilaginosa DSM 20446 DSM	1.681	43675
8 (-)	Rothia mucilaginosa DSM 20445 DSM	1.623	43675
9 (-)	Rothia aeria DSM 14556T DSM	1.53	172042
10 (-)	Arthrobacter stackebrandtii DSM 16005T DSM	1.421	272161

Analyte110

Analyte Name:	E14
Analyte Description:	
Analyte ID:	40A
Analyte Creation Date/Time:	2019-12-04T13:54:32.219
Applied MSP Library(ies):	Listeria, BDAL, Filamentous Fungi Library 1.0, Mycobacteria Library 1.0 (bead method), IVD
Applied Taxonomy Tree:	

Rank (Quality)	Matched Pattern	Score Value	NCBI Identifier
1 (++)	Rothia dentocariosa DSM 43762T DSM	2.022	2047
2 (-)	Rothia mucilaginosa DSM 20445 DSM	1.571	43675
3 (-)	Rothia mucilaginosa CCUG 52532 CCUG	1.398	43675
4 (-)	Rothia dentocariosa CCUG 29965 CCUG	1.337	2047
5 (-)	Rothia mucilaginosa DSM 20746T DSM	1.325	43675
6 (-)	Paenibacillus sp DSM 1487 DSM	1.31	44249
7 (-)	Lactobacillus plantarum DSM 13273 DSM	1.304	1590
8 (-)	Rothia mucilaginosa DSM 20446 DSM	1.304	43675
9 (-)	Rothia mucilaginosa BK2995_09 ERL	1.297	43675
10 (-)	Rothia mucilaginosa CCUG 31189 CCUG	1.257	43675

Analyte111

Analyte Name:	E15
Analyte Description:	
Analyte ID:	40B
Analyte Creation Date/Time:	2019-12-04T13:54:32.330
Applied MSP Library(ies):	BDAL, Filamentous Fungi Library 1.0, Mycobacteria Library 1.0 (bead method), IVD, Listeria
Applied Taxonomy Tree:	

Rank (Quality)	Matched Pattern	Score Value	NCBI Identifier
1 (-)	Sinomonas atrocyanea DSM 20127T DSM	1.5	37927
2 (-)	Filifactor villosus 1051_NCTC 11220T BOG	1.426	29374
3 (-)	Clostridium baratii 1018_NCTC 10986 BOG	1.41	1561
4 (-)	Microbacterium saperdae IMET 11076T HKJ	1.375	69368
5 (-)	Ralstonia sp B484 UFL	1.335	48736
6 (-)	Staphylococcus warneri CCM 2604 CCM	1.311	1292
7 (-)	Mycoplasma hyorhinis FLR	1.302	2100
8 (-)	Clostridium novyi 1082_ATCC 17861T BOG	1.3	1542
9 (-)	Agromyces rhizospherae HKI 302_DSM 14597T HKJ	1.286	88374
10 (-)	Clostridium haemolyticum 1069_ATCC 9650T BOG	1.265	84025

Analyte112

Analyte Name:	E16
Analyte Description:	
Analyte ID:	40B
Analyte Creation Date/Time:	2019-12-04T13:54:32.374
Applied MSP Library(ies):	BDAL, Filamentous Fungi Library 1.0, Mycobacteria Library 1.0 (bead method), IVD, Listeria
Applied Taxonomy Tree:	

Rank (Quality)	Matched Pattern	Score Value	NCBI Identifier
1 (-)	Pichia occidentalis CBS 1910 CBS	1.475	54552
2 (-)	Streptomyces lavendulae B264 UFL	1.425	1914
3 (-)	Filifactor villosus 1051_NCTC 11220T BOG	1.37	29374
4 (-)	Kingella kingae CCM 5679T CCM	1.362	504
5 (-)	Streptococcus salivarius DSM 20067 DSM	1.318	1304
6 (-)	Lactobacillus satsumensis DSM 16230T DSM	1.315	259059
7 (-)	Lactobacillus paracasei ssp paracasei DSM 20207 DSM	1.301	47714
8 (-)	Nocardia cyriacigeorgica DSM 44484T DSM	1.297	135487
9 (-)	Azoarcus communis Swub3 MPB	1.296	41977
10 (-)	Weissella minor DSM 20014T DSM	1.296	1620

Analyte113

Analyte Name:	E17
Analyte Description:	
Analyte ID:	40C
Analyte Creation Date/Time:	2019-12-04T13:54:32.051
Applied MSP Library(ies):	BDAL, Filamentous Fungi Library 1.0, Mycobacteria Library 1.0 (bead method), IVD, Listeria
Applied Taxonomy Tree:	

Rank (Quality)	Matched Pattern	Score Value	NCBI Identifier
1 (++)	Arthrobacter gandavensis DSM 15046T DSM	2.223	169960
2 (-)	Arthrobacter koreensis DSM 16760T DSM	1.556	199136
3 (-)	Arthrobacter tumbae DSM 16406T DSM	1.422	163874
4 (-)	Clostridium tetani type 1 1049_NCTC 279T BOG	1.384	1513
5 (-)	Burkholderia xenovorans LMG 21463T HAM	1.376	36873
6 (-)	Chryseobacterium chaponense DSM 23144 DSM	1.342	59732
7 (-)	Rhizobium tropici DSM 11418T HAM	1.316	398
8 (-)	Arthrobacter woluwensis DSM 10495T DSM	1.305	156980
9 (-)	Burkholderia phymatum LMG 21445T HAM	1.303	148447
10 (-)	Arthrobacter uratoxydans HKI 11492 HKJ	1.297	43667

Analyte114

Analyte Name:	E18
Analyte Description:	
Analyte ID:	40C
Analyte Creation Date/Time:	2019-12-04T13:54:31.766
Applied MSP Library(ies):	Listeria, IVD, Mycobacteria Library 1.0 (bead method), Filamentous Fungi Library 1.0, BDAL
Applied Taxonomy Tree:	

Rank (Quality)	Matched Pattern	Score Value	NCBI Identifier
1 (++)	Arthrobacter gandavensis DSM 15046T DSM	2.193	169960
2 (-)	Arthrobacter nicotinovorans DSM 420T DSM	1.568	29320
3 (-)	Arthrobacter koreensis DSM 16760T DSM	1.539	199136
4 (-)	Yersinia enterocolitica ssp enterocolitica ATCC 9610T THL	1.431	630
5 (-)	Arthrobacter woluwensis DSM 10495T DSM	1.403	156980
6 (-)	Comamonas terrigena DSM 7099T HAM	1.342	32013
7 (-)	Arthrobacter tumbae DSM 16406T DSM	1.297	163874
8 (-)	Arthrobacter polychromogenes DSM 20136T DSM	1.29	1676
9 (-)	Burkholderia caribensis DSM 13236T HAM	1.285	75105
10 (-)	Bacillus cereus DSM 31T DSM	1.265	1396

Analyte115

Analyte Name:	E19
Analyte Description:	
Analyte ID:	CN19A
Analyte Creation Date/Time:	2019-12-04T13:54:31.595
Applied MSP Library(ies):	BDAL, Filamentous Fungi Library 1.0, Mycobacteria Library 1.0 (bead method), IVD, Listeria
Applied Taxonomy Tree:	

Rank (Quality)	Matched Pattern	Score Value	NCBI Identifier
1 (+)	Rothia dentocariosa DSM 43762T DSM	1.723	2047
2 (-)	Arthrobacter stackebrandtii DSM 16005T DSM	1.498	272161
3 (-)	Rothia dentocariosa CCUG 29965 CCUG	1.325	2047
4 (-)	Rothia mucilaginosa DSM 20445 DSM	1.315	43675
5 (-)	Cellulosimicrobium cellulans B480 UFL	1.312	1710
6 (-)	Clostridium tetani 1089_ATCC 10779 BOG	1.229	1513
7 (-)	Actinomyces radingae 343 RLT	1.221	131110
8 (-)	Atopobium parvulum P10flue_re3AN USH	1.22	1382
9 (-)	Burkholderia pyrrocinia LMG 14191T HAM	1.197	60550
10 (-)	Streptococcus gallolyticus ssp gallolyticus DSM 16831T DSM	1.197	53354

Analyte116

Analyte Name:	E20
Analyte Description:	
Analyte ID:	CN19A
Analyte Creation Date/Time:	2019-12-04T13:54:32.139
Applied MSP Library(ies):	BDAL, Filamentous Fungi Library 1.0, Mycobacteria Library 1.0 (bead method), IVD, Listeria
Applied Taxonomy Tree:	

Rank (Quality)	Matched Pattern	Score Value	NCBI Identifier
1 (+)	Rothia dentocariosa DSM 43762T DSM	1.717	2047
2 (-)	Rothia dentocariosa CCUG 29965 CCUG	1.671	2047
3 (-)	Rothia mucilaginosa DSM 20746T DSM	1.459	43675
4 (-)	Rothia dentocariosa RV_BA1_032010_D LBK	1.456	2047
5 (-)	Rothia mucilaginosa CCUG 52532 CCUG	1.378	43675
6 (-)	Rothia mucilaginosa BK2995_09 ERL	1.346	43675
7 (-)	Arthrobacter stackebrandtii DSM 16005T DSM	1.259	272161
8 (-)	Brevibacillus borstelensis DSM 6347T DSM	1.243	45462
9 (-)	Rothia aeria DSM 14556T DSM	1.23	172042
10 (-)	Lactobacillus acidophilus DSM 20079T DSM	1.215	1579

Analyte117

Analyte Name:	E21
Analyte Description:	
Analyte ID:	CN19B
Analyte Creation Date/Time:	2019-12-04T13:54:32.737
Applied MSP Library(ies):	BDAL, Filamentous Fungi Library 1.0, Mycobacteria Library 1.0 (bead method), IVD, Listeria
Applied Taxonomy Tree:	

Rank (Quality)	Matched Pattern	Score Value	NCBI Identifier
1 (+)	Streptococcus salivarius 0807M25049501 IBS	1.825	1304
2 (-)	Streptococcus vestibularis CCUG 51352 CCUG	1.549	1343
3 (-)	Streptococcus macacae DSM 20724T DSM	1.516	1339
4 (-)	Streptococcus vestibularis CCUG 61229 CCUG	1.501	1343
5 (-)	Streptococcus orisuis DSM 18307T DSM	1.385	282078
6 (-)	Streptococcus salivarius DSM 20560T BRB	1.384	1304
7 (-)	Lactobacillus aviarius ssp aviarius DSM 20654 DSM	1.373	147810
8 (-)	Thauera phenylacetica B4P MPB	1.37	164400
9 (-)	Streptococcus salivarius DSM 20560T DSM	1.333	1304
10 (-)	Streptococcus sobrinus DSM 20742T DSM	1.33	1310

Analyte118

Analyte Name:	E22
Analyte Description:	
Analyte ID:	CN19B
Analyte Creation Date/Time:	2019-12-04T13:54:32.070
Applied MSP Library(ies):	BDAL, Filamentous Fungi Library 1.0, Mycobacteria Library 1.0 (bead method), IVD, Listeria
Applied Taxonomy Tree:	

Rank (Quality)	Matched Pattern	Score Value	NCBI Identifier
1 (+)	Streptococcus salivarius 0807M25049501 IBS	1.911	1304
2 (-)	Rothia dentocariosa G6496_ch28 IBS	1.635	2047
3 (-)	Streptococcus salivarius DSM 20560T DSM	1.612	1304
4 (-)	Rothia dentocariosa RV_BA1_032010_D LBK	1.536	2047
5 (-)	Streptococcus salivarius IBS_MS_23 IBS	1.494	1304
6 (-)	Streptococcus macacae DSM 20724T DSM	1.462	1339
7 (-)	Rothia dentocariosa CCUG 29965 CCUG	1.422	2047
8 (-)	Streptococcus vestibularis CCUG 61229 CCUG	1.406	1343
9 (-)	Lactobacillus aviarius ssp aviarius DSM 20654 DSM	1.378	147810
10 (-)	Lactobacillus plantarum DSM 13273 DSM	1.368	1590

Analyte119

Analyte Name:	E23
Analyte Description:	
Analyte ID:	C30A
Analyte Creation Date/Time:	2019-12-04T13:54:32.090
Applied MSP Library(ies):	BDAL, Filamentous Fungi Library 1.0, Mycobacteria Library 1.0 (bead method), IVD, Listeria
Applied Taxonomy Tree:	

Rank (Quality)	Matched Pattern	Score Value	NCBI Identifier
1 (+)	Rothia aeria NO_11 HUA	1.987	172042
2 (+)	Rothia aeria DSM 14556T DSM	1.832	172042
3 (+)	Rothia aeria 120619_15_b HUA	1.749	172042
4 (-)	Arthrobacter psychrolactophilus DSM 15612T DSM	1.572	92442
5 (-)	Rothia aeria CCUG 50760 CCUG	1.549	172042
6 (-)	Rothia dentocariosa CCUG 29965 CCUG	1.483	2047
7 (-)	Rothia dentocariosa DSM 43762T DSM	1.345	2047
8 (-)	Klebsiella oxytoca ATCC 700324 THL	1.32	571
9 (-)	Weissella viridescens DSM 20248 DSM	1.316	1629
10 (-)	Rothia dentocariosa G18709 IBS	1.289	2047

Analyte120

Analyte Name:	E24
Analyte Description:	
Analyte ID:	C30A
Analyte Creation Date/Time:	2019-12-04T13:54:32.685
Applied MSP Library(ies):	BDAL, Filamentous Fungi Library 1.0, Mycobacteria Library 1.0 (bead method), IVD, Listeria
Applied Taxonomy Tree:	

Rank (Quality)	Matched Pattern	Score Value	NCBI Identifier
1 (+)	Rothia aeria 120619_15_b HUA	1.7	172042
2 (-)	Rothia aeria DSM 14556T DSM	1.506	172042
3 (-)	Paenibacillus massiliensis DSM 16942T DSM	1.453	225917
4 (-)	Rothia aeria CCUG 50760 CCUG	1.448	172042
5 (-)	Streptococcus salivarius 0807M25049501 IBS	1.441	1304
6 (-)	Rothia aeria NO_11 HUA	1.421	172042
7 (-)	Arthrobacter psychrolactophilus DSM 15612T DSM	1.4	92442
8 (-)	Rothia dentocariosa CCUG 29965 CCUG	1.382	2047
9 (-)	Arthrobacter stackebrandtii DSM 16005T DSM	1.361	272161
10 (-)	Weissella viridescens DSM 20248 DSM	1.333	1629

Analyte121

Analyte Name:	F1
Analyte Description:	
Analyte ID:	C30B
Analyte Creation Date/Time:	2019-12-04T13:54:32.192
Applied MSP Library(ies):	BDAL, Filamentous Fungi Library 1.0, Mycobacteria Library 1.0 (bead method), IVD, Listeria
Applied Taxonomy Tree:	

Rank (Quality)	Matched Pattern	Score Value	NCBI Identifier
1 (-)	Rothia dentocariosa DSM 43762T DSM	1.68	2047
2 (-)	Weissella viridescens DSM 20248 DSM	1.448	1629
3 (-)	Ralstonia pickettii 21323_1 CHB	1.352	329
4 (-)	Psychrobacillus psychrodurans DSM 11713T DSM	1.34	126157
5 (-)	Rothia dentocariosa CCUG 29965 CCUG	1.321	2047
6 (-)	Hyphomicrobium sp MB64 UFL	1.315	81
7 (-)	Propionibacterium thoenii DSM 20276T DSM	1.314	1751
8 (-)	Propionibacterium thoenii DSM 20277 DSM	1.301	1751
9 (-)	Comamonas kerstersii DSM 16026T HAM	1.297	225992
10 (-)	Paenibacillus phyllosphaerae DSM 17399T DSM	1.287	274593

Analyte122

Analyte Name:	F2
Analyte Description:	
Analyte ID:	C30B
Analyte Creation Date/Time:	2019-12-04T13:54:31.959
Applied MSP Library(ies):	IVD, BDAL, Filamentous Fungi Library 1.0, Mycobacteria Library 1.0 (bead method), Listeria
Applied Taxonomy Tree:	

Rank (Quality)	Matched Pattern	Score Value	NCBI Identifier
1 (-)	Pseudomonas migulae CIP 105470T HAM	1.432	78543
2 (-)	Pseudomonas lutea LMG 21974T HAM	1.385	243924
3 (-)	Lactobacillus vini DSM 20605T DSM	1.363	238015
4 (-)	Chryseobacterium piscicola DSM 21068T DSM	1.326	59732
5 (-)	Clostridium chauvoei 1024_NCTC 8596 BOG	1.303	46867
6 (-)	Rothia dentocariosa CCUG 29965 CCUG	1.282	2047
7 (-)	Pasteurella multocida FI FLR	1.276	747
8 (-)	Pseudomonas koreensis LMG 21318T HAM	1.276	198620
9 (-)	Streptococcus vestibularis DSM 5636T DSM	1.272	1343
10 (-)	Citrobacter freundii 13158_2 CHB	1.259	546

Analyte123

Analyte Name:	F3
Analyte Description:	
Analyte ID:	CN26A
Analyte Creation Date/Time:	2019-12-04T13:54:32.231
Applied MSP Library(ies):	BDAL, Filamentous Fungi Library 1.0, Mycobacteria Library 1.0 (bead method), IVD, Listeria
Applied Taxonomy Tree:	

Rank (Quality)	Matched Pattern	Score Value	NCBI Identifier
1 (-)	Streptococcus infantis DSM 12492T DSM	1.569	68892
2 (-)	Streptococcus salivarius 0807M25049501 IBS	1.511	1304
3 (-)	Streptococcus downei DSM 5635T DSM	1.476	1317
4 (-)	Streptococcus orisratti DSM 15617T DSM	1.459	114652
5 (-)	Streptococcus gordonii DSM 6777T DSM	1.443	1302
6 (-)	Streptococcus gallolyticus ssp pasteurianus DSM 15351T DSM	1.426	197614
7 (-)	Streptococcus equi_ssp_ruminatorum DSM 17037T DSM	1.411	254358
8 (-)	Streptococcus equi_ssp_zooepidemicus ATCC 43079T THL	1.398	40041
9 (-)	Streptococcus dysgalactiae ssp equisimilis DSM 6176 DSM	1.358	119602
10 (-)	Propionibacterium thoenii DSM 20276T DSM	1.352	1751

Analyte124

Analyte Name:	F4
Analyte Description:	
Analyte ID:	CN26A
Analyte Creation Date/Time:	2019-12-04T13:54:32.287
Applied MSP Library(ies):	BDAL, Filamentous Fungi Library 1.0, Mycobacteria Library 1.0 (bead method), IVD, Listeria
Applied Taxonomy Tree:	

Rank (Quality)	Matched Pattern	Score Value	NCBI Identifier
1 (-)	Lactobacillus sharpeae DSM 20506 DSM	1.515	1626
2 (-)	Streptococcus infantis DSM 12492T DSM	1.464	68892
3 (-)	Lactobacillus sharpeae DSM 20504 DSM	1.462	1626
4 (-)	Streptococcus salivarius 0807M25049501 IBS	1.4	1304
5 (-)	Streptococcus sobrinus DSM 20742T DSM	1.392	1310
6 (-)	Streptococcus downei DSM 5635T DSM	1.39	1317
7 (-)	Streptococcus vestibularis CCUG 61229 CCUG	1.37	1343
8 (-)	Streptococcus orisratti DSM 15617T DSM	1.368	114652
9 (-)	Streptococcus gallolyticus ssp pasteurianus DSM 15351T DSM	1.358	197614
10 (-)	Streptococcus oralis DSM 20627T DSM	1.352	1303

Analyte125

Analyte Name:	F5
Analyte Description:	
Analyte ID:	CN26B
Analyte Creation Date/Time:	2019-12-04T13:54:31.856
Applied MSP Library(ies):	BDAL, Filamentous Fungi Library 1.0, Mycobacteria Library 1.0 (bead method), IVD, Listeria
Applied Taxonomy Tree:	

Rank (Quality)	Matched Pattern	Score Value	NCBI Identifier
1 (+)	Rothia dentocariosa CCUG 29965 CCUG	1.839	2047
2 (-)	Rothia mucilaginosa DSM 20445 DSM	1.608	43675
3 (-)	Rothia mucilaginosa CCUG 52532 CCUG	1.576	43675
4 (-)	Rothia mucilaginosa DSM 20446 DSM	1.554	43675
5 (-)	Rothia mucilaginosa BK2995_09 ERL	1.475	43675
6 (-)	Corynebacterium amycolatum 100_28_B77962_42 IBS	1.35	43765
7 (-)	Rothia mucilaginosa DSM 20746T DSM	1.315	43675
8 (-)	Rothia mucilaginosa CCUG 44966 CCUG	1.31	43675
9 (-)	Burkholderia pyrrocinia LMG 14191T HAM	1.286	60550
10 (-)	Weissella viridescens DSM 20248 DSM	1.269	1629

Analyte126

Analyte Name:	F6
Analyte Description:	
Analyte ID:	CN26B
Analyte Creation Date/Time:	2019-12-04T13:54:32.262
Applied MSP Library(ies):	BDAL, Filamentous Fungi Library 1.0, Mycobacteria Library 1.0 (bead method), IVD, Listeria
Applied Taxonomy Tree:	

Rank (Quality)	Matched Pattern	Score Value	NCBI Identifier
1 (-)	Rothia aeria DSM 14556T DSM	1.44	172042
2 (-)	Rothia mucilaginosa DSM 20445 DSM	1.373	43675
3 (-)	Rothia mucilaginosa DSM 20446 DSM	1.346	43675
4 (-)	Sinomonas atrocyanea HKI 10432 HKJ	1.308	37927
5 (-)	Corynebacterium amycolatum PX_25086111 MLD	1.306	43765
6 (-)	Agromyces mediolanus DSM 20152T DSM	1.238	41986
7 (-)	Brevibacillus borstelensis 5_5 TUB	1.231	45462
8 (-)	Sphingomonas faeni DSM 14747T HAM	1.216	185950
9 (-)	Paenibacillus mendelii DSM 19248T DSM	1.208	206163
10 (-)	Weissella viridescens DSM 20248 DSM	1.203	1629

Analyte127

Analyte Name: F7
Analyte Description:
Analyte ID: CN15A
Analyte Creation Date/Time: 2019-12-04T13:54:32.353
Applied MSP Library(ies): BDAL, Filamentous Fungi Library 1.0, Mycobacteria Library 1.0 (bead method), IVD, Listeria
Applied Taxonomy Tree:

Rank (Quality)	Matched Pattern	Score Value	NCBI Identifier
1 (+)	Streptococcus salivarius 0807M25049501 IBS	1.921	1304
2 (+)	Streptococcus vestibularis CCUG 51352 CCUG	1.762	1343
3 (-)	Rothia dentocariosa DSM 43762T DSM	1.611	2047
4 (-)	Streptococcus salivarius DSM 20560T DSM	1.595	1304
5 (-)	Streptococcus vestibularis CCUG 61229 CCUG	1.394	1343
6 (-)	Agromyces mediolanus HKI 308 HKJ	1.379	41986
7 (-)	Agromyces mediolanus HKI 108_DSM 20152T HKJ	1.332	41986
8 (-)	Chryseobacterium scophthalmum LMG 13028T HAM	1.32	59733
9 (-)	Ralstonia sp B484 UFL	1.308	48736
10 (-)	Clostridium cadaveris 1074_ATCC 25783T BOG	1.301	1529

Analyte128

Analyte Name:	F8
Analyte Description:	
Analyte ID:	CN15A
Analyte Creation Date/Time:	2019-12-04T13:54:32.496
Applied MSP Library(ies):	BDAL, Filamentous Fungi Library 1.0, Mycobacteria Library 1.0 (bead method), IVD, Listeria
Applied Taxonomy Tree:	

Rank (Quality)	Matched Pattern	Score Value	NCBI Identifier
1 (+)	Streptococcus salivarius 0807M25049501 IBS	1.829	1304
2 (-)	Streptococcus downei DSM 5635T DSM	1.577	1317
3 (-)	Paenibacillus glucanolyticus DSM 5162T DSM	1.408	59843
4 (-)	Thauera phenylacetica B4P MPB	1.39	164400
5 (-)	Weissella viridescens DSM 20248 DSM	1.382	1629
6 (-)	Brevibacterium casei IMET 10997T HKJ	1.37	33889
7 (-)	Lactobacillus plantarum DSM 2601 DSM	1.361	1590
8 (-)	Rothia dentocariosa DSM 43762T DSM	1.359	2047
9 (-)	Paenibacillus nematophilus DSM 13559T DSM	1.356	200132
10 (-)	Streptococcus sobrinus DSM 20742T DSM	1.348	1310

Analyte129

Analyte Name: F9
Analyte Description:
Analyte ID: CN15B
Analyte Creation Date/Time: 2019-12-04T13:54:32.337
Applied MSP Library(ies): BDAL, Filamentous Fungi Library 1.0, Mycobacteria Library 1.0 (bead method), IVD, Listeria
Applied Taxonomy Tree:

Rank (Quality)	Matched Pattern	Score Value	NCBI Identifier
1 (-)	Pichia occidentalis CBS 1910 CBS	1.539	54552
2 (-)	Clostridium bifermentans 2274_CCUG 35556 A BOG	1.508	1490
3 (-)	Lactobacillus plantarum DSM 20205 DSM	1.482	1590
4 (-)	Filifactor villosus 1051_NCTC 11220T BOG	1.385	29374
5 (-)	Lactobacillus paracasei ssp paracasei DSM 20207 DSM	1.374	47714
6 (-)	Candida parapsilosis ATCC 22019 THL	1.358	5480
7 (-)	Kitasatospora phosalacinea HKI 222 HKJ	1.341	2065
8 (-)	Acinetobacter baumannii B389 UFL	1.313	470
9 (-)	Clostridium difficile MB_7869_05 THL	1.309	1496
10 (-)	Curtobacterium albidum HKI 11500 HKJ	1.302	50728

Analyte130

Analyte Name:	F10
Analyte Description:	
Analyte ID:	CN15B
Analyte Creation Date/Time:	2019-12-04T13:54:31.805
Applied MSP Library(ies):	BDAL, Filamentous Fungi Library 1.0, Mycobacteria Library 1.0 (bead method), IVD, Listeria
Applied Taxonomy Tree:	

Rank (Quality)	Matched Pattern	Score Value	NCBI Identifier
1 (-)	Lactobacillus curvatus DSM 20496 DSM	1.454	28038
2 (-)	Staphylococcus simulans DSM 20324 DSM	1.417	1286
3 (-)	Lactobacillus plantarum DSM 20205 DSM	1.377	1590
4 (-)	Clostridium cadaveris 1074_ATCC 25783T BOG	1.333	1529
5 (-)	Lactobacillus curvatus DSM 20495 DSM	1.326	28038
6 (-)	Stenotrophomonas maltophilia 10942 CHB	1.284	40324
7 (-)	Aeromonas hydrophila CECT 839T DSM	1.277	644
8 (-)	Blastomonas natatoria DSM 3183T HAM	1.273	34015
9 (-)	Candida kefyr CBS 834 CBS	1.271	374272
10 (-)	Weissella halotolerans DSM 20190T DSM	1.268	1615

Analyte131

Analyte Name:	F11
Analyte Description:	
Analyte ID:	CN3A
Analyte Creation Date/Time:	2019-12-04T13:54:32.367
Applied MSP Library(ies):	BDAL, Filamentous Fungi Library 1.0, Mycobacteria Library 1.0 (bead method), IVD, Listeria
Applied Taxonomy Tree:	

Rank (Quality)	Matched Pattern	Score Value	NCBI Identifier
1 (-)	Rothia aeria 120619_15_b HUA	1.682	172042
2 (-)	Streptococcus salivarius 0807M25049501 IBS	1.595	1304
3 (-)	Streptococcus salivarius DSM 20560T DSM	1.53	1304
4 (-)	Rothia aeria CCUG 50760 CCUG	1.488	172042
5 (-)	Streptococcus salivarius ssp salivarius 140417_01 ETL	1.482	1304
6 (-)	Streptococcus salivarius DSM 20560T BRB	1.42	1304
7 (-)	Penicillium discolor_DD MPA 1338 MPA	1.392	123269315
8 (-)	Ochrobactrum anthropi DSM 20150 DSM	1.374	529
9 (-)	Paenibacillus zanthoxyli DSM 18202T DSM	1.345	369399
10 (-)	Rothia dentocariosa CCUG 29965 CCUG	1.325	2047

Analyte132

Analyte Name:	F12
Analyte Description:	
Analyte ID:	CN3A
Analyte Creation Date/Time:	2019-12-04T13:54:32.672
Applied MSP Library(ies):	BDAL, Filamentous Fungi Library 1.0, Mycobacteria Library 1.0 (bead method), IVD, Listeria
Applied Taxonomy Tree:	

Rank (Quality)	Matched Pattern	Score Value	NCBI Identifier
1 (+)	Rothia aeria DSM 14556T DSM	1.819	172042
2 (-)	Rothia aeria CCUG 50760 CCUG	1.637	172042
3 (-)	Streptococcus salivarius ssp salivarius 140417_01 ETL	1.424	1304
4 (-)	Clostridium tetani type 1 1049_NCTC 279T BOG	1.417	1513
5 (-)	Streptococcus salivarius DSM 20560T BRB	1.412	1304
6 (-)	Rothia aeria NO_11 HUA	1.399	172042
7 (-)	Pseudomonas oleovorans DSM 1045T HAM	1.384	301
8 (-)	Streptococcus salivarius DSM 20560T DSM	1.337	1304
9 (-)	Sphingomonas yabuuchiae DSM 14562T HAM	1.291	172044
10 (-)	Clostridium cochlearium 1080_ATCC 17794T BOG	1.289	1494

Analyte133

Analyte Name:	F13
Analyte Description:	
Analyte ID:	CN3B
Analyte Creation Date/Time:	2019-12-04T13:54:32.512
Applied MSP Library(ies):	BDAL, Filamentous Fungi Library 1.0, Mycobacteria Library 1.0 (bead method), IVD, Listeria
Applied Taxonomy Tree:	

Rank (Quality)	Matched Pattern	Score Value	NCBI Identifier
1 (-)	Rothia dentocariosa CCUG 29965 CCUG	1.614	2047
2 (-)	Streptococcus salivarius 0807M25049501 IBS	1.419	1304
3 (-)	Rothia mucilaginosa CCUG 31189 CCUG	1.41	43675
4 (-)	Ralstonia pickettii 21323_1 CHB	1.407	329
5 (-)	Rothia mucilaginosa BK2995_09 ERL	1.337	43675
6 (-)	Arthrobacter roseus DSM 14508T DSM	1.331	136274
7 (-)	Rothia mucilaginosa DSM 20746T DSM	1.284	43675
8 (-)	Lactobacillus sharpeae DSM 20506 DSM	1.267	1626
9 (-)	Rothia mucilaginosa DSM 20445 DSM	1.255	43675
10 (-)	Lactobacillus sharpeae DSM 20504 DSM	1.206	1626

Analyte134

Analyte Name:	F14
Analyte Description:	
Analyte ID:	CN3B
Analyte Creation Date/Time:	2019-12-04T13:54:32.225
Applied MSP Library(ies):	BDAL, Filamentous Fungi Library 1.0, Mycobacteria Library 1.0 (bead method), IVD, Listeria
Applied Taxonomy Tree:	

Rank (Quality)	Matched Pattern	Score Value	NCBI Identifier
1 (+)	Rothia dentocariosa CCUG 29965 CCUG	1.879	2047
2 (+)	Rothia dentocariosa DSM 43762T DSM	1.843	2047
3 (-)	Streptococcus salivarius DSM 20560T DSM	1.54	1304
4 (-)	Streptococcus downei DSM 5635T DSM	1.446	1317
5 (-)	Rothia dentocariosa RV_BA1_032010_D LBK	1.438	2047
6 (-)	Weissella viridescens DSM 20248 DSM	1.438	1629
7 (-)	Rothia mucilaginosa DSM 20746T DSM	1.364	43675
8 (-)	Corynebacterium striatum 23086514 MLD	1.354	43770
9 (-)	Corynebacterium urealyticum CCUG 17231 CCUG	1.328	43771
10 (-)	Streptococcus vestibularis 1C15011954_3 MVD	1.319	1343

Analyte135

Analyte Name:	F15
Analyte Description:	
Analyte ID:	C5A
Analyte Creation Date/Time:	2019-12-04T13:54:31.973
Applied MSP Library(ies):	BDAL, Filamentous Fungi Library 1.0, Mycobacteria Library 1.0 (bead method), IVD, Listeria
Applied Taxonomy Tree:	

Rank (Quality)	Matched Pattern	Score Value	NCBI Identifier
1 (+)	Rothia aeria NO_11 HUA	1.81	172042
2 (-)	Rothia aeria DSM 14556T DSM	1.594	172042
3 (-)	Rothia dentocariosa CCUG 29965 CCUG	1.573	2047
4 (-)	Rothia aeria CCUG 50760 CCUG	1.564	172042
5 (-)	Acidovorax facilis DSM 649T HAM	1.392	12917
6 (-)	Paenibacillus xylanilyticus DSM 17255T DSM	1.346	248903
7 (-)	Paenibacillus brasilensis DSM 14914T DSM	1.32	128574
8 (-)	Rothia dentocariosa G18709 IBS	1.293	2047
9 (-)	Photorhabdus temperata ssp temperata DSM 14550T HAM	1.279	171441
10 (-)	Escherichia coli DH5alpha BRL	1.257	562

Analyte136

Analyte Name:	F16
Analyte Description:	
Analyte ID:	C5A
Analyte Creation Date/Time:	2019-12-04T13:54:31.699
Applied MSP Library(ies):	BDAL, Filamentous Fungi Library 1.0, Mycobacteria Library 1.0 (bead method), IVD, Listeria
Applied Taxonomy Tree:	

Rank (Quality)	Matched Pattern	Score Value	NCBI Identifier
1 (-)	Agromyces salentinus HKI 320_DSM 16198T HKJ	1.363	269421
2 (-)	Paenibacillus jamilae DSM 13815T DSM	1.362	114136
3 (-)	Rothia aeria DSM 14556T DSM	1.318	172042
4 (-)	Paenibacillus zanthoxyli DSM 18202T DSM	1.315	369399
5 (-)	Paenibacillus polymyxa DSM 740 DSM	1.308	1406
6 (-)	Rothia dentocariosa DSM 43762T DSM	1.306	2047
7 (-)	Paenibacillus stellifer DSM 14472T DSM	1.293	169760
8 (-)	Paenibacillus massiliensis DSM 16942T DSM	1.284	225917
9 (-)	Paenibacillus brasilensis DSM 14914T DSM	1.269	128574
10 (-)	Lactobacillus equi DSM 15833T DSM	1.254	137357

Analyte137

Analyte Name:	F17
Analyte Description:	
Analyte ID:	C5B
Analyte Creation Date/Time:	2019-12-04T13:54:32.616
Applied MSP Library(ies):	BDAL, Filamentous Fungi Library 1.0, Mycobacteria Library 1.0 (bead method), IVD, Listeria
Applied Taxonomy Tree:	

Rank (Quality)	Matched Pattern	Score Value	NCBI Identifier
1 (++)	Rothia dentocariosa CCUG 29965 CCUG	2.128	2047
2 (+)	Rothia dentocariosa DSM 43762T DSM	1.866	2047
3 (-)	Rothia dentocariosa G6496_ch28 IBS	1.556	2047
4 (-)	Rothia mucilaginosa DSM 20445 DSM	1.522	43675
5 (-)	Rothia dentocariosa RV_BA1_032010_D LBK	1.44	2047
6 (-)	Rothia mucilaginosa DSM 20746T DSM	1.432	43675
7 (-)	Rothia dentocariosa B16575_bh8 IBS	1.415	2047
8 (-)	Burkholderia pyrrocinia LMG 14191T HAM	1.331	60550
9 (-)	Rothia mucilaginosa CCUG 44966 CCUG	1.329	43675
10 (-)	Ralstonia pickettii 21323_1 CHB	1.324	329

Analyte138

Analyte Name:	F18
Analyte Description:	
Analyte ID:	C5B
Analyte Creation Date/Time:	2019-12-04T13:54:32.042
Applied MSP Library(ies):	BDAL, Filamentous Fungi Library 1.0, Mycobacteria Library 1.0 (bead method), IVD, Listeria
Applied Taxonomy Tree:	

Rank (Quality)	Matched Pattern	Score Value	NCBI Identifier
1 (+)	Rothia dentocariosa DSM 43762T DSM	1.896	2047
2 (-)	Rothia dentocariosa CCUG 29965 CCUG	1.668	2047
3 (-)	Candida kefyr CBS 834 CBS	1.487	374272
4 (-)	Agromyces bracchium HKI 303 DSM 14596T HKJ	1.452	88376
5 (-)	Cupriavidus necator DSM 531 HAM	1.376	106590
6 (-)	Rothia dentocariosa G6496_ch28 IBS	1.324	2047
7 (-)	Paenibacillus lactis DSM 15596T DSM	1.321	228574
8 (-)	Ochrobactrum intermedium LMG 3301T HAM	1.311	94625
9 (-)	Corynebacterium confusum DSM 44384T DSM	1.288	71254
10 (-)	Lactobacillus gasseri DSM 20604 DSM	1.286	1596

Analyte139

Analyte Name:	F19
Analyte Description:	
Analyte ID:	C5C
Analyte Creation Date/Time:	2019-12-04T13:54:31.990
Applied MSP Library(ies):	BDAL, Filamentous Fungi Library 1.0, Mycobacteria Library 1.0 (bead method), IVD, Listeria
Applied Taxonomy Tree:	

Rank (Quality)	Matched Pattern	Score Value	NCBI Identifier
1 (+)	Rothia dentocariosa DSM 43762T DSM	1.917	2047
2 (-)	Rothia mucilaginosa DSM 20746T DSM	1.511	43675
3 (-)	Rothia dentocariosa CCUG 29965 CCUG	1.491	2047
4 (-)	Arthrobacter stackebrandtii DSM 16005T DSM	1.346	272161
5 (-)	Rothia mucilaginosa BK2995_09 ERL	1.294	43675
6 (-)	Weissella viridescens DSM 20248 DSM	1.265	1629
7 (-)	Paenibacillus lactis DSM 15596T DSM	1.251	228574
8 (-)	Trichosporon mucoides ATCC 204094 THL	1.247	82522
9 (-)	Vibrio gazogenes LMG 19540T HAM	1.241	687
10 (-)	Paenibacillus taiwanensis DSM 18679T DSM	1.209	401638

Analyte140

Analyte Name:	F20
Analyte Description:	
Analyte ID:	C5C
Analyte Creation Date/Time:	2019-12-04T13:54:32.571
Applied MSP Library(ies):	BDAL, Filamentous Fungi Library 1.0, Mycobacteria Library 1.0 (bead method), IVD, Listeria
Applied Taxonomy Tree:	

Rank (Quality)	Matched Pattern	Score Value	NCBI Identifier
1 (+)	Rothia dentocariosa DSM 43762T DSM	1.841	2047
2 (-)	Pseudomonas congelans DSM 14939T HAM	1.464	200452
3 (-)	Rothia dentocariosa RV_BA1_032010_D LBK	1.46	2047
4 (-)	Rothia dentocariosa CCUG 29965 CCUG	1.365	2047
5 (-)	Rothia mucilaginosa CCUG 52532 CCUG	1.322	43675
6 (-)	Arthrobacter stackebrandtii DSM 16005T DSM	1.316	272161
7 (-)	Arthrobacter histidinolovorans DSM 20115T DSM	1.286	43664
8 (-)	Novosphingobium resinovorum DSM 7478T HAM	1.263	158500
9 (-)	Lactobacillus paracasei ssp tolerans DSM 20012 DSM	1.24	113557
10 (-)	Staphylococcus warneri DSM 20036 DSM	1.239	1292

Analyte141

Analyte Name:	F21
Analyte Description:	
Analyte ID:	CN13
Analyte Creation Date/Time:	2019-12-04T13:54:31.667
Applied MSP Library(ies):	BDAL, Filamentous Fungi Library 1.0, Mycobacteria Library 1.0 (bead method), IVD, Listeria
Applied Taxonomy Tree:	

Rank (Quality)	Matched Pattern	Score Value	NCBI Identifier
1 (+)	Rothia mucilaginosa BK2995_09 ERL	1.993	43675
2 (+)	Rothia mucilaginosa DSM 20445 DSM	1.736	43675
3 (+)	Rothia mucilaginosa CCUG 44966 CCUG	1.714	43675
4 (-)	Rothia mucilaginosa CCUG 31189 CCUG	1.521	43675
5 (-)	Rothia mucilaginosa DSM 30548 DSM	1.491	43675
6 (-)	Rothia mucilaginosa DSM 20446 BRB	1.411	43675
7 (-)	Streptococcus salivarius DSM 20560T DSM	1.402	1304
8 (-)	Burkholderia pyrrocinia LMG 14191T HAM	1.402	60550
9 (-)	Pseudomonas lutea LMG 21974T HAM	1.375	243924
10 (-)	Corynebacterium confusum DSM 44384T DSM	1.326	71254

Analyte142

Analyte Name:	F22
Analyte Description:	
Analyte ID:	CN13
Analyte Creation Date/Time:	2019-12-04T13:54:32.113
Applied MSP Library(ies):	BDAL, Filamentous Fungi Library 1.0, Mycobacteria Library 1.0 (bead method), IVD, Listeria
Applied Taxonomy Tree:	

Rank (Quality)	Matched Pattern	Score Value	NCBI Identifier
1 (++)	Rothia mucilaginosa BK2995_09 ERL	2.156	43675
2 (++)	Rothia mucilaginosa CCUG 44966 CCUG	2.016	43675
3 (+)	Rothia mucilaginosa DSM 20445 DSM	1.94	43675
4 (+)	Rothia mucilaginosa CCUG 31189 CCUG	1.918	43675
5 (+)	Rothia mucilaginosa CCUG 52532 CCUG	1.77	43675
6 (-)	Rothia dentocariosa DSM 43762T DSM	1.674	2047
7 (-)	Rothia mucilaginosa DSM 30548 DSM	1.66	43675
8 (-)	Streptococcus salivarius DSM 20560T DSM	1.605	1304
9 (-)	Rothia dentocariosa CCUG 29965 CCUG	1.576	2047
10 (-)	Rothia mucilaginosa DSM 20746T DSM	1.526	43675

Analyte143

Analyte Name:	F23
Analyte Description:	
Analyte ID:	CN4A
Analyte Creation Date/Time:	2019-12-04T13:54:31.864
Applied MSP Library(ies):	Listeria, BDAL, Filamentous Fungi Library 1.0, Mycobacteria Library 1.0 (bead method), IVD
Applied Taxonomy Tree:	

Rank (Quality)	Matched Pattern	Score Value	NCBI Identifier
1 (-)	Rothia dentocariosa RV_BA1_032010_D LBK	1.668	2047
2 (-)	Rothia dentocariosa DSM 43762T DSM	1.658	2047
3 (-)	Rothia mucilaginosa DSM 20746T DSM	1.608	43675
4 (-)	Rothia dentocariosa B16575_bh8 IBS	1.563	2047
5 (-)	Rothia mucilaginosa BK2995_09 ERL	1.528	43675
6 (-)	Rothia dentocariosa CCUG 29965 CCUG	1.526	2047
7 (-)	Rothia mucilaginosa CCUG 44966 CCUG	1.484	43675
8 (-)	Streptococcus salivarius DSM 20560T DSM	1.478	1304
9 (-)	Rothia mucilaginosa DSM 20446 DSM	1.459	43675
10 (-)	Streptococcus salivarius ssp salivarius 140417_01 ETL	1.446	1304

Analyte144

Analyte Name:	F24
Analyte Description:	
Analyte ID:	CN4A
Analyte Creation Date/Time:	2019-12-04T13:54:31.777
Applied MSP Library(ies):	BDAL, Filamentous Fungi Library 1.0, Mycobacteria Library 1.0 (bead method), IVD, Listeria
Applied Taxonomy Tree:	

Rank (Quality)	Matched Pattern	Score Value	NCBI Identifier
1 (+)	Rothia dentocariosa DSM 43762T DSM	1.763	2047
2 (+)	Rothia dentocariosa RV_BA1_032010_D LBK	1.723	2047
3 (+)	Rothia dentocariosa CCUG 29965 CCUG	1.708	2047
4 (-)	Rothia mucilaginosa DSM 20746T DSM	1.637	43675
5 (-)	Rothia mucilaginosa CCUG 44966 CCUG	1.486	43675
6 (-)	Streptococcus salivarius 0807M25049501 IBS	1.472	1304
7 (-)	Rothia dentocariosa G6496_ch28 IBS	1.468	2047
8 (-)	Corynebacterium pilosum DSM 20521T DSM	1.374	35756
9 (-)	Ralstonia pickettii 21323_1 CHB	1.355	329
10 (-)	Weissella viridescens DSM 20248 DSM	1.342	1629

Analyte145

Analyte Name:	G1
Analyte Description:	
Analyte ID:	CN4B
Analyte Creation Date/Time:	2019-12-04T13:54:31.672
Applied MSP Library(ies):	BDAL, Filamentous Fungi Library 1.0, Mycobacteria Library 1.0 (bead method), IVD, Listeria
Applied Taxonomy Tree:	

Rank (Quality)	Matched Pattern	Score Value	NCBI Identifier
1 (+)	Rothia dentocariosa DSM 43762T DSM	1.762	2047
2 (+)	Rothia dentocariosa CCUG 29965 CCUG	1.738	2047
3 (-)	Rothia mucilaginosa DSM 20445 DSM	1.352	43675
4 (-)	Arthrobacter stackebrandtii DSM 16005T DSM	1.288	272161
5 (-)	Curtobacterium luteum HKI 10360 HKJ	1.28	33881
6 (-)	Arthrobacter sulfonivorans DSM 14002T DSM	1.28	121292
7 (-)	Corynebacterium amycolatum PX_25086111 MLD	1.24	43765
8 (-)	Agromyces mediolanus HKI 308 HKJ	1.218	41986
9 (-)	Arthrobacter pascens DSM 20545T DSM	1.209	1677
10 (-)	Agromyces italicus HKI 325_DSM 16388T HKJ	1.203	279572

Analyte146

Analyte Name:	G2
Analyte Description:	
Analyte ID:	CN4B
Analyte Creation Date/Time:	2019-12-04T13:54:32.252
Applied MSP Library(ies):	BDAL, Filamentous Fungi Library 1.0, Mycobacteria Library 1.0 (bead method), IVD, Listeria
Applied Taxonomy Tree:	

Rank (Quality)	Matched Pattern	Score Value	NCBI Identifier
1 (-)	Candida guilliermondii CBS 566 CBS	1.461	4929
2 (-)	Pichia occidentalis CBS 1910 CBS	1.455	54552
3 (-)	Austwickia chelonae DSM 44178T DSM	1.453	100225
4 (-)	Lactobacillus paracasei ssp paracasei DSM 5622T DSM	1.446	47714
5 (-)	Lactobacillus paracasei ssp paracasei DSM 8741 DSM	1.446	47714
6 (-)	Rhizobium radiobacter B166 UFL	1.41	358
7 (-)	Lactobacillus paracasei ssp tolerans DSM 20012 DSM	1.381	113557
8 (-)	Clostridium chauvoei 1024_NCTC 8596 BOG	1.369	46867
9 (-)	Lactobacillus satsumensis DSM 16230T DSM	1.362	259059
10 (-)	Rothia dentocariosa DSM 43762T DSM	1.357	2047

Analyte147

Analyte Name:	G3
Analyte Description:	
Analyte ID:	CN5A
Analyte Creation Date/Time:	2019-12-04T13:54:32.417
Applied MSP Library(ies):	BDAL, Filamentous Fungi Library 1.0, Mycobacteria Library 1.0 (bead method), IVD, Listeria
Applied Taxonomy Tree:	

Rank (Quality)	Matched Pattern	Score Value	NCBI Identifier
1 (-)	Staphylococcus simulans DSM 20324 DSM	1.477	1286
2 (-)	Mycobacterium tuberculosis W148 R_722_HI PGM	1.474	1773
3 (-)	Arthrobacter ramosus IMET 10685T HKJ	1.474	1672
4 (-)	Lactobacillus paracasei ssp paracasei DSM 20207 DSM	1.414	47714
5 (-)	Staphylococcus warneri CCM 2604 CCM	1.413	1292
6 (-)	Clostridium baratii 1084_ATCC 25782 BOG	1.407	1561
7 (-)	Actinomyces meyeri DSM 20733T DSM	1.364	52773
8 (-)	Streptomyces badius B192 UFL	1.34	1941
9 (-)	Clostridium bifermentans 2274_CCUG 35556 A BOG	1.336	1490
10 (-)	Candida lusitaniae CBS 4413T CBS	1.32	36911

Analyte148

Analyte Name:	G4
Analyte Description:	
Analyte ID:	CN5A
Analyte Creation Date/Time:	2019-12-04T13:54:31.725
Applied MSP Library(ies):	BDAL, Filamentous Fungi Library 1.0, Mycobacteria Library 1.0 (bead method), IVD, Listeria
Applied Taxonomy Tree:	

Rank (Quality)	Matched Pattern	Score Value	NCBI Identifier
1 (-)	Pichia occidentalis CBS 1910 CBS	1.578	54552
2 (-)	Arthrobacter ramosus IMET 10685T HKJ	1.511	1672
3 (-)	Candida lambica CBS 603 CBS	1.471	53655
4 (-)	Pantoea agglomerans CCM 298 CCM	1.386	549
5 (-)	Pseudomonas oryzihabitans DSM 6835T HAM	1.377	47885
6 (-)	Aeromonas eucrenophila CECT 4224T DSM	1.346	649
7 (-)	Lactobacillus oligofermentans DSM 15709 DSM	1.339	293371
8 (-)	Colletotrichum gloeosporioides CBS 100471 CBS	1.338	474922
9 (-)	Lactobacillus graminis DSM 20719T DSM	1.33	60519
10 (-)	Aspergillus niger 1069 PFM	1.321	5061

Analyte149

Analyte Name:	G5
Analyte Description:	
Analyte ID:	CN5B
Analyte Creation Date/Time:	2019-12-04T13:54:32.183
Applied MSP Library(ies):	BDAL, Filamentous Fungi Library 1.0, Mycobacteria Library 1.0 (bead method), IVD, Listeria
Applied Taxonomy Tree:	

Rank (Quality)	Matched Pattern	Score Value	NCBI Identifier
1 (-)	Rothia dentocariosa B16575_bh8 IBS	1.599	2047
2 (-)	Rothia dentocariosa DSM 43762T DSM	1.594	2047
3 (-)	Rothia dentocariosa CCUG 29965 CCUG	1.501	2047
4 (-)	Streptococcus pneumoniae DSM 20566T DSM	1.421	1313
5 (-)	Arthrobacter oxydans DSM 20119T DSM	1.405	1671
6 (-)	Staphylococcus auricularis DSM 20609 DSM	1.362	29379
7 (-)	Rothia dentocariosa G6496_ch28 IBS	1.346	2047
8 (-)	Paenibacillus brasilensis DSM 14914T DSM	1.338	128574
9 (-)	Streptococcus pneumoniae NRZ 28221 NRZ	1.333	1313
10 (-)	Streptococcus pneumoniae V17_201197 MUZ	1.333	1313

Analyte150

Analyte Name:	G6
Analyte Description:	
Analyte ID:	CN5B
Analyte Creation Date/Time:	2019-12-04T13:54:32.119
Applied MSP Library(ies):	Listeria, IVD, Mycobacteria Library 1.0 (bead method), Filamentous Fungi Library 1.0, BDAL
Applied Taxonomy Tree:	

Rank (Quality)	Matched Pattern	Score Value	NCBI Identifier
1 (+)	Rothia dentocariosa DSM 43762T DSM	1.741	2047
2 (-)	Paenibacillus alvei DSM 5557 DSM	1.492	44250
3 (-)	Citrobacter freundii 13158_2 CHB	1.403	546
4 (-)	Pseudomonas straminea CIP 106745T HAM	1.373	47882
5 (-)	Hydrogenophaga flava B339 UFL	1.368	65657
6 (-)	Paenibacillus illinoisensis DSM 11733T DSM	1.358	59845
7 (-)	Cellulosimicrobium cellulans B480 UFL	1.342	1710
8 (-)	Sodalis glossinidius DSM 16929T HAM	1.316	63612
9 (-)	Corynebacterium confusum DSM 44384T DSM	1.307	71254
10 (-)	Curtobacterium luteum HKI 10360 HKJ	1.307	33881

Analyte151

Analyte Name:	G7
Analyte Description:	
Analyte ID:	CN18A
Analyte Creation Date/Time:	2019-12-04T13:54:32.100
Applied MSP Library(ies):	BDAL, Filamentous Fungi Library 1.0, Mycobacteria Library 1.0 (bead method), IVD, Listeria
Applied Taxonomy Tree:	

Rank (Quality)	Matched Pattern	Score Value	NCBI Identifier
1 (-)	Aeromonas molluscorum 848T DSM	1.417	271417
2 (-)	Rothia aeria CCUG 50760 CCUG	1.404	172042
3 (-)	Lactobacillus kefiri DSM 20587T DSM	1.36	33962
4 (-)	Rothia aeria DSM 14556T DSM	1.335	172042
5 (-)	Streptococcus salivarius DSM 20560T DSM	1.311	1304
6 (-)	Arthrobacter pyridinolis B384 UFL	1.266	1663
7 (-)	Lactobacillus curvatus DSM 20010 DSM	1.24	28038
8 (-)	Lactobacillus antri DSM 16041T DSM	1.236	227943
9 (-)	Shewanella baltica DSM 9439T HAM	1.227	62322
10 (-)	Lactobacillus curvatus DSM 20499 DSM	1.227	28038

Analyte152

Analyte Name: G8
Analyte Description:
Analyte ID: CN18A
Analyte Creation Date/Time: 2019-12-04T13:54:31.945
Applied MSP Library(ies): BDAL, Filamentous Fungi Library 1.0, Mycobacteria Library 1.0 (bead method), IVD, Listeria
Applied Taxonomy Tree:

Rank (Quality)	Matched Pattern	Score Value	NCBI Identifier
1 (+)	Rothia aeria CCUG 25688 CCUG	1.815	172042
2 (+)	Rothia aeria CCUG 50760 CCUG	1.749	172042
3 (+)	Rothia aeria 120619_15_b HUA	1.704	172042
4 (-)	Rothia aeria DSM 14556T DSM	1.451	172042
5 (-)	Rothia aeria NO_11 HUA	1.431	172042
6 (-)	Streptococcus salivarius 0807M25049501 IBS	1.398	1304
7 (-)	Flavobacterium hydatis DSM 2063T HAM	1.325	991
8 (-)	Blastomonas natatoria DSM 3183T HAM	1.307	34015
9 (-)	Paenibacillus polymyxa DSM 356 DSM	1.294	1406
10 (-)	Streptomyces hirsutus B267 UFL	1.286	35620

Analyte153

Analyte Name:	G9
Analyte Description:	
Analyte ID:	CN18B
Analyte Creation Date/Time:	2019-12-04T13:54:32.168
Applied MSP Library(ies):	BDAL, Filamentous Fungi Library 1.0, Mycobacteria Library 1.0 (bead method), IVD, Listeria
Applied Taxonomy Tree:	

Rank (Quality)	Matched Pattern	Score Value	NCBI Identifier
1 (-)	Rothia mucilaginosa BK2995_09 ERL	1.61	43675
2 (-)	Rothia mucilaginosa CCUG 31189 CCUG	1.583	43675
3 (-)	Rothia dentocariosa B16575_bh8 IBS	1.474	2047
4 (-)	Janthinobacterium lividum CIP 106720T HAM	1.469	29581
5 (-)	Rothia mucilaginosa DSM 20446 BRB	1.446	43675
6 (-)	Paenibacillus amylolyticus DSM 15211T DSM	1.436	1451
7 (-)	Rothia dentocariosa DSM 43762T DSM	1.422	2047
8 (-)	Comamonas terrigena DSM 7099T HAM	1.42	32013
9 (-)	Rothia mucilaginosa DSM 20445 DSM	1.366	43675
10 (-)	Paenibacillus phyllosphaerae DSM 17399T DSM	1.359	274593

Analyte154

Analyte Name: G10
Analyte Description:
Analyte ID: CN18B
Analyte Creation Date/Time: 2019-12-04T13:54:32.658
Applied MSP Library(ies): BDAL, Filamentous Fungi Library 1.0, Mycobacteria Library 1.0 (bead method), IVD, Listeria
Applied Taxonomy Tree:

Rank (Quality)	Matched Pattern	Score Value	NCBI Identifier
1 (+)	Rothia dentocariosa CCUG 29965 CCUG	1.733	2047
2 (-)	Rothia mucilaginosa CCUG 31189 CCUG	1.604	43675
3 (-)	Rothia dentocariosa DSM 43762T DSM	1.593	2047
4 (-)	Rothia mucilaginosa BK2995_09 ERL	1.571	43675
5 (-)	Burkholderia pyrrocinia LMG 14191T HAM	1.546	60550
6 (-)	Burkholderia anthina LMG 16670 HAM	1.541	179879
7 (-)	Burkholderia stabilis LMG 14294T HAM	1.533	95485
8 (-)	Burkholderia cepacia LMG 2161 HAM	1.512	292
9 (-)	Rothia dentocariosa RV_BA1_032010_D LBK	1.471	2047
10 (-)	Burkholderia cenocepacia LMG 12614 HAM	1.462	95486

Analyte155

Analyte Name:	G11
Analyte Description:	
Analyte ID:	CN32A
Analyte Creation Date/Time:	2019-12-04T13:54:32.148
Applied MSP Library(ies):	Listeria, IVD, Mycobacteria Library 1.0 (bead method), Filamentous Fungi Library 1.0, BDAL
Applied Taxonomy Tree:	

Rank (Quality)	Matched Pattern	Score Value	NCBI Identifier
1 (-)	Neisseria sicca DSM 17713T DSM	1.444	490
2 (-)	Neisseria meningitidis Serogroup_Y BRL	1.356	487
3 (-)	Aeromonas schubertii CECT 4240T DSM	1.355	652
4 (-)	Weissella minor DSM 20014T DSM	1.321	1620
5 (-)	Pseudomonas kilonensis DSM 13647T HAM	1.266	132476
6 (-)	Pseudomonas umsongensis LMG 21317T HAM	1.259	198618
7 (-)	Staphylococcus xylosus DSM 20267 DSM	1.238	1288
8 (-)	Neisseria meningitidis 24086406 MLD	1.232	487
9 (-)	Lactococcus garvieae DSM 20684T DSM	1.218	1363
10 (-)	Corynebacterium thomssenii DSM 44276T DSM	1.215	62629

Analyte156

Analyte Name:	G12
Analyte Description:	
Analyte ID:	CN32A
Analyte Creation Date/Time:	2019-12-04T13:54:32.105
Applied MSP Library(ies):	BDAL, Filamentous Fungi Library 1.0, Mycobacteria Library 1.0 (bead method), IVD, Listeria
Applied Taxonomy Tree:	

Rank (Quality)	Matched Pattern	Score Value	NCBI Identifier
1 (-)	Neisseria mucosa DSM 17611T DSM	1.576	488
2 (-)	Neisseria gonorrhoeae ATCC 49226 THL	1.552	485
3 (-)	Neisseria subflava DSM 17610T DSM_2	1.381	28449
4 (-)	Pseudomonas fragi DSM 3456T HAM	1.31	296
5 (-)	Neisseria meningitidis Serogroup_X BRL	1.294	487
6 (-)	Neisseria subflava 1672 PGM	1.294	28449
7 (-)	Pseudomonas agarici DSM 11810T HAM	1.292	46677
8 (-)	Enterobacter cloacae DSM 3264 DSM	1.272	550
9 (-)	Lactococcus garvieae DSM 20684T DSM	1.252	1363
10 (-)	Ralstonia pickettii 21323_1 CHB	1.238	329

Analyte157

Analyte Name:	G13
Analyte Description:	
Analyte ID:	CN32B
Analyte Creation Date/Time:	2019-12-04T13:54:32.341
Applied MSP Library(ies):	BDAL, Filamentous Fungi Library 1.0, Mycobacteria Library 1.0 (bead method), IVD, Listeria
Applied Taxonomy Tree:	

Rank (Quality)	Matched Pattern	Score Value	NCBI Identifier
1 (-)	Paenibacillus xinjiangensis DSM 16970T DSM	1.452	459527
2 (-)	Rothia mucilaginosa CCUG 52532 CCUG	1.284	43675
3 (-)	Aeromonas veronii CECT 4199T DSM	1.279	654
4 (-)	Castellaniella defragrans DSM 12141T HAM	1.253	75697
5 (-)	Pseudomonas fragi DSM 3456T HAM	1.22	296
6 (-)	Salmonella sp (enterica st Dublin) Sa05_188 VAB	1.199	98360
7 (-)	Rothia dentocariosa G6496_ch28 IBS	1.189	2047
8 (-)	Arthrobacter russicus DSM 14555T DSM	1.171	172040
9 (-)	Neisseria meningitidis Serogroup_A BRL	1.168	487
10 (-)	Candida dubliniensis CBS 8500 CBS	1.162	42374

Analyte158

Analyte Name:	G14
Analyte Description:	
Analyte ID:	CN32B
Analyte Creation Date/Time:	2019-12-04T13:54:31.836
Applied MSP Library(ies):	BDAL, Filamentous Fungi Library 1.0, Mycobacteria Library 1.0 (bead method), IVD, Listeria
Applied Taxonomy Tree:	

Rank (Quality)	Matched Pattern	Score Value	NCBI Identifier
1 (-)	Rothia mucilaginosa CCUG 52532 CCUG	1.421	43675
2 (-)	Rothia mucilaginosa BK2995_09 ERL	1.399	43675
3 (-)	Clostridium novyi A 1025_NCTC 538 BOG	1.261	1542
4 (-)	Sphingomonas adhaesiva DSM 7418T HAM	1.208	28212
5 (-)	Castellaniella defragrans DSM 12141T HAM	1.206	75697
6 (-)	Pantoea agglomerans CCM 4412 CCM	1.203	549
7 (-)	Candida dubliniensis SA 121 CBS	1.202	42374
8 (-)	Morganella morganii ssp morganii 15284_1 CHB	1.181	180434
9 (-)	Microbacterium terregens DSM 20449T DSM	1.171	69363
10 (-)	Streptomyces violaceoruber B263 UFL	1.164	1935

Analyte159

Analyte Name:	G15
Analyte Description:	
Analyte ID:	CN32C
Analyte Creation Date/Time:	2019-12-04T13:54:32.522
Applied MSP Library(ies):	BDAL, Filamentous Fungi Library 1.0, Mycobacteria Library 1.0 (bead method), IVD, Listeria
Applied Taxonomy Tree:	

Rank (Quality)	Matched Pattern	Score Value	NCBI Identifier
1 (-)	Pseudomonas aeruginosa DSM 50071T HAM	1.41	287
2 (-)	Pseudomonas aeruginosa 8147_2 CHB	1.398	287
3 (-)	Clostridium novyi A 1025_NCTC 538 BOG	1.289	1542
4 (-)	Colletotrichum gloeosporioides CBS 100471 CBS	1.253	474922
5 (-)	Escherichia coli ATCC 25922 CHB	1.248	562
6 (-)	Rhizobium radiobacter B166 UFL	1.227	358
7 (-)	Corynebacterium macginleyi DSM 44284T DSM	1.223	38290
8 (-)	Candida glabrata ATCC 2001T THL	1.215	5478
9 (-)	Lactobacillus equi DSM 15833T DSM	1.21	137357
10 (-)	Staphylococcus felis DSM 7377T DSM	1.206	46127

Analyte160

Analyte Name:	G16
Analyte Description:	
Analyte ID:	CN32C
Analyte Creation Date/Time:	2019-12-04T13:54:32.579
Applied MSP Library(ies):	BDAL, Filamentous Fungi Library 1.0, Mycobacteria Library 1.0 (bead method), IVD, Listeria
Applied Taxonomy Tree:	

Rank (Quality)	Matched Pattern	Score Value	NCBI Identifier
1 (-)	Agromyces rhizospherae HKI 302_DSM 14597T HKJ	1.494	88374
2 (-)	Lactobacillus equi DSM 15833T DSM	1.343	137357
3 (-)	Thauera mechernichensis Tl1 MPB	1.326	82788
4 (-)	Lactobacillus gastricus DSM 16045T DSM	1.295	227942
5 (-)	Lactobacillus curvatus DSM 20019T DSM	1.282	28038
6 (-)	Lactobacillus paracasei ssp paracasei DSM 5622T DSM	1.261	47714
7 (-)	Lactobacillus pantheris DSM 15945T DSM	1.26	171523
8 (-)	Lactobacillus zeae DSM 20178T DSM	1.253	57037
9 (-)	Bacillus litoralis DSM 16303T DSM	1.224	152268
10 (-)	Lactobacillus sharpeae DSM 20507 DSM	1.219	1626

Analyte161

Analyte Name:	G17
Analyte Description:	
Analyte ID:	CN20A
Analyte Creation Date/Time:	2019-12-04T13:54:32.390
Applied MSP Library(ies):	BDAL, Filamentous Fungi Library 1.0, Mycobacteria Library 1.0 (bead method), IVD, Listeria
Applied Taxonomy Tree:	

Rank (Quality)	Matched Pattern	Score Value	NCBI Identifier
1 (-)	Trichosporon mucoides ATCC 204094 THL	1.355	82522
2 (-)	Rothia dentocariosa DSM 43762T DSM	1.327	2047
3 (-)	Corynebacterium afermentans ssp afermentans DSM 44280T DSM	1.303	144183
4 (-)	Lactobacillus saerimneri DSM 16027 DSM	1.286	228229
5 (-)	Clostridium novyi A 1025_NCTC 538 BOG	1.264	1542
6 (-)	Lactobacillus paracasei ssp paracasei DSM 20207 DSM	1.239	47714
7 (-)	Streptococcus salivarius DSM 20560T DSM	1.237	1304
8 (-)	Arthrobacter pascens DSM 20545T DSM	1.236	1677
9 (-)	Rothia mucilaginosa DSM 20746T DSM	1.225	43675
10 (-)	Staphylococcus xylosus DSM 20266T DSM	1.221	1288

Analyte162

Analyte Name:	G18
Analyte Description:	
Analyte ID:	CN20A
Analyte Creation Date/Time:	2019-12-04T13:54:32.363
Applied MSP Library(ies):	BDAL, Filamentous Fungi Library 1.0, Mycobacteria Library 1.0 (bead method), IVD, Listeria
Applied Taxonomy Tree:	

Rank (Quality)	Matched Pattern	Score Value	NCBI Identifier
1 (-)	Rothia dentocariosa DSM 43762T DSM	1.588	2047
2 (-)	Lactobacillus paracasei ssp paracasei DSM 20207 DSM	1.502	47714
3 (-)	Paenibacillus xinjiangensis DSM 16970T DSM	1.387	459527
4 (-)	Clostridium novyi A 1025_NCTC 538 BOG	1.347	1542
5 (-)	Arthrobacter sulfureus B571 UFL	1.294	43666
6 (-)	Staphylococcus aureus ssp aureus DSM 4910 DSM	1.282	46170
7 (-)	Lactobacillus antri DSM 16041T DSM	1.276	227943
8 (-)	Agromyces italicus HKI 325_DSM 16388T HKJ	1.251	279572
9 (-)	Lactobacillus plantarum DSM 20205 DSM	1.225	1590
10 (-)	Rothia dentocariosa CCUG 29965 CCUG	1.208	2047

Analyte163

Analyte Name:	G19
Analyte Description:	
Analyte ID:	CN20B
Analyte Creation Date/Time:	2019-12-04T13:54:31.786
Applied MSP Library(ies):	BDAL, Filamentous Fungi Library 1.0, Mycobacteria Library 1.0 (bead method), IVD, Listeria
Applied Taxonomy Tree:	

Rank (Quality)	Matched Pattern	Score Value	NCBI Identifier
1 (-)	Staphylococcus epidermidis ATCC 12228 THL	1.553	1282
2 (-)	Lactobacillus paracasei ssp paracasei DSM 20207 DSM	1.331	47714
3 (-)	Lactobacillus salivarius DSM 20492 DSM	1.3	1624
4 (-)	Clostridium sphenoides 1046_NCTC 507T BOG	1.288	29370
5 (-)	Staphylococcus epidermidis ATCC 14990T THL	1.272	1282
6 (-)	Clostridium spiroforme 1047_NCTC 11211T BOG	1.254	29348
7 (-)	Staphylococcus xylosus DSM 20267 DSM	1.23	1288
8 (-)	Pseudomonas caricapapayae LMG 2152T HAM	1.206	46678
9 (-)	Staphylococcus epidermidis ATCC 12228 CHB	1.19	1282
10 (-)	Lactobacillus mali DSM 20483 DSM	1.184	1618

Analyte164

Analyte Name:	G20
Analyte Description:	
Analyte ID:	CN20B
Analyte Creation Date/Time:	2019-12-04T13:54:31.676
Applied MSP Library(ies):	BDAL, Filamentous Fungi Library 1.0, Mycobacteria Library 1.0 (bead method), IVD, Listeria
Applied Taxonomy Tree:	

Rank (Quality)	Matched Pattern	Score Value	NCBI Identifier
1 (-)	Staphylococcus epidermidis ATCC 12228 CHB	1.401	1282
2 (-)	Lactobacillus plantarum DSM 20205 DSM	1.339	1590
3 (-)	Corynebacterium kroppenstedtii DSM 44385T DSM	1.333	161879
4 (-)	Lactobacillus salivarius DSM 20492 DSM	1.311	1624
5 (-)	Enterococcus faecium 11037 CHB	1.309	1352
6 (-)	Lactobacillus paracasei ssp paracasei DSM 20207 DSM	1.296	47714
7 (-)	Paenibacillus kobensis DSM 10249T DSM	1.289	59841
8 (-)	Candida viswanathii CBS 1924 CBS	1.285	5486
9 (-)	Staphylococcus lugdunensis DSM 4806 DSM	1.27	28035
10 (-)	Bacillus aquimaris DSM 16205T DSM	1.269	189382

Analyte165

Analyte Name:	G21
Analyte Description:	
Analyte ID:	CN21A
Analyte Creation Date/Time:	2019-12-04T13:54:31.977
Applied MSP Library(ies):	BDAL, Filamentous Fungi Library 1.0, Mycobacteria Library 1.0 (bead method), IVD, Listeria
Applied Taxonomy Tree:	

Rank (Quality)	Matched Pattern	Score Value	NCBI Identifier
1 (-)	Rothia dentocariosa CCUG 29965 CCUG	1.294	2047
2 (-)	Lactobacillus paracasei ssp paracasei DSM 20207 DSM	1.273	47714
3 (-)	Weissella viridescens DSM 20248 DSM	1.246	1629
4 (-)	Streptococcus salivarius 0807M25049501 IBS	1.228	1304
5 (-)	Weissella halotolerans DSM 20190T DSM	1.2	1615
6 (-)	Pichia occidentalis CBS 1910 CBS	1.199	54552
7 (-)	Paenibacillus macerans DSM 1574 DSM	1.195	44252
8 (-)	Burkholderia fungorum LMG 20227T HAM	1.183	134537
9 (-)	Neisseria meningitidis 639 PGM	1.174	487
10 (-)	Plesiomonas shigelloides CCM 1996 CCM	1.173	703

Analyte166

Analyte Name:	G22
Analyte Description:	
Analyte ID:	CN21A
Analyte Creation Date/Time:	2019-12-04T13:54:32.534
Applied MSP Library(ies):	BDAL, Filamentous Fungi Library 1.0, Mycobacteria Library 1.0 (bead method), IVD, Listeria
Applied Taxonomy Tree:	

Rank (Quality)	Matched Pattern	Score Value	NCBI Identifier
1 (+)	Rothia mucilaginosa BK2995_09 ERL	1.804	43675
2 (+)	Rothia mucilaginosa CCUG 44966 CCUG	1.732	43675
3 (+)	Rothia dentocariosa CCUG 29965 CCUG	1.719	2047
4 (-)	Rothia mucilaginosa CCUG 52532 CCUG	1.678	43675
5 (-)	Rothia mucilaginosa DSM 20746T DSM	1.666	43675
6 (-)	Rothia dentocariosa RV_BA1_032010_D LBK	1.617	2047
7 (-)	Rothia mucilaginosa DSM 20446 BRB	1.589	43675
8 (-)	Rothia mucilaginosa CCUG 31189 CCUG	1.558	43675
9 (-)	Rothia dentocariosa DSM 43762T DSM	1.496	2047
10 (-)	Streptococcus vestibularis CCUG 51352 CCUG	1.419	1343

Analyte167

Analyte Name:	G23
Analyte Description:	
Analyte ID:	CN21B
Analyte Creation Date/Time:	2019-12-04T13:54:31.860
Applied MSP Library(ies):	BDAL, Filamentous Fungi Library 1.0, Mycobacteria Library 1.0 (bead method), IVD, Listeria
Applied Taxonomy Tree:	

Rank (Quality)	Matched Pattern	Score Value	NCBI Identifier
1 (++)	Rothia dentocariosa RV_BA1_032010_D LBK	2.095	2047
2 (++)	Rothia dentocariosa CCUG 29965 CCUG	2.033	2047
3 (+)	Rothia dentocariosa G6496_ch28 IBS	1.874	2047
4 (+)	Rothia dentocariosa B16575_bh8 IBS	1.783	2047
5 (+)	Rothia dentocariosa DSM 43762T DSM	1.728	2047
6 (-)	Rothia dentocariosa G18709 IBS	1.594	2047
7 (-)	Rothia mucilaginosa CCUG 52532 CCUG	1.484	43675
8 (-)	Arthrobacter stackebrandtii DSM 16005T DSM	1.471	272161
9 (-)	Rothia mucilaginosa CCUG 44966 CCUG	1.448	43675
10 (-)	Streptococcus salivarius ssp salivarius 140417_01 ETL	1.427	1304

Analyte168

Analyte Name:	G24
Analyte Description:	
Analyte ID:	CN21B
Analyte Creation Date/Time:	2019-12-04T13:54:32.236
Applied MSP Library(ies):	BDAL, Filamentous Fungi Library 1.0, Mycobacteria Library 1.0 (bead method), IVD, Listeria
Applied Taxonomy Tree:	

Rank (Quality)	Matched Pattern	Score Value	NCBI Identifier
1 (-)	Thauera chlorobenzoica 3CB_1 MPB	1.556	96773
2 (-)	Lactobacillus curvatus DSM 20010 DSM	1.466	28038
3 (-)	Rothia dentocariosa CCUG 29965 CCUG	1.448	2047
4 (-)	Actinocorallia libanotica B246 UFL	1.443	46162
5 (-)	Clostridium baratii 1018_NCTC 10986 BOG	1.439	1561
6 (-)	Lactobacillus curvatus DSM 20019T DSM	1.438	28038
7 (-)	Cellulomonas gelida IMET 11078 HKJ	1.367	1712
8 (-)	Streptococcus sanguinis DSM 20567T DSM	1.337	1305
9 (-)	Lactobacillus fermentum DSM 20391 DSM	1.327	1613
10 (-)	Halomonas elongata B558 UFL	1.322	2746

Analyte169

Analyte Name:	H1
Analyte Description:	
Analyte ID:	CN21C
Analyte Creation Date/Time:	2019-12-04T13:54:32.134
Applied MSP Library(ies):	BDAL, Filamentous Fungi Library 1.0, Mycobacteria Library 1.0 (bead method), IVD, Listeria
Applied Taxonomy Tree:	

Rank (Quality)	Matched Pattern	Score Value	NCBI Identifier
1 (-)	Streptococcus vestibularis CCUG 51352 CCUG	1.56	1343
2 (-)	Streptococcus salivarius 0807M25049501 IBS	1.482	1304
3 (-)	Streptococcus sobrinus DSM 20742T DSM	1.42	1310
4 (-)	Streptococcus pneumoniae besSt29 THL	1.41	1313
5 (-)	Streptococcus equi_ssp_zooepidemicus ATCC 43079T THL	1.396	40041
6 (-)	Streptococcus gordonii DSM 6777T DSM	1.394	1302
7 (-)	Lactobacillus graminis DSM 20719T DSM	1.391	60519
8 (-)	Nocardioides jensenii DSM 20641T DSM	1.381	1843
9 (-)	Streptococcus orisratti DSM 15617T DSM	1.376	114652
10 (-)	Pseudomonas thermotolerans DSM 14292T HAM	1.345	157784

Analyte170

Analyte Name:	H2
Analyte Description:	
Analyte ID:	CN21C
Analyte Creation Date/Time:	2019-12-04T13:54:32.208
Applied MSP Library(ies):	BDAL, Filamentous Fungi Library 1.0, Mycobacteria Library 1.0 (bead method), IVD, Listeria
Applied Taxonomy Tree:	

Rank (Quality)	Matched Pattern	Score Value	NCBI Identifier
1 (-)	Streptococcus salivarius 0807M25049501 IBS	1.699	1304
2 (-)	Streptococcus downei DSM 5635T DSM	1.534	1317
3 (-)	Rothia dentocariosa DSM 43762T DSM	1.524	2047
4 (-)	Corynebacterium urealyticum DSM 7110 DSM	1.442	43771
5 (-)	Streptococcus gordonii DSM 6777T DSM	1.427	1302
6 (-)	Rothia dentocariosa RV_BA1_032010_D LBK	1.421	2047
7 (-)	Rothia dentocariosa CCUG 29965 CCUG	1.409	2047
8 (-)	Streptococcus equi_ssp_ruminatorum DSM 17037T DSM	1.399	254358
9 (-)	Rothia dentocariosa B16575_bh8 IBS	1.368	2047
10 (-)	Lactobacillus graminis DSM 20719T DSM	1.314	60519

Analyte171

Analyte Name:	H3
Analyte Description:	
Analyte ID:	CN2A
Analyte Creation Date/Time:	2019-12-04T13:54:32.046
Applied MSP Library(ies):	Listeria, IVD, Mycobacteria Library 1.0 (bead method), Filamentous Fungi Library 1.0, BDAL
Applied Taxonomy Tree:	

Rank (Quality)	Matched Pattern	Score Value	NCBI Identifier
1 (-)	Staphylococcus epidermidis DSM 1798 DSM	1.391	1282
2 (-)	Paenibacillus stellifer DSM 14472T DSM	1.331	169760
3 (-)	Enterococcus faecalis ATCC 29212 CHB	1.288	1351
4 (-)	Lactobacillus sakei ssp sakei DSM 20017T DSM	1.273	214326
5 (-)	Aeromonas veronii CECT 4199T DSM	1.27	654
6 (-)	Mycoplasma bovis 86B96 VLW	1.264	28903
7 (-)	Paenibacillus apiarius DSM 5582 DSM	1.263	46240
8 (-)	Burkholderia thailandensis DSM 13276T HAM	1.243	57975
9 (-)	Rothia aeria DSM 14556T DSM	1.22	172042
10 (-)	Pseudomonas savastanoi ssp savastanoi LMG 5011 HAM	1.21	29438

Analyte172

Analyte Name:	H4
Analyte Description:	
Analyte ID:	CN2A
Analyte Creation Date/Time:	2019-12-04T13:54:32.446
Applied MSP Library(ies):	BDAL, Filamentous Fungi Library 1.0, Mycobacteria Library 1.0 (bead method), IVD, Listeria
Applied Taxonomy Tree:	

Rank (Quality)	Matched Pattern	Score Value	NCBI Identifier
1 (-)	Lactobacillus saerimneri DSM 16049T DSM	1.362	228229
2 (-)	Weissella viridescens DSM 20248 DSM	1.356	1629
3 (-)	Rothia aeria DSM 14556T DSM	1.273	172042
4 (-)	Lactobacillus saerimneri DSM 16027 DSM	1.26	228229
5 (-)	Paenibacillus thiaminolyticus DSM 5713 DSM	1.205	49283
6 (-)	Ralstonia sp B484 UFL	1.205	48736
7 (-)	Lactobacillus versmoldensis DSM 14857T DSM	1.203	194326
8 (-)	Paenibacillus alvei DSM 5560 DSM	1.199	44250
9 (-)	Clostridium sardiniense 1001_NCTC 10984T BOG	1.177	29369
10 (-)	Actinocorallia libanotica B246 UFL	1.169	46162

Analyte173

Analyte Name:	H5
Analyte Description:	
Analyte ID:	CN2B
Analyte Creation Date/Time:	2019-12-04T13:54:32.009
Applied MSP Library(ies):	BDAL, Filamentous Fungi Library 1.0, Mycobacteria Library 1.0 (bead method), IVD, Listeria
Applied Taxonomy Tree:	

Rank (Quality)	Matched Pattern	Score Value	NCBI Identifier
1 (-)	Rothia mucilaginosa BK2995_09 ERL	1.54	43675
2 (-)	Rothia mucilaginosa CCUG 44966 CCUG	1.436	43675
3 (-)	Rothia aeria CCUG 50760 CCUG	1.429	172042
4 (-)	Rothia mucilaginosa DSM 20446 BRB	1.347	43675
5 (-)	Aeromonas hydrophila CECT 839T DSM	1.322	644
6 (-)	Rothia mucilaginosa DSM 20445 DSM	1.319	43675
7 (-)	Rothia mucilaginosa CCUG 52532 CCUG	1.294	43675
8 (-)	Arthrobacter oxydans DSM 20119T DSM	1.264	1671
9 (-)	Pseudomonas mandelii CIP 105273T HAM	1.255	75612
10 (-)	Rothia mucilaginosa CCUG 31189 CCUG	1.255	43675

Analyte174

Analyte Name:	H6
Analyte Description:	
Analyte ID:	CN2B
Analyte Creation Date/Time:	2019-12-04T13:54:32.325
Applied MSP Library(ies):	Listeria, BDAL, Filamentous Fungi Library 1.0, Mycobacteria Library 1.0 (bead method), IVD
Applied Taxonomy Tree:	

Rank (Quality)	Matched Pattern	Score Value	NCBI Identifier
1 (+)	Rothia mucilaginosa CCUG 44966 CCUG	1.8	43675
2 (-)	Rothia mucilaginosa BK2995_09 ERL	1.693	43675
3 (-)	Rothia mucilaginosa CCUG 31189 CCUG	1.664	43675
4 (-)	Rothia mucilaginosa CCUG 52532 CCUG	1.616	43675
5 (-)	Rothia mucilaginosa DSM 20446 BRB	1.615	43675
6 (-)	Rothia aeria DSM 14556T DSM	1.488	172042
7 (-)	Rothia dentocariosa DSM 43762T DSM	1.477	2047
8 (-)	Propionibacterium thoenii DSM 20276T DSM	1.465	1751
9 (-)	Clostridium cochlearium 1080_ATCC 17794T BOG	1.455	1494
10 (-)	Propionibacterium thoenii DSM 20277 DSM	1.395	1751

Analyte175

Analyte Name:	H7
Analyte Description:	
Analyte ID:	CN2C
Analyte Creation Date/Time:	2019-12-04T13:54:32.664
Applied MSP Library(ies):	IVD, Mycobacteria Library 1.0 (bead method), Filamentous Fungi Library 1.0, BDAL, Listeria
Applied Taxonomy Tree:	

Rank (Quality)	Matched Pattern	Score Value	NCBI Identifier
1 (-)	Neisseria mucosa DSM 17611T DSM	1.695	488
2 (-)	Neisseria subflava DSM 17610T DSM_2	1.42	28449
3 (-)	Achromobacter spanius LMG 5911T HAM	1.243	217203
4 (-)	Sphingomonas trueperi DSM 7225T DSM	1.206	53317
5 (-)	Rhizobium radiobacter 994000124 LBK	1.199	358
6 (-)	Aeromonas hydrophila CECT 839T DSM	1.18	644
7 (-)	Arthrobacter arilaitensis DSM 16368T DSM	1.174	256701
8 (-)	Bacillus horikoshii DSM 8719T DSM	1.174	79883
9 (-)	Lactobacillus sharpeae DSM 20507 DSM	1.169	1626
10 (-)	Neisseria meningitidis Serogroup_A BRL	1.163	487

Analyte176

Analyte Name:	H8
Analyte Description:	
Analyte ID:	CN2C
Analyte Creation Date/Time:	2019-12-04T13:54:32.379
Applied MSP Library(ies):	BDAL, Filamentous Fungi Library 1.0, Mycobacteria Library 1.0 (bead method), IVD, Listeria
Applied Taxonomy Tree:	

Rank (Quality)	Matched Pattern	Score Value	NCBI Identifier
1 (-)	Cupriavidus necator B619 UFL	1.269	106590
2 (-)	Cellulomonas gelida IMET 11078 HKJ	1.229	1712
3 (-)	Clostridium clostridioforme 1021_NCTC 11224T BOG	1.212	1531
4 (-)	Lactobacillus sharpeae DSM 20506 DSM	1.207	1626
5 (-)	Enterococcus canintestini P_3828_1 IMK	1.19	317010
6 (-)	Lactobacillus sharpeae DSM 20507 DSM	1.183	1626
7 (-)	Streptococcus vestibularis DSM 5636T DSM	1.171	1343
8 (-)	Blastomonas ursincola DSM 9006T HAM	1.16	56361
9 (-)	Lactobacillus reuteri DSM 20053 DSM	1.153	1598
10 (-)	Tissierella praeacuta 1078_ATCC 33268T BOG	1.15	43131

Analyte177

Analyte Name:	H9
Analyte Description:	
Analyte ID:	CN25A
Analyte Creation Date/Time:	2019-12-04T13:54:32.086
Applied MSP Library(ies):	BDAL, Filamentous Fungi Library 1.0, Mycobacteria Library 1.0 (bead method), IVD, Listeria
Applied Taxonomy Tree:	

Rank (Quality)	Matched Pattern	Score Value	NCBI Identifier
1 (+)	Streptococcus salivarius 0807M25049501 IBS	1.827	1304
2 (-)	Streptococcus suis DSM 9682T DSM	1.452	1307
3 (-)	Lactobacillus sharpeae DSM 20505T DSM	1.401	1626
4 (-)	Lactobacillus aviarius ssp aviarius DSM 20654 DSM	1.37	147810
5 (-)	Lactobacillus coryniformis ssp torquens DSM 20004T DSM	1.367	115542
6 (-)	Clostridium cadaveris 1074_ATCC 25783T BOG	1.342	1529
7 (-)	Lactobacillus sharpeae DSM 20504 DSM	1.336	1626
8 (-)	Streptococcus australis DSM 15627T DSM	1.331	113107
9 (-)	Neisseria meningitidis C1 2 PGM	1.317	487
10 (-)	Penicillium digitatum DSM 2732 DSM	1.308	123269315

Analyte178

Analyte Name:	H10
Analyte Description:	
Analyte ID:	CN25A
Analyte Creation Date/Time:	2019-12-04T13:54:32.457
Applied MSP Library(ies):	BDAL, Filamentous Fungi Library 1.0, Mycobacteria Library 1.0 (bead method), IVD, Listeria
Applied Taxonomy Tree:	

Rank (Quality)	Matched Pattern	Score Value	NCBI Identifier
1 (-)	Streptococcus salivarius IBS_MS_23 IBS	1.532	1304
2 (-)	Streptococcus sobrinus DSM 20742T DSM	1.428	1310
3 (-)	Lactobacillus sharpeae DSM 20506 DSM	1.385	1626
4 (-)	Streptococcus salivarius 0807M25049501 IBS	1.336	1304
5 (-)	Rhizobium radiobacter B178 UFL	1.314	358
6 (-)	Brevibacterium casei IMET 10997T HKJ	1.294	33889
7 (-)	Lactobacillus sharpeae DSM 20505T DSM	1.29	1626
8 (-)	Streptococcus suis ISU 29164 ISUV	1.276	1307
9 (-)	Lactobacillus sharpeae DSM 20504 DSM	1.276	1626
10 (-)	Lactobacillus gastricus DSM 16046 DSM	1.263	227942

Analyte179

Analyte Name:	H11
Analyte Description:	
Analyte ID:	CN25B
Analyte Creation Date/Time:	2019-12-04T13:54:31.663
Applied MSP Library(ies):	BDAL, Filamentous Fungi Library 1.0, Mycobacteria Library 1.0 (bead method), IVD, Listeria
Applied Taxonomy Tree:	

Rank (Quality)	Matched Pattern	Score Value	NCBI Identifier
1 (+)	Streptococcus salivarius 0807M25049501 IBS	1.861	1304
2 (+)	Streptococcus salivarius DSM 20560T DSM	1.793	1304
3 (-)	Streptococcus macacae DSM 20724T DSM	1.552	1339
4 (-)	Rothia dentocariosa DSM 43762T DSM	1.475	2047
5 (-)	Lactobacillus sharpeae DSM 20505T DSM	1.428	1626
6 (-)	Streptococcus downei DSM 5635T DSM	1.389	1317
7 (-)	Streptococcus salivarius_ssp_thermophilus DSM 8713 DSM	1.369	1304
8 (-)	Streptococcus phocae DSM 15635T BRB	1.367	119224
9 (-)	Brevibacillus borstelensis 5_5 TUB	1.31	45462
10 (-)	Weissella viridescens DSM 20248 DSM	1.309	1629

Analyte180

Analyte Name:	H12
Analyte Description:	
Analyte ID:	CN25B
Analyte Creation Date/Time:	2019-12-04T13:54:31.732
Applied MSP Library(ies):	BDAL, Filamentous Fungi Library 1.0, Mycobacteria Library 1.0 (bead method), IVD, Listeria
Applied Taxonomy Tree:	

Rank (Quality)	Matched Pattern	Score Value	NCBI Identifier
1 (+)	Streptococcus salivarius 0807M25049501 IBS	1.981	1304
2 (-)	Rothia dentocariosa DSM 43762T DSM	1.592	2047
3 (-)	Streptococcus salivarius DSM 20560T DSM	1.572	1304
4 (-)	Streptococcus vestibularis CCUG 51352 CCUG	1.495	1343
5 (-)	Rothia dentocariosa G6496_ch28 IBS	1.443	2047
6 (-)	Rothia dentocariosa B16575_bh8 IBS	1.437	2047
7 (-)	Streptococcus vestibularis 14147704_2 MVD	1.414	1343
8 (-)	Streptococcus vestibularis CCUG 61229 CCUG	1.384	1343
9 (-)	Streptococcus macacae DSM 20724T DSM	1.383	1339
10 (-)	Streptococcus downei DSM 5635T DSM	1.37	1317

Analyte181

Analyte Name:	H13
Analyte Description:	
Analyte ID:	C33
Analyte Creation Date/Time:	2019-12-04T13:54:31.897
Applied MSP Library(ies):	BDAL, Filamentous Fungi Library 1.0, Mycobacteria Library 1.0 (bead method), IVD, Listeria
Applied Taxonomy Tree:	

Rank (Quality)	Matched Pattern	Score Value	NCBI Identifier
1 (+)	Streptococcus salivarius 0807M25049501 IBS	1.765	1304
2 (-)	Streptococcus salivarius_ssp_thermophilus DSM 8713 DSM	1.603	1304
3 (-)	Streptococcus orisratti DSM 15617T DSM	1.563	114652
4 (-)	Lactobacillus sharpeae DSM 20505T DSM	1.514	1626
5 (-)	Streptococcus vestibularis 14147704_2 MVD	1.46	1343
6 (-)	Streptococcus salivarius DSM 20560T BRB	1.4	1304
7 (-)	Streptococcus downei DSM 5635T DSM	1.397	1317
8 (-)	Streptococcus vestibularis CCUG 51352 CCUG	1.356	1343
9 (-)	Streptococcus hyovaginalis DSM 12220 DSM	1.342	149015
10 (-)	Chryseobacterium scophthalmum LMG 13028T HAM	1.337	59733

Analyte182

Analyte Name:	H14
Analyte Description:	
Analyte ID:	C33
Analyte Creation Date/Time:	2019-12-04T13:54:31.790
Applied MSP Library(ies):	BDAL, Filamentous Fungi Library 1.0, Mycobacteria Library 1.0 (bead method), IVD, Listeria
Applied Taxonomy Tree:	

Rank (Quality)	Matched Pattern	Score Value	NCBI Identifier
1 (+)	Streptococcus salivarius 0807M25049501 IBS	1.836	1304
2 (+)	Streptococcus salivarius DSM 20560T DSM	1.772	1304
3 (-)	Streptococcus downei DSM 5635T DSM	1.56	1317
4 (-)	Streptococcus salivarius IBS_MS_23 IBS	1.409	1304
5 (-)	Lactobacillus sharpeae DSM 20505T DSM	1.401	1626
6 (-)	Streptococcus orisratti DSM 15617T DSM	1.396	114652
7 (-)	Streptococcus gordonii DSM 6777T DSM	1.395	1302
8 (-)	Streptococcus parasanguinis CCUG 55521 CCUG	1.346	1318
9 (-)	Streptococcus equi_ssp_zooepidemicus DSM 20727T DSM	1.325	40041
10 (-)	Streptococcus equi_ssp_ruminatorum DSM 17037T DSM	1.311	254358

Analyte183

Analyte Name:	H15
Analyte Description:	
Analyte ID:	CN23A
Analyte Creation Date/Time:	2019-12-04T13:54:32.567
Applied MSP Library(ies):	BDAL, Filamentous Fungi Library 1.0, Mycobacteria Library 1.0 (bead method), IVD, Listeria
Applied Taxonomy Tree:	

Rank (Quality)	Matched Pattern	Score Value	NCBI Identifier
1 (-)	Neisseria mucosa DSM 17611T DSM	1.555	488
2 (-)	Neisseria meningitidis C1 2 PGM	1.437	487
3 (-)	Lactobacillus reuteri DSM 20053 DSM	1.391	1598
4 (-)	Neisseria elongata ssp elongata DSM 17712T DSM	1.391	214482
5 (-)	Neisseria subflava 1672 PGM	1.39	28449
6 (-)	Clostridium aciditolerans DSM 17425T DSM	1.389	339861
7 (-)	Neisseria meningitidis 24086406 MLD	1.324	487
8 (-)	Blastomonas natatoria DSM 3183T HAM	1.303	34015
9 (-)	Neisseria flavescens 533 PGM	1.275	484
10 (-)	Neisseria gonorrhoeae ps601 PGM	1.262	485

Analyte184

Analyte Name:	H16
Analyte Description:	
Analyte ID:	CN23A
Analyte Creation Date/Time:	2019-12-04T13:54:31.937
Applied MSP Library(ies):	BDAL, Filamentous Fungi Library 1.0, Mycobacteria Library 1.0 (bead method), IVD, Listeria
Applied Taxonomy Tree:	

Rank (Quality)	Matched Pattern	Score Value	NCBI Identifier
1 (-)	Blastomonas natatoria DSM 3183T HAM	1.381	34015
2 (-)	Neisseria flavescens C1 2 PGM	1.373	484
3 (-)	Neisseria mucosa DSM 17611T DSM	1.329	488
4 (-)	Neisseria sicca 110 PGM	1.323	490
5 (-)	Clostridium bifermentans 1027_NCTC 1341 BOG	1.295	1490
6 (-)	Neisseria meningitidis C1 2 PGM	1.243	487
7 (-)	Neisseria sicca 70 PGM	1.233	490
8 (-)	Neisseria gonorrhoeae ps601 PGM	1.227	485
9 (-)	Streptococcus alactolyticus DSM 5199 DSM	1.226	29389
10 (-)	Aeromonas schubertii CECT 4240T DSM	1.217	652

Analyte185

Analyte Name:	H17
Analyte Description:	
Analyte ID:	CN23B
Analyte Creation Date/Time:	2019-12-04T13:54:32.396
Applied MSP Library(ies):	BDAL, Filamentous Fungi Library 1.0, Mycobacteria Library 1.0 (bead method), IVD, Listeria
Applied Taxonomy Tree:	

Rank (Quality)	Matched Pattern	Score Value	NCBI Identifier
1 (-)	Staphylococcus epidermidis DSM 1798 DSM	1.448	1282
2 (-)	Clostridium baratii 1018_NCTC 10986 BOG	1.432	1561
3 (-)	Arthrobacter roseus DSM 14508T DSM	1.424	136274
4 (-)	Agromyces italicus HKI 325_DSM 16388T HKJ	1.378	279572
5 (-)	Cryptococcus neoformans_var_grubii ICB175_SDA_NaCl CBS	1.351	178876
6 (-)	Halomonas elongata B496 UFL	1.346	2746
7 (-)	Bacillus cereus DSM 31T DSM	1.339	1396
8 (-)	Staphylococcus simulans DSM 20324 DSM	1.325	1286
9 (-)	Enterococcus faecium VRE_PX_16086218 MLD	1.316	1352
10 (-)	Bacillus muralis DSM 16288T DSM	1.313	264697

Analyte186

Analyte Name:	H18
Analyte Description:	
Analyte ID:	CN23B
Analyte Creation Date/Time:	2019-12-04T13:54:32.384
Applied MSP Library(ies):	BDAL, Filamentous Fungi Library 1.0, Mycobacteria Library 1.0 (bead method), IVD, Listeria
Applied Taxonomy Tree:	

Rank (Quality)	Matched Pattern	Score Value	NCBI Identifier
1 (-)	Bacillus cereus DSM 31T DSM	1.444	1396
2 (-)	Bacillus pseudomycoides DSM 12442T DSM	1.439	64104
3 (-)	Staphylococcus epidermidis ATCC 14990T THL	1.367	1282
4 (-)	Candida parapsilosis ATCC 22019 THL	1.364	5480
5 (-)	Staphylococcus capitis ssp capitis DSM 6180 DSM	1.359	72758
6 (-)	Staphylococcus simulans DSM 20324 DSM	1.353	1286
7 (-)	Staphylococcus epidermidis DSM 1798 DSM	1.313	1282
8 (-)	Paenibacillus brasilensis DSM 14914T DSM	1.292	128574
9 (-)	Vibrio brasiliensis DSM 17184T HAM	1.286	170652
10 (-)	Lactobacillus plantarum DSM 12028 DSM	1.262	1590

Analyte187

Analyte Name:	H19
Analyte Description:	
Analyte ID:	CN6A
Analyte Creation Date/Time:	2019-12-04T13:54:32.241
Applied MSP Library(ies):	BDAL, Filamentous Fungi Library 1.0, Mycobacteria Library 1.0 (bead method), IVD, Listeria
Applied Taxonomy Tree:	

Rank (Quality)	Matched Pattern	Score Value	NCBI Identifier
1 (+)	Rothia aeria CCUG 50760 CCUG	1.868	172042
2 (-)	Rothia aeria 120619_15_b HUA	1.58	172042
3 (-)	Streptococcus salivarius 0807M25049501 IBS	1.569	1304
4 (-)	Aeromonas molluscorum 848T DSM	1.541	271417
5 (-)	Shewanella baltica DSM 9439T HAM	1.516	62322
6 (-)	Streptococcus salivarius DSM 20560T DSM	1.432	1304
7 (-)	Burkholderia phymatum LMG 21445T HAM	1.376	148447
8 (-)	Streptococcus salivarius ssp salivarius 140417_01 ETL	1.337	1304
9 (-)	Paenibacillus massiliensis DSM 16942T DSM	1.323	225917
10 (-)	Chryseobacterium scophthalmum LMG 13028T HAM	1.321	59733

Analyte188

Analyte Name:	H20
Analyte Description:	
Analyte ID:	CN6A
Analyte Creation Date/Time:	2019-12-04T13:54:32.558
Applied MSP Library(ies):	BDAL, Filamentous Fungi Library 1.0, Mycobacteria Library 1.0 (bead method), IVD, Listeria
Applied Taxonomy Tree:	

Rank (Quality)	Matched Pattern	Score Value	NCBI Identifier
1 (-)	Staphylococcus epidermidis 6b_s ESL	1.496	1282
2 (-)	Streptococcus salivarius DSM 20560T DSM	1.49	1304
3 (-)	Lactobacillus paracasei ssp paracasei DSM 8741 DSM	1.423	47714
4 (-)	Agromyces italicus HKI 325_DSM 16388T HKJ	1.391	279572
5 (-)	Streptococcus salivarius 0807M25049501 IBS	1.387	1304
6 (-)	Staphylococcus epidermidis 4b_r ESL	1.373	1282
7 (-)	Bacillus farraginis DSM 16013T DSM	1.343	254757
8 (-)	Rothia aeria Cory_26 IBS	1.325	172042
9 (-)	Rothia aeria 120619_15_b HUA	1.3	172042
10 (-)	Mycobacterium tuberculosis AI36 R_899 PGM	1.294	1773

Analyte189

Analyte Name:	H21
Analyte Description:	
Analyte ID:	CN6B
Analyte Creation Date/Time:	2019-12-04T13:54:32.646
Applied MSP Library(ies):	BDAL, Filamentous Fungi Library 1.0, Mycobacteria Library 1.0 (bead method), IVD, Listeria
Applied Taxonomy Tree:	

Rank (Quality)	Matched Pattern	Score Value	NCBI Identifier
1 (+)	Streptococcus salivarius DSM 20560T DSM	1.951	1304
2 (+)	Streptococcus salivarius 0807M25049501 IBS	1.823	1304
3 (+)	Streptococcus parasanguinis 14137939_2 MVD	1.737	1318
4 (-)	Streptococcus downei DSM 5635T DSM	1.688	1317
5 (-)	Streptococcus infantis DSM 12492T DSM	1.591	68892
6 (-)	Streptococcus oralis DSM 20627T DSM	1.584	1303
7 (-)	Streptococcus gordonii DSM 6777T DSM	1.436	1302
8 (-)	Brevibacterium casei IMET 10997T HKJ	1.433	33889
9 (-)	Streptococcus salivarius IBS_MS_23 IBS	1.43	1304
10 (-)	Chryseobacterium scophthalmum LMG 13028T HAM	1.396	59733

Analyte190

Analyte Name:	H22
Analyte Description:	
Analyte ID:	CN6B
Analyte Creation Date/Time:	2019-12-04T13:54:32.055
Applied MSP Library(ies):	BDAL, Filamentous Fungi Library 1.0, Mycobacteria Library 1.0 (bead method), IVD, Listeria
Applied Taxonomy Tree:	

Rank (Quality)	Matched Pattern	Score Value	NCBI Identifier
1 (-)	Streptococcus downei DSM 5635T DSM	1.668	1317
2 (-)	Streptococcus parasanguinis 14137939_2 MVD	1.568	1318
3 (-)	Streptococcus salivarius DSM 20560T DSM	1.497	1304
4 (-)	Streptococcus parasanguinis CCUG 55521 CCUG	1.473	1318
5 (-)	Chryseobacterium scophthalmum LMG 13028T HAM	1.415	59733
6 (-)	Streptococcus sobrinus DSM 20742T DSM	1.408	1310
7 (-)	Streptococcus orisratti DSM 15617T DSM	1.391	114652
8 (-)	Streptococcus salivarius 0807M25049501 IBS	1.367	1304
9 (-)	Streptococcus intermedius DSM 20573T DSM	1.346	1338
10 (-)	Weissella minor DSM 20014T DSM	1.336	1620

Analyte191

Analyte Name:	H23
Analyte Description:	
Analyte ID:	CN10A
Analyte Creation Date/Time:	2019-12-04T13:54:32.741
Applied MSP Library(ies):	Filamentous Fungi Library 1.0, BDAL, Mycobacteria Library 1.0 (bead method), IVD, Listeria
Applied Taxonomy Tree:	

Rank (Quality)	Matched Pattern	Score Value	NCBI Identifier
1 (-)	Actinocorallia libanotica B246 UFL	1.378	46162
2 (-)	Lactobacillus murinus DSM 20452T DSM	1.333	1622
3 (-)	Clostridium tetani 1089_ATCC 10779 BOG	1.331	1513
4 (-)	Agromyces rhizospherae HKI 302_DSM 14597T HKJ	1.319	88374
5 (-)	Neisseria meningitidis 639 PGM	1.318	487
6 (-)	Rhizobium radiobacter B177 UFL	1.309	358
7 (-)	Staphylococcus simulans DSM 20324 DSM	1.303	1286
8 (-)	Staphylococcus epidermidis ATCC 12228 CHB	1.294	1282
9 (-)	Arthrobacter citreus DSM 20133T DSM	1.284	1670
10 (-)	Weissella halotolerans DSM 20190T DSM	1.273	1615

Analyte192

Analyte Name:	H24
Analyte Description:	
Analyte ID:	CN10A
Analyte Creation Date/Time:	2019-12-04T13:54:32.347
Applied MSP Library(ies):	BDAL, Filamentous Fungi Library 1.0, Mycobacteria Library 1.0 (bead method), IVD, Listeria
Applied Taxonomy Tree:	

Rank (Quality)	Matched Pattern	Score Value	NCBI Identifier
1 (-)	Rothia aeria 120619_15_b HUA	1.631	172042
2 (-)	Rothia aeria CCUG 50760 CCUG	1.495	172042
3 (-)	Rothia aeria NO_11 HUA	1.476	172042
4 (-)	Rothia aeria CCUG 25688 CCUG	1.44	172042
5 (-)	Agromyces bracchium HKI 303 DSM 14596T HKJ	1.423	88376
6 (-)	Halomonas elongata B582 UFL	1.396	2746
7 (-)	Rothia aeria Cory_26 IBS	1.391	172042
8 (-)	Blastomonas ursincola DSM 9006T HAM	1.359	56361
9 (-)	Actinocorallia libanotica B246 UFL	1.356	46162
10 (-)	Rothia aeria DSM 14556T DSM	1.334	172042

Analyte193

Analyte Name:	I1
Analyte Description:	
Analyte ID:	CN10B
Analyte Creation Date/Time:	2019-12-04T13:54:32.065
Applied MSP Library(ies):	BDAL, Filamentous Fungi Library 1.0, Mycobacteria Library 1.0 (bead method), IVD, Listeria
Applied Taxonomy Tree:	

Rank (Quality)	Matched Pattern	Score Value	NCBI Identifier
1 (-)	Acinetobacter baumannii B389 UFL	1.528	470
2 (-)	Pichia occidentalis CBS 1910 CBS	1.519	54552
3 (-)	Colletotrichum gloeosporioides CBS 100471 CBS	1.441	474922
4 (-)	Streptococcus pneumoniae besSt29 THL	1.417	1313
5 (-)	Herbaspirillum frisingense DSM 13128T DSM	1.403	92645
6 (-)	Filifactor villosus 1051_NCTC 11220T BOG	1.394	29374
7 (-)	Staphylococcus simulans DSM 20324 DSM	1.365	1286
8 (-)	Arthrobacter ramosus IMET 10685T HKJ	1.318	1672
9 (-)	Enterococcus faecium VRE_PX_16086218 MLD	1.301	1352
10 (-)	Lactobacillus fuchuensis DSM 14340T DSM	1.287	164393

Analyte194

Analyte Name:	I2
Analyte Description:	
Analyte ID:	CN10B
Analyte Creation Date/Time:	2019-12-04T13:54:32.256
Applied MSP Library(ies):	BDAL, Filamentous Fungi Library 1.0, Mycobacteria Library 1.0 (bead method), IVD, Listeria
Applied Taxonomy Tree:	

Rank (Quality)	Matched Pattern	Score Value	NCBI Identifier
1 (+)	Streptococcus salivarius 0807M25049501 IBS	1.731	1304
2 (-)	Rothia dentocariosa DSM 43762T DSM	1.636	2047
3 (-)	Streptococcus salivarius DSM 20560T DSM	1.633	1304
4 (-)	Streptococcus macacae DSM 20724T DSM	1.518	1339
5 (-)	Streptococcus salivarius IBS_MS_23 IBS	1.509	1304
6 (-)	Rothia dentocariosa CCUG 29965 CCUG	1.504	2047
7 (-)	Rothia mucilaginosa DSM 20446 DSM	1.458	43675
8 (-)	Rothia mucilaginosa CCUG 44966 CCUG	1.38	43675
9 (-)	Rothia mucilaginosa BK2995_09 ERL	1.38	43675
10 (-)	Streptococcus vestibularis CCUG 61229 CCUG	1.339	1343

Analyte195

Analyte Name:	I3
Analyte Description:	
Analyte ID:	CN31
Analyte Creation Date/Time:	2019-12-04T13:54:32.282
Applied MSP Library(ies):	BDAL, Filamentous Fungi Library 1.0, Mycobacteria Library 1.0 (bead method), IVD, Listeria
Applied Taxonomy Tree:	

Rank (Quality)	Matched Pattern	Score Value	NCBI Identifier
1 (-)	Enterococcus faecium VRE_PX_16086218 MLD	1.464	1352
2 (-)	Lactobacillus satsumensis DSM 16230T DSM	1.399	259059
3 (-)	Lactobacillus pantheris DSM 15945T DSM	1.393	171523
4 (-)	Lactobacillus antri DSM 16041T DSM	1.384	227943
5 (-)	Propionibacterium acnes DSM 1897T DSM	1.367	1747
6 (-)	Kandleria vitulina DSM 20405T DSM	1.356	1630
7 (-)	Paracoccus versutus B352 UFL	1.355	34007
8 (-)	Lactobacillus crispatus DSM 20584T DSM	1.33	47770
9 (-)	Filifactor villosus 1051_NCTC 11220T BOG	1.324	29374
10 (-)	Streptococcus salivarius DSM 20067 DSM	1.318	1304

Analyte196

Analyte Name:	I4
Analyte Description:	
Analyte ID:	CN31
Analyte Creation Date/Time:	2019-12-04T13:54:31.830
Applied MSP Library(ies):	BDAL, Filamentous Fungi Library 1.0, Mycobacteria Library 1.0 (bead method), IVD, Listeria
Applied Taxonomy Tree:	

Rank (Quality)	Matched Pattern	Score Value	NCBI Identifier
1 (-)	Lactobacillus brevis DSM 1267 DSM	1.456	1580
2 (-)	Rhizopus stolonifer 111116_03 IMD	1.425	123269315
3 (-)	Streptococcus downei DSM 5635T DSM	1.369	1317
4 (-)	Lactobacillus brevis DSM 1268 DSM	1.368	1580
5 (-)	Neisseria weaveri DSM 17688T DSM	1.326	28091
6 (-)	Lactobacillus brevis DSM 2647 DSM	1.324	1580
7 (-)	Staphylococcus simulans DSM 20723 DSM	1.311	1286
8 (-)	Staphylococcus aureus ssp aureus DSM 3463 DSM	1.305	46170
9 (-)	Lactobacillus vaccinostercus DSM 15802T DSM	1.296	176291
10 (-)	Lactobacillus intestinalis DSM 6629T DSM	1.29	151781

Analyte197

Analyte Name: I5
Analyte Description:
Analyte ID: CN11A
Analyte Creation Date/Time: 2019-12-04T13:54:32.129
Applied MSP Library(ies): BDAL, Filamentous Fungi Library 1.0, Mycobacteria Library 1.0 (bead method), IVD, Listeria
Applied Taxonomy Tree:

Rank (Quality)	Matched Pattern	Score Value	NCBI Identifier
1 (-)	Staphylococcus aureus ssp aureus DSM 3463 DSM	1.542	46170
2 (-)	Staphylococcus aureus ssp aureus DSM 20231T DSM	1.442	46170
3 (-)	Staphylococcus aureus ssp anaerobius DSM 20714T DSM	1.412	72759
4 (-)	Lactobacillus paracasei ssp paracasei DSM 8741 DSM	1.384	47714
5 (-)	Lactobacillus agilis DSM 20509T DSM	1.363	1601
6 (-)	Lactobacillus fermentum DSM 20391 DSM	1.347	1613
7 (-)	Staphylococcus aureus ssp aureus DSM 799 DSM	1.346	46170
8 (-)	Clostridium novyi A 1025_NCTC 538 BOG	1.315	1542
9 (-)	Lactobacillus kimchii DSM 13961T DSM	1.29	103818
10 (-)	Actinocorallia libanotica B246 UFL	1.28	46162

Analyte198

Analyte Name:	I6
Analyte Description:	
Analyte ID:	CN11A
Analyte Creation Date/Time:	2019-12-04T13:54:31.815
Applied MSP Library(ies):	Listeria, IVD, Mycobacteria Library 1.0 (bead method), Filamentous Fungi Library 1.0, BDAL
Applied Taxonomy Tree:	

Rank (Quality)	Matched Pattern	Score Value	NCBI Identifier
1 (++)	Staphylococcus aureus ATCC 29213 THL	2.108	1280
2 (+)	Staphylococcus aureus ssp aureus DSM 3463 DSM	1.766	46170
3 (+)	Staphylococcus aureus ssp aureus DSM 799 DSM	1.738	46170
4 (-)	Staphylococcus aureus ssp aureus DSM 20231T DSM	1.672	46170
5 (-)	Staphylococcus aureus ATCC 33862 THL	1.574	1280
6 (-)	Staphylococcus simiae DSM 17639 DSM	1.409	308354
7 (-)	Clostridium paraputrificum 1083_ATCC 17796 BOG	1.402	29363
8 (-)	Staphylococcus xylosus FI FLR	1.392	1288
9 (-)	Staphylococcus simiae DSM 17637 DSM	1.353	308354
10 (-)	Staphylococcus aureus ssp anaerobius DSM 20714T DSM	1.33	72759

Analyte199

Analyte Name:	I7
Analyte Description:	
Analyte ID:	CN11B
Analyte Creation Date/Time:	2019-12-04T13:54:32.622
Applied MSP Library(ies):	BDAL, Filamentous Fungi Library 1.0, Mycobacteria Library 1.0 (bead method), IVD, Listeria
Applied Taxonomy Tree:	

Rank (Quality)	Matched Pattern	Score Value	NCBI Identifier
1 (-)	Rothia dentocariosa DSM 43762T DSM	1.693	2047
2 (-)	Brevibacillus centrosporus DSM 8445T DSM	1.373	54910
3 (-)	Arthrobacter citreus DSM 20133T DSM	1.298	1670
4 (-)	Rothia dentocariosa CCUG 29965 CCUG	1.289	2047
5 (-)	Lactobacillus agilis DSM 20509T DSM	1.278	1601
6 (-)	Lactobacillus agilis DSM 20508 DSM	1.249	1601
7 (-)	Staphylococcus lugdunensis DSM 4804T DSM	1.213	28035
8 (-)	Brevibacillus brevis DSM 30T DSM	1.191	1393
9 (-)	Paenibacillus glycanilyticus DSM 17608T DSM	1.167	126569
10 (-)	Lodderomyces elongisporus CBS 2605T CBS	1.162	36914

Analyte200

Analyte Name:	I8
Analyte Description:	
Analyte ID:	CN11B
Analyte Creation Date/Time:	2019-12-04T13:54:32.719
Applied MSP Library(ies):	BDAL, Filamentous Fungi Library 1.0, Mycobacteria Library 1.0 (bead method), IVD, Listeria
Applied Taxonomy Tree:	

Rank (Quality)	Matched Pattern	Score Value	NCBI Identifier
1 (+)	Rothia dentocariosa DSM 43762T DSM	1.811	2047
2 (-)	Bacillus megaterium DSM 32T DSM	1.235	1404
3 (-)	Rothia mucilaginosa DSM 20746T DSM	1.191	43675
4 (-)	Arthrobacter stackebrandtii DSM 16005T DSM	1.181	272161
5 (-)	Novosphingobium resinovorum DSM 10700 HAM	1.16	158500
6 (-)	Streptomyces violaceoruber B263 UFL	1.14	1935
7 (-)	Novosphingobium resinovorum DSM 7478T HAM	1.139	158500
8 (-)	Paenibacillus phyllosphaerae DSM 17399T DSM	1.128	274593
9 (-)	Lactobacillus plantarum DSM 2601 DSM	1.123	1590
10 (-)	Lactobacillus plantarum ssp plantarum DSM 20174T DSM	1.114	337330

Analyte201

Analyte Name:	I9
Analyte Description:	
Analyte ID:	CN11C
Analyte Creation Date/Time:	2019-12-04T13:54:32.172
Applied MSP Library(ies):	BDAL, Filamentous Fungi Library 1.0, Mycobacteria Library 1.0 (bead method), IVD, Listeria
Applied Taxonomy Tree:	

Rank (Quality)	Matched Pattern	Score Value	NCBI Identifier
1 (+)	Rothia dentocariosa CCUG 29965 CCUG	1.793	2047
2 (-)	Rothia dentocariosa RV_BA1_032010_D LBK	1.598	2047
3 (-)	Rothia dentocariosa G6496_ch28 IBS	1.564	2047
4 (-)	Rothia dentocariosa B16575_bh8 IBS	1.416	2047
5 (-)	Lactobacillus saerimneri DSM 16027 DSM	1.307	228229
6 (-)	Rothia mucilaginosa DSM 30548 DSM	1.285	43675
7 (-)	Aeromonas veronii CECT 4199T DSM	1.278	654
8 (-)	Lactobacillus saerimneri DSM 16049T DSM	1.27	228229
9 (-)	Stenotrophomonas acidaminiphila DSM 13117T HAM	1.265	128780
10 (-)	Arthrobacter stackebrandtii DSM 16005T DSM	1.265	272161

Analyte202

Analyte Name:	I10
Analyte Description:	
Analyte ID:	CN11C
Analyte Creation Date/Time:	2019-12-04T13:54:32.435
Applied MSP Library(ies):	Listeria, IVD, Mycobacteria Library 1.0 (bead method), Filamentous Fungi Library 1.0, BDAL
Applied Taxonomy Tree:	

Rank (Quality)	Matched Pattern	Score Value	NCBI Identifier
1 (-)	Agromyces salentinus HKI 320_DSM 16198T HKJ	1.48	269421
2 (-)	Lactobacillus agilis DSM 20509T DSM	1.405	1601
3 (-)	Clostridium bifermentans 2274_CCUG 35556 A BOG	1.342	1490
4 (-)	Streptomyces lavendulae B264 UFL	1.341	1914
5 (-)	Aspergillus flavus 1081 PFM	1.332	5059
6 (-)	Paenibacillus agaridevorans DSM 1486 DSM	1.319	171404
7 (-)	Lactobacillus paracasei ssp paracasei DSM 5622T DSM	1.308	47714
8 (-)	Empedobacter brevis LMG 4011T HAM	1.304	247
9 (-)	Mycoplasma hyorhinis FLR	1.3	2100
10 (-)	Streptomyces badius B192 UFL	1.296	1941

Analyte203

Analyte Name:	I11
Analyte Description:	
Analyte ID:	CN11D
Analyte Creation Date/Time:	2019-12-04T13:54:31.685
Applied MSP Library(ies):	BDAL, Filamentous Fungi Library 1.0, Mycobacteria Library 1.0 (bead method), IVD, Listeria
Applied Taxonomy Tree:	

Rank (Quality)	Matched Pattern	Score Value	NCBI Identifier
1 (-)	Staphylococcus lugdunensis DSM 4804T DSM	1.392	28035
2 (-)	Lactobacillus brevis DSM 1267 DSM	1.39	1580
3 (-)	Lactobacillus brevis DSM 2647 DSM	1.35	1580
4 (-)	Lactobacillus plantarum DSM 13273 DSM	1.326	1590
5 (-)	Lactobacillus hilgardii DSM 20051 DSM	1.308	1588
6 (-)	Lactobacillus brevis DSM 20054T DSM	1.28	1580
7 (-)	Lactobacillus plantarum DSM 2648 DSM	1.257	1590
8 (-)	Agromyces bracchium HKI 303 DSM 14596T HKJ	1.22	88376
9 (-)	Geotrichum sp 116 PSB	1.209	43987
10 (-)	Streptomyces lavendulae B264 UFL	1.202	1914

Analyte204

Analyte Name:	I12
Analyte Description:	
Analyte ID:	CN11D
Analyte Creation Date/Time:	2019-12-04T13:54:31.752
Applied MSP Library(ies):	BDAL, Filamentous Fungi Library 1.0, Mycobacteria Library 1.0 (bead method), IVD, Listeria
Applied Taxonomy Tree:	

Rank (Quality)	Matched Pattern	Score Value	NCBI Identifier
1 (-)	Agromyces bracchium HKI 303 DSM 14596T HKJ	1.364	88376
2 (-)	Lactobacillus plantarum DSM 13273 DSM	1.308	1590
3 (-)	Clostridium sphenoides 1046_NCTC 507T BOG	1.284	29370
4 (-)	Arthrobacter polychromogenes DSM 20136T DSM	1.24	1676
5 (-)	Staphylococcus aureus ATCC 25923 THL	1.202	1280
6 (-)	Lactobacillus acidophilus DSM 20079T DSM	1.18	1579
7 (-)	Legionella gratiana HWL_155b HWH	1.161	45066
8 (-)	Corynebacterium singulare DSM 44357T DSM	1.158	161899
9 (-)	Enterococcus faecalis 20247_4 CHB	1.149	1351
10 (-)	Alishewanella fetalis DSM 16032T HAM	1.139	111143

Analyte205

Analyte Name:	I13
Analyte Description:	
Analyte ID:	C35
Analyte Creation Date/Time:	2019-12-04T13:54:32.401
Applied MSP Library(ies):	BDAL, Filamentous Fungi Library 1.0, Mycobacteria Library 1.0 (bead method), IVD, Listeria
Applied Taxonomy Tree:	

Rank (Quality)	Matched Pattern	Score Value	NCBI Identifier
1 (-)	Pseudomonas syringae ssp syringae LMG 1247T HAM	1.452	317
2 (-)	Corynebacterium flavescens DSM 20296T DSM	1.444	28028
3 (-)	Pseudomonas straminea CIP 106745T HAM	1.379	47882
4 (-)	Streptococcus sobrinus DSM 20742T DSM	1.366	1310
5 (-)	Streptococcus salivarius 0807M25049501 IBS	1.362	1304
6 (-)	Aspergillus fumigatus M02 RLH	1.357	123269315
7 (-)	Pseudomonas savastanoi ssp savastanoi LMG 5011 HAM	1.303	29438
8 (-)	Streptococcus salivarius DSM 20560T DSM	1.301	1304
9 (-)	Staphylococcus simulans DSM 20324 DSM	1.29	1286
10 (-)	Streptomyces lavendulae B264 UFL	1.282	1914

Analyte206

Analyte Name:	I14
Analyte Description:	
Analyte ID:	C35
Analyte Creation Date/Time:	2019-12-04T13:54:31.994
Applied MSP Library(ies):	BDAL, Filamentous Fungi Library 1.0, Mycobacteria Library 1.0 (bead method), IVD, Listeria
Applied Taxonomy Tree:	

Rank (Quality)	Matched Pattern	Score Value	NCBI Identifier
1 (++)	Streptococcus salivarius 0807M25049501 IBS	2.093	1304
2 (+)	Streptococcus vestibularis CCUG 51352 CCUG	1.773	1343
3 (+)	Streptococcus vestibularis CCUG 61229 CCUG	1.712	1343
4 (-)	Streptococcus macacae DSM 20724T DSM	1.588	1339
5 (-)	Streptococcus salivarius IBS_MS_23 IBS	1.522	1304
6 (-)	Bacillus farraginis DSM 16013T DSM	1.489	254757
7 (-)	Streptococcus suis DSM 9682T DSM	1.47	1307
8 (-)	Streptococcus cristatus DSM 8249T DSM	1.435	45634
9 (-)	Streptococcus suis GD69 GDD	1.418	1307
10 (-)	Streptococcus parasanguinis 14137939_2 MVD	1.412	1318

Analyte207

Analyte Name:	I15
Analyte Description:	
Analyte ID:	CN14A
Analyte Creation Date/Time:	2019-12-04T13:54:31.611
Applied MSP Library(ies):	BDAL, Filamentous Fungi Library 1.0, Mycobacteria Library 1.0 (bead method), IVD, Listeria
Applied Taxonomy Tree:	

Rank (Quality)	Matched Pattern	Score Value	NCBI Identifier
1 (+)	Staphylococcus aureus ssp aureus DSM 4910 DSM	1.865	46170
2 (+)	Staphylococcus aureus ATCC 29213 THL	1.839	1280
3 (+)	Staphylococcus aureus ssp aureus DSM 3463 DSM	1.702	46170
4 (-)	Staphylococcus aureus ssp aureus DSM 20231T DSM	1.641	46170
5 (-)	Staphylococcus aureus ATCC 33591 THL	1.565	1280
6 (-)	Staphylococcus aureus ssp aureus DSM 20232 DSM	1.558	46170
7 (-)	Staphylococcus aureus ATCC 33862 THL	1.54	1280
8 (-)	Staphylococcus aureus ATCC 25923 THL	1.529	1280
9 (-)	Staphylococcus aureus ssp aureus DSM 20652 DSM	1.503	46170
10 (-)	Lactobacillus zeae DSM 20178T DSM	1.394	57037

Analyte208

Analyte Name:	I16
Analyte Description:	
Analyte ID:	CN14A
Analyte Creation Date/Time:	2019-12-04T13:54:32.593
Applied MSP Library(ies):	BDAL, Filamentous Fungi Library 1.0, Mycobacteria Library 1.0 (bead method), IVD, Listeria
Applied Taxonomy Tree:	

Rank (Quality)	Matched Pattern	Score Value	NCBI Identifier
1 (-)	Staphylococcus aureus ssp aureus DSM 4910 DSM	1.624	46170
2 (-)	Clostridium bifermentans 2274_CCUG 35556 A BOG	1.437	1490
3 (-)	Candida parapsilosis ATCC 22019 THL	1.405	5480
4 (-)	Clostridium sordellii 1070_ATCC 9714T BOG	1.393	1505
5 (-)	Staphylococcus aureus ATCC 33591 THL	1.389	1280
6 (-)	Blastomonas ursincola DSM 9006T HAM	1.38	56361
7 (-)	Staphylococcus aureus ATCC 29213 THL	1.372	1280
8 (-)	Agromyces rhizospherae HKI 302_DSM 14597T HKJ	1.36	88374
9 (-)	Candida lambica CBS 603 CBS	1.347	53655
10 (-)	Pichia occidentalis CBS 1910 CBS	1.347	54552

Analyte209

Analyte Name:	I17
Analyte Description:	
Analyte ID:	CN14B
Analyte Creation Date/Time:	2019-12-04T13:54:31.810
Applied MSP Library(ies):	BDAL, Filamentous Fungi Library 1.0, Mycobacteria Library 1.0 (bead method), IVD, Listeria
Applied Taxonomy Tree:	

Rank (Quality)	Matched Pattern	Score Value	NCBI Identifier
1 (-)	Rothia dentocariosa CCUG 29965 CCUG	1.546	2047
2 (-)	Streptococcus salivarius DSM 20560T DSM	1.489	1304
3 (-)	Brevibacterium casei IMET 10997T HKJ	1.441	33889
4 (-)	Streptococcus vestibularis CCUG 51352 CCUG	1.381	1343
5 (-)	Rothia dentocariosa DSM 43762T DSM	1.353	2047
6 (-)	Clostridium cochlearium 1050_NCTC 2909 BOG	1.345	1494
7 (-)	Clostridium cochlearium 1080_ATCC 17794T BOG	1.336	1494
8 (-)	Paenibacillus macerans DSM 1574 DSM	1.335	44252
9 (-)	Lactobacillus gasseri DSM 20604 DSM	1.309	1596
10 (-)	Paenibacillus alvei DSM 29T DSM	1.284	44250

Analyte210

Analyte Name:	I18
Analyte Description:	
Analyte ID:	CN14B
Analyte Creation Date/Time:	2019-12-04T13:54:32.075
Applied MSP Library(ies):	BDAL, Filamentous Fungi Library 1.0, Mycobacteria Library 1.0 (bead method), IVD, Listeria
Applied Taxonomy Tree:	

Rank (Quality)	Matched Pattern	Score Value	NCBI Identifier
1 (-)	Paenibacillus phyllosphaerae DSM 17399T DSM	1.369	274593
2 (-)	Paenibacillus macerans DSM 1574 DSM	1.359	44252
3 (-)	Arthrobacter stackebrandtii DSM 16005T DSM	1.328	272161
4 (-)	Plesiomonas shigelloides DSM 8224T DSM	1.317	703
5 (-)	Agromyces italicus HKI 325_DSM 16388T HKJ	1.303	279572
6 (-)	Siccibacter colletis CCUG 51494 CCUG_corr	1.279	543
7 (-)	Ralstonia pickettii 21323_1 CHB	1.275	329
8 (-)	Corynebacterium confusum DSM 44384T DSM	1.254	71254
9 (-)	Agromyces bracchium HKI 303 DSM 14596T HKJ	1.251	88376
10 (-)	Lactobacillus plantarum DSM 20205 DSM	1.245	1590

Analyte211

Analyte Name:	I19
Analyte Description:	
Analyte ID:	CN14C
Analyte Creation Date/Time:	2019-12-04T13:54:32.014
Applied MSP Library(ies):	BDAL, Filamentous Fungi Library 1.0, Mycobacteria Library 1.0 (bead method), IVD, Listeria
Applied Taxonomy Tree:	

Rank (Quality)	Matched Pattern	Score Value	NCBI Identifier
1 (-)	Lactobacillus kefiri DSM 20588 DSM	1.377	33962
2 (-)	Acidovorax avenae ssp citrulli LMG 5376T HAM	1.353	80869
3 (-)	Agromyces bracchium HKI 303 DSM 14596T HKJ	1.348	88376
4 (-)	Brevibacterium casei IMET 10997T HKJ	1.348	33889
5 (-)	Lactobacillus plantarum DSM 20205 DSM	1.342	1590
6 (-)	Agromyces salentinus HKI 320_DSM 16198T HKJ	1.317	269421
7 (-)	Arthrobacter pyridinolis B384 UFL	1.31	1663
8 (-)	Lactobacillus saerimneri DSM 16027 DSM	1.309	228229
9 (-)	Streptococcus pyogenes ATCC 19615 THL	1.296	1314
10 (-)	Lactobacillus sharpeae DSM 20506 DSM	1.295	1626

Analyte212

Analyte Name:	I20
Analyte Description:	
Analyte ID:	CN14C
Analyte Creation Date/Time:	2019-12-04T13:54:32.731
Applied MSP Library(ies):	BDAL, Filamentous Fungi Library 1.0, Mycobacteria Library 1.0 (bead method), IVD, Listeria
Applied Taxonomy Tree:	

Rank (Quality)	Matched Pattern	Score Value	NCBI Identifier
1 (-)	Lactobacillus kefiri DSM 20587T DSM	1.517	33962
2 (-)	Streptococcus macacae DSM 20724T DSM	1.435	1339
3 (-)	Rothia dentocariosa DSM 43762T DSM	1.397	2047
4 (-)	Clostridium bifermentans 2273_CCUG 35297 BOG	1.356	1490
5 (-)	Rothia dentocariosa RV_BA1_032010_D LBK	1.329	2047
6 (-)	Streptococcus sobrinus DSM 20742T DSM	1.307	1310
7 (-)	Weissella viridescens DSM 20248 DSM	1.304	1629
8 (-)	Pseudomonas congelans DSM 14939T HAM	1.273	200452
9 (-)	Staphylococcus warneri DSM 20036 DSM	1.268	1292
10 (-)	Lactobacillus saerimneri DSM 16027 DSM	1.254	228229

Analyte213

Analyte Name:	I21
Analyte Description:	
Analyte ID:	CN24A
Analyte Creation Date/Time:	2019-12-04T13:54:32.314
Applied MSP Library(ies):	Listeria, BDAL, Filamentous Fungi Library 1.0, Mycobacteria Library 1.0 (bead method), IVD
Applied Taxonomy Tree:	

Rank (Quality)	Matched Pattern	Score Value	NCBI Identifier
1 (+)	Rothia mucilaginosa BK2995_09 ERL	1.773	43675
2 (-)	Rothia dentocariosa CCUG 29965 CCUG	1.668	2047
3 (-)	Rothia mucilaginosa DSM 20446 DSM	1.638	43675
4 (-)	Rothia dentocariosa RV_BA1_032010_D LBK	1.591	2047
5 (-)	Rothia dentocariosa G6496_ch28 IBS	1.53	2047
6 (-)	Rothia mucilaginosa CCUG 52532 CCUG	1.528	43675
7 (-)	Rothia dentocariosa B16575_bh8 IBS	1.524	2047
8 (-)	Rothia mucilaginosa CCUG 44966 CCUG	1.51	43675
9 (-)	Rothia mucilaginosa DSM 20746T DSM	1.495	43675
10 (-)	Streptococcus gordonii DSM 6777T DSM	1.431	1302

Analyte214

Analyte Name:	I22
Analyte Description:	
Analyte ID:	CN24A
Analyte Creation Date/Time:	2019-12-04T13:54:32.247
Applied MSP Library(ies):	BDAL, Filamentous Fungi Library 1.0, Mycobacteria Library 1.0 (bead method), IVD, Listeria
Applied Taxonomy Tree:	

Rank (Quality)	Matched Pattern	Score Value	NCBI Identifier
1 (++)	Rothia dentocariosa CCUG 29965 CCUG	2.016	2047
2 (+)	Rothia mucilaginosa BK2995_09 ERL	1.947	43675
3 (+)	Rothia dentocariosa RV_BA1_032010_D LBK	1.913	2047
4 (+)	Rothia dentocariosa B16575_bh8 IBS	1.879	2047
5 (+)	Rothia mucilaginosa DSM 20445 DSM	1.798	43675
6 (+)	Rothia dentocariosa G6496_ch28 IBS	1.791	2047
7 (+)	Rothia mucilaginosa CCUG 44966 CCUG	1.787	43675
8 (+)	Rothia mucilaginosa CCUG 52532 CCUG	1.727	43675
9 (-)	Rothia mucilaginosa DSM 20446 DSM	1.676	43675
10 (-)	Rothia mucilaginosa DSM 20446 BRB	1.657	43675

Analyte215

Analyte Name:	I23
Analyte Description:	
Analyte ID:	CN24B
Analyte Creation Date/Time:	2019-12-04T13:54:32.061
Applied MSP Library(ies):	BDAL, Filamentous Fungi Library 1.0, Mycobacteria Library 1.0 (bead method), IVD, Listeria
Applied Taxonomy Tree:	

Rank (Quality)	Matched Pattern	Score Value	NCBI Identifier
1 (-)	Brevibacterium casei IMET 10997T HKJ	1.508	33889
2 (-)	Streptococcus lutetiensis DSM 15350T DSM	1.489	150055
3 (-)	Propionibacterium thoenii DSM 20276T DSM	1.484	1751
4 (-)	Weissella viridescens DSM 20248 DSM	1.475	1629
5 (-)	Streptococcus downei DSM 5635T DSM	1.453	1317
6 (-)	Rothia dentocariosa DSM 43762T DSM	1.427	2047
7 (-)	Propionibacterium thoenii DSM 20277 DSM	1.388	1751
8 (-)	Streptococcus salivarius 0807M25049501 IBS	1.385	1304
9 (-)	Streptococcus vestibularis CCUG 61229 CCUG	1.38	1343
10 (-)	Streptococcus orisratti DSM 15617T DSM	1.357	114652

Analyte216

Analyte Name:	I24
Analyte Description:	
Analyte ID:	CN24B
Analyte Creation Date/Time:	2019-12-04T13:54:32.680
Applied MSP Library(ies):	BDAL, Filamentous Fungi Library 1.0, Mycobacteria Library 1.0 (bead method), IVD, Listeria
Applied Taxonomy Tree:	

Rank (Quality)	Matched Pattern	Score Value	NCBI Identifier
1 (+)	Streptococcus salivarius 0807M25049501 IBS	1.756	1304
2 (-)	Streptococcus salivarius DSM 20560T BRB	1.613	1304
3 (-)	Streptococcus salivarius IBS_MS_23 IBS	1.578	1304
4 (-)	Streptococcus salivarius DSM 20560T DSM	1.577	1304
5 (-)	Streptococcus sobrinus DSM 20742T DSM	1.519	1310
6 (-)	Streptococcus downei DSM 5635T DSM	1.487	1317
7 (-)	Rothia dentocariosa DSM 43762T DSM	1.467	2047
8 (-)	Streptococcus alactolyticus DSM 5199 DSM	1.407	29389
9 (-)	Streptococcus vestibularis DSM 5636T DSM	1.402	1343
10 (-)	Weissella viridescens DSM 20248 DSM	1.372	1629

Analyte217

Analyte Name:	J1
Analyte Description:	
Analyte ID:	CN8A
Analyte Creation Date/Time:	2019-12-04T13:54:32.575
Applied MSP Library(ies):	BDAL, Filamentous Fungi Library 1.0, Mycobacteria Library 1.0 (bead method), IVD, Listeria
Applied Taxonomy Tree:	

Rank (Quality)	Matched Pattern	Score Value	NCBI Identifier
1 (+)	Rothia dentocariosa DSM 43762T DSM	1.902	2047
2 (-)	Rothia mucilaginosa CCUG 52532 CCUG	1.58	43675
3 (-)	Rothia mucilaginosa CCUG 44966 CCUG	1.411	43675
4 (-)	Rothia dentocariosa RV_BA1_032010_D LBK	1.407	2047
5 (-)	Rothia mucilaginosa BK2995_09 ERL	1.386	43675
6 (-)	Rothia dentocariosa CCUG 29965 CCUG	1.294	2047
7 (-)	Arthrobacter oxydans DSM 20119T DSM	1.274	1671
8 (-)	Corynebacterium pilosum DSM 20521T DSM	1.256	35756
9 (-)	Burkholderia cepacia LMG 2161 HAM	1.254	292
10 (-)	Pseudomonas koreensis LMG 21318T HAM	1.21	198620

Analyte218

Analyte Name:	J2
Analyte Description:	
Analyte ID:	CN8A
Analyte Creation Date/Time:	2019-12-04T13:54:32.650
Applied MSP Library(ies):	BDAL, Filamentous Fungi Library 1.0, Mycobacteria Library 1.0 (bead method), IVD, Listeria
Applied Taxonomy Tree:	

Rank (Quality)	Matched Pattern	Score Value	NCBI Identifier
1 (+)	Rothia dentocariosa DSM 43762T DSM	1.736	2047
2 (-)	Rothia dentocariosa CCUG 29965 CCUG	1.677	2047
3 (-)	Bifidobacterium saeculare DSM 6532 DSM	1.365	78257
4 (-)	Arthrobacter roseus DSM 14508T DSM	1.321	136274
5 (-)	Lactobacillus johnsonii DSM 20553 DSM	1.296	33959
6 (-)	Rothia mucilaginosa DSM 20746T DSM	1.268	43675
7 (-)	Corynebacterium flavescens IMET 11080T HKJ	1.265	28028
8 (-)	Streptococcus macacae DSM 20724T DSM	1.232	1339
9 (-)	Weissella viridescens DSM 20248 DSM	1.223	1629
10 (-)	Burkholderia pyrrocinia LMG 14191T HAM	1.193	60550

Analyte219

Analyte Name: J3
Analyte Description:
Analyte ID: CN8B
Analyte Creation Date/Time: 2019-12-04T13:54:32.475
Applied MSP Library(ies): BDAL, Filamentous Fungi Library 1.0, Mycobacteria Library 1.0 (bead method), IVD, Listeria
Applied Taxonomy Tree:

Rank (Quality)	Matched Pattern	Score Value	NCBI Identifier
1 (+)	Rothia dentocariosa CCUG 29965 CCUG	1.713	2047
2 (-)	Rothia dentocariosa DSM 43762T DSM	1.514	2047
3 (-)	Rothia dentocariosa RV_BA1_032010_D LBK	1.436	2047
4 (-)	Weissella viridescens DSM 20248 DSM	1.42	1629
5 (-)	Streptococcus vestibularis DSM 5636T DSM	1.388	1343
6 (-)	Weissella minor DSM 20014T DSM	1.384	1620
7 (-)	Streptococcus downei DSM 5635T DSM	1.278	1317
8 (-)	Staphylococcus aureus ssp aureus DSM 3463 DSM	1.272	46170
9 (-)	Arthrobacter oxydans DSM 20119T DSM	1.263	1671
10 (-)	Rothia dentocariosa G6496_ch28 IBS	1.262	2047

Analyte220

Analyte Name:	J4
Analyte Description:	
Analyte ID:	CN8B
Analyte Creation Date/Time:	2019-12-04T13:54:32.358
Applied MSP Library(ies):	BDAL, Filamentous Fungi Library 1.0, Mycobacteria Library 1.0 (bead method), IVD, Listeria
Applied Taxonomy Tree:	

Rank (Quality)	Matched Pattern	Score Value	NCBI Identifier
1 (-)	Rothia dentocariosa DSM 43762T DSM	1.492	2047
2 (-)	Filifactor villosus 1051_NCTC 11220T BOG	1.435	29374
3 (-)	Streptococcus salivarius DSM 20560T DSM	1.378	1304
4 (-)	Paenibacillus phyllosphaerae DSM 17399T DSM	1.374	274593
5 (-)	Paenibacillus sp DSM 1482 DSM	1.363	44249
6 (-)	Arthrobacter parietis DSM 16404T DSM	1.342	271434
7 (-)	Paenibacillus cineris DSM 16945T DSM	1.34	237530
8 (-)	Weissella viridescens DSM 20248 DSM	1.332	1629
9 (-)	Pseudomonas syringae ssp syringae DSM 6693 HAM	1.308	317
10 (-)	Paenibacillus terrae DSM 15891 DSM	1.306	159743
